# How does creative enquiry enable us to deal with uncertainty?

**DOI:** 10.1186/s12909-026-10060-w

**Published:** 2026-08-08

**Authors:** Ivon Kandiah, Louise Younie

**Affiliations:** Queen Mary University of London, London, UK

**Keywords:** Creative enquiry, Uncertainty, Medical education, Clinical practice

## Abstract

**Background:**

Uncertainty is an inherent and pervasive feature of medical practice. Here, uncertainty refers to the subjective perception and affective experience encountered by medical students and clinicians. Difficulties in managing uncertainty are associated with impaired communication in shared decision-making and may contribute to clinical burnout. Despite longstanding recognition of its importance, including recent UK curricular reforms focused on “managing uncertainty,” it remains insufficiently addressed in medical practice. This study seeks to address this gap by examining how creative enquiry may support both learners and clinicians in engaging with uncertainty in practice.

**Methods:**

We undertook a qualitative study using semi-structured interviews with ten participants (five medical students; five clinicians, comprising four general practitioners and one infectious disease specialist) with prior experience of creative enquiry. Participants were recruited through convenience sampling from a creative-enquiry student-selected component (SSC) and clinician networks. Interviews were conducted online, audio-recorded, and transcribed verbatim. Data were analysed inductively using Braun and Clarke’s reflexive thematic analysis, supported by NVivo 14. Student participants had completed the SSC in preclinical years and were interviewed during clinical training. Creative enquiry activities included engagement with patient-generated creative work, arts-based practices (e.g. photography, collage, creative writing, sculpture), and facilitated group dialogue.

**Results:**

Three analytically related dimensions were generated: Issues, Processes, and Effects. Participants described uncertainty as intensified by binary epistemic cultures and the marginalisation of emotional experience. Creative enquiry involved engagement with indeterminate processes characterised by the absence of singular correct outcomes and the unpredictability of creative production. These processes enabled emotional processing, supporting participants to “digest” affective responses, and fostered a stance of “permission to play,” legitimising experimentation and imperfection. Repeated engagement contributed to the normalisation of uncertainty, with parallels drawn to exposure.

Reported effects included restoration of voice and recognition, reduction in the emotional burden of uncertainty, and enhanced reflexivity. Participants described an expanded capacity for perspective-taking and more complex conceptualisations of practice (“seeing medicine in three dimensions”).

**Conclusions:**

This study extends current understandings of uncertainty in medical education by identifying creative enquiry as a practice that integrates epistemic and affective engagement. We propose a Personal, Protective, and Embracing (PPE) framework, through which creative enquiry supports (i) articulation and emotional processing (Personal), (ii) containment and respite (Protective), and (iii) acceptance and constructive engagement with uncertainty (Embracing). These findings contribute to the operationalisation of “managing uncertainty” as a curricular competency and support further theoretically informed and empirical investigation of creative pedagogies in medical education.

**Supplementary Information:**

The online version contains supplementary material available at https://doi.org/10.1186/s12909-026-10060-w.

## Background

Medicine has long been described as “a science of uncertainty and an art of probability” [1]. This characterisation captures a persistent feature of practice that has been recognised from Hippocratic medicine to the present day [2, 3]. Uncertainty is not peripheral but woven through everyday clinical work. It appears in diagnostic reasoning, in conversations with patients, and in the organisation of care [4]. Advances in science and technology have not removed this condition. Instead, they have often exposed further areas of indeterminacy and extended the grey zones within which clinicians must act [5]. At the same time, professional cultures that are uneasy with acknowledging uncertainty continue to shape practice, with implications for both quality and cost of care [6]. Recent developments in the United Kingdom reflect a shift in this regard. The Medical Licensing Assessment (MLA) content map and the Royal College of General Practitioners (RCGP) handbook now identify managing uncertainty as a core capability across undergraduate and postgraduate training.

Across the history of medicine, practitioners have drawn on a range of resources to respond to uncertainty. These include art, philosophy, reflective practice, and dialogue with colleagues [7–9]. Within medical education, the arts and humanities have similarly evolved. What was once positioned as enrichment has become more closely aligned with enquiry [10–13]. Learners are increasingly invited to make, interpret, and share creative work as a way of exploring clinical and personal experience [14, 15]. The terms “creative inquiry”, “arts based inquiry”, and “creative enquiry” are often used interchangeably in this literature [16, 17]. In this study, we use “creative enquiry” to denote the exploration of lived experience through artistic practice [17].

Uncertainty itself has been defined in multiple ways. Early work dating back to the 1950 s established its subjective character [18, 19]. Mishel described uncertainty as arising when individuals cannot make sense of events because available cues are insufficient or unclear [20]. Subsequent authors have reinforced this emphasis on uncertainty as a lived and personal experience [21–23]. Beresford, drawing on interviews with clinicians, distinguished between technical uncertainty, where scientific knowledge is incomplete, personal uncertainty, where patient preferences are not known, and conceptual uncertainty, where general principles are difficult to apply in specific situations [24]. Other accounts highlight that uncertainty is not only cognitive but also behavioural and emotional in its effects [21, 22]. Han et al. brought these strands together in a three dimensional taxonomy that identifies sources, issues, and loci of uncertainty in clinical practice [25]. They describe probability, ambiguity, and complexity as key sources, while also distinguishing between disease centred, system centred, and patient centred concerns. In this study, we adopt Lee et al.’s definition of uncertainty as “the dynamic, subjective perception of not knowing what to think, feel, or do” [26]. Ambiguity is treated as a related but distinct construct, understood as the presence of multiple possible interpretations rather than as a direct synonym for uncertainty.

Recent reviews have extended these definitions and explored how uncertainty is experienced and addressed in contemporary education and practice [4, 26–28]. Moffett et al. describe uncertainty among undergraduate health professions students as a felt sense of not knowing that prompts cognitive, behavioural, and emotional responses [4]. Patel et al. map educational interventions aimed at shaping these responses, drawing explicitly on Lee et al.’s framework [26, 27]. Gardner et al. show how students and trainees encounter uncertainty in primary care settings, where incomplete information and competing demands are routine [28]. At the same time, uncertainty remains contested. Nowotny argues that it is not simply a deficit to be controlled but a condition embedded in ambiguity, risk, and regulation. On this view, uncertainty is something that must often be engaged with, and at times embraced, rather than resolved [29].

For clinicians, uncertainty is frequently experienced as a moment of indecision about what to think, feel, or do [22]. The ability to act under such conditions is central to professional practice [8, 30]. When this capacity is limited, the consequences can be significant. Poor decision making, over investigation, and emotional strain have all been linked to difficulties in responding to uncertainty [31–33]. Developing this capacity has therefore been described as a pressing priority for medical education, even characterised as “The Next Medical Revolution” [34].

At the same time, there is evidence that educational environments do not always support this development. Moffett et al. highlight a tension between the human desire for certainty and the realities of clinical work [4]. Where uncertainty is treated as error or failure, learners may avoid acknowledging it. Such environments can leave clinicians ill prepared for the complexity and unpredictability of practice [34–36].

Within this context, creative enquiry has been proposed as one way of engaging with uncertainty. Patel et al.’s review of twenty four studies identified six that used creative approaches [27]. Although quantitative findings were mixed, all six reported some positive contribution. In Moffett et al.’s review, two studies found a statistically significant positive effect of creative approaches on responses to uncertainty [4, 37, 38]. These findings suggest that the value of creative enquiry may lie not only in measurable outcomes but also in the qualities of the process itself. Three features are particularly relevant. First, creative work is inherently uncertain. Second, it can widen perspectives and disrupt habitual ways of seeing. Third, it is grounded in subjective and affective experience. In this sense, creative enquiry appears to have a dual relationship with uncertainty. It may generate uncertainty through open ended processes, and it may also support engagement with uncertainty through reflection and meaning making.

This dual relationship is widely recognised. Montouri compares creativity to opening Pandora’s box, emphasising both its unpredictability and its generative potential [16]. Beghetto and Henriksen similarly argue that uncertainty is not an obstacle to creativity but a condition of it [39, 40]. Engagement with the arts can encourage careful looking, multiple interpretations, and new forms of understanding in situations that resist simple answers [41–45]. By contrast, medical education has often prioritised certainty, emphasising factual knowledge and correct answers [42]. Creative enquiry introduces a different orientation. It invites learners to pause, to look again, and to consider alternative meanings. In doing so, it may enable them to revisit difficult situations and to see them differently [42, 43, 45]. It also provides a space in which emotions can be acknowledged and explored. This is important because uncertainty is often felt as much as it is thought [43, 45, 46]. As Lawrence suggests, creative practice allows individuals to “be with” their emotions rather than move quickly to resolve them [46]. Emotions are closely linked to both creativity and uncertainty, shaping how situations are perceived and acted upon [23, 33]. Although these features of creative enquiry are recognised, the ways in which they shape engagement with uncertainty in medical education remain insufficiently understood.

More recent work has called for approaches to uncertainty that move beyond communication strategies alone [47]. Creative enquiry may offer one such approach. However, its role should not be overstated. It is not simply a tool for reducing uncertainty. Rather, it may also intensify uncertainty by opening up new questions and unsettling established assumptions. In this sense, creative enquiry can both challenge and support learners as they encounter uncertainty. Against this background, this study explores how students and clinicians with experience of creative enquiry describe its relationship to uncertainty in their own accounts of practice and training.

## Methods

### Study design and setting

We conducted an exploratory qualitative study informed by an interpretivist orientation [48–50]. This approach assumes that knowledge is produced through interpretation and is shaped by context, experience, and social position, rather than simply discovered as an objective reality. It was therefore well suited to our aim: to explore how medical students and clinicians with experience of creative enquiry understood its relationship to uncertainty in medical education and clinical practice.

A qualitative design was chosen because we were not seeking to test the effectiveness of a defined intervention or to establish causal effects. Rather, we wished to examine how participants themselves described, interpreted, and made sense of this relationship. This was particularly important given the conceptual issues raised in the literature. Creative enquiry may not simply help people respond to uncertainty; it may also intensify or expose uncertainty through open ended, interpretive, and affectively charged processes. An exploratory design therefore allowed us to investigate participants’ accounts without assuming in advance that creative enquiry functioned in one fixed way.

### Participants

Participants were recruited through convenience sampling. The sample comprised ten participants: five medical students from Queen Mary University of London who had previously elected to take a creative enquiry Student Selected Component in Year 1 or Year 2, and five clinicians with prior experience of creative enquiry, including four general practitioners and one infectious disease specialist. All participants gave written informed consent before taking part. No incentives were offered, and participants were free to withdraw at any stage.

Student participants had completed the SSC in their early years of study but were interviewed later, after entering the clinical phase of training and gaining experience of patient care. Including both students and practising clinicians enabled us to explore how creative enquiry was understood across different stages of professional formation. At the same time, we recognise that these groups brought different levels of clinical experience and authority to their accounts.

General practitioners were included because uncertainty is a familiar and explicit feature of general practice, and because the Royal College of General Practitioners identifies engagement with uncertainty as an important aspect of learning and practice. The inclusion of one infectious disease specialist broadened the sample beyond primary care and highlighted that uncertainty is not confined to one specialty or setting.

This sampling strategy was used because the study was exploratory and because the pool of individuals with direct experience of the specific creative enquiry course and related practices was limited. However, this approach has important consequences for interpretation. Participants were recruited from groups already disposed towards creative practice or interested in it. Their accounts therefore cannot be taken as representative of medical students or clinicians more broadly, nor do they allow claims about the views of those who are sceptical of creative enquiry or have not engaged with it. We treat the findings as situated accounts from a self selecting sample, not as evidence of generalisable effects.

### Recruitment

Recruitment was facilitated by L.Y., who acted as gatekeeper to three groups: the Creative Enquiry Student Selected Component at Queen Mary University of London, the Royal College of General Practitioners Creative Health Special Interest Group, and a WhatsApp group for clinicians concerned with flourishing in practice. Potential participants were approached through digital posters distributed by email and WhatsApp, and through personal invitation at a symposium on arts in medical education (Appendix A).

Those who expressed interest were sent a participant information sheet (Appendix B) and consent form (Appendix C). Interviews invited reflection on personal and professional experience and therefore had the potential to surface difficult or sensitive material. For that reason, participants were offered access to support through L.Y. should distress arise, although this did not prove necessary.

### Data collection

We used semi structured interviews to generate rich accounts while retaining enough flexibility to follow participants’ own concerns and examples [51, 52]. The interview guide (Appendix D) was developed by I.K. in discussion with L.Y. and designed to prompt reflection on experiences of uncertainty, experiences of creative enquiry, and perceived relationships between the two. Interviews were conducted online using Microsoft Teams.

Interviews lasted an average of 41 min and were audio recorded using the platform’s recording function. Microsoft Teams generated a live transcript for each interview, but these transcripts were checked manually against the audio recordings, corrected where necessary, and formatted by I.K. to ensure accuracy. The final dataset comprised approximately 80 pages of transcript.

Participants described a range of creative enquiry activities. These included engagement with patient creative work, participation in arts based practices such as photography, collage, creative writing, and sculpture, the sharing of creative outputs, and group discussion. These experiences were not standardised and did not constitute a single intervention. Rather, they formed a set of related practices centred on artistic making, interpretation, and reflection.

### Ethics and data management

The study received approval from the Institute of Health Sciences Education Peer Review Panel at Queen Mary University of London and from the Queen Mary University of London Devolved School Research Ethics Committee (reference IPREC231213.KAN; Appendix E). Data were collected and stored in accordance with the Data Protection Act (1998) and institutional policy on password protected devices [53].

The study was conducted in line with the principles of the Declaration of Helsinki. All participants provided written informed consent to participate and gave explicit consent for the use of anonymised quotations in publications arising from the study.

### Data analysis

Analysis was conducted by I.K., a medical student and researcher in medical education, and L.Y., a professor of medical education and physician educator with more than 20 years’ experience in creative enquiry within medical education. We analysed the interview data inductively using Braun and Clarke’s six phase approach to thematic analysis [54]. This method was chosen because it offers a systematic but flexible way of identifying patterns of meaning across qualitative data while remaining compatible with an interpretivist approach.

The analysis began with immersion in the data through repeated listening to recordings and repeated reading of transcripts. Initial codes were then generated across the dataset. Coding attended both to semantic content, what participants directly said, and to latent meaning, what their accounts suggested at a more interpretive level. For example, explicit descriptions of medicine as “black and white” were coded semantically, while broader ideas such as “voice” or “permission” were coded at a latent level. Related codes were then grouped and reviewed to develop candidate themes. An example of this process was the merging of codes such as “right or wrong” and “black and white” into the broader theme of binary thinking.

Themes were reviewed in relation to the entire dataset, refined for coherence and distinctiveness, and then defined in relation to the research question. The final analytic account was developed through discussion between both authors and supported using NVivo 14. Across January to May 2023, I.K. and L.Y. met regularly online to review transcripts, compare coding, and discuss the development of themes.

The analysis generated approximately 70 initial codes, which were progressively refined into a smaller set of final themes. As with all interpretive qualitative work, theme development was shaped by the interaction between dataset, analytic judgement, and researcher perspective. We therefore present the analysis not as a definitive account of creative enquiry and uncertainty, but as a careful interpretation of how this particular group of participants described their experiences.

To enhance analytic rigour, we used collaborative discussion throughout coding and theme development. We also sought feedback from doctors with experience of creative enquiry and from students who had completed the course. These conversations suggested that the developing interpretation resonated with their experiences. We treat this as supportive of credibility, while recognising that it does not remove the limitations associated with the sample or the interpretive nature of the analysis.

## Results: overview

Interviews with five clinicians and five medical students produced detailed accounts of how creative enquiry related to uncertainty in practice and training. Several participants commented that the interview itself had been reflective and useful. As one clinician remarked, “Thanks for the free therapy” (Doctor 5). Another said, “I think we covered everything. But it’s very stimulating, Ivon, to talk about this. I need to write some of this down properly” (Doctor 2).

**Fig. 1:**
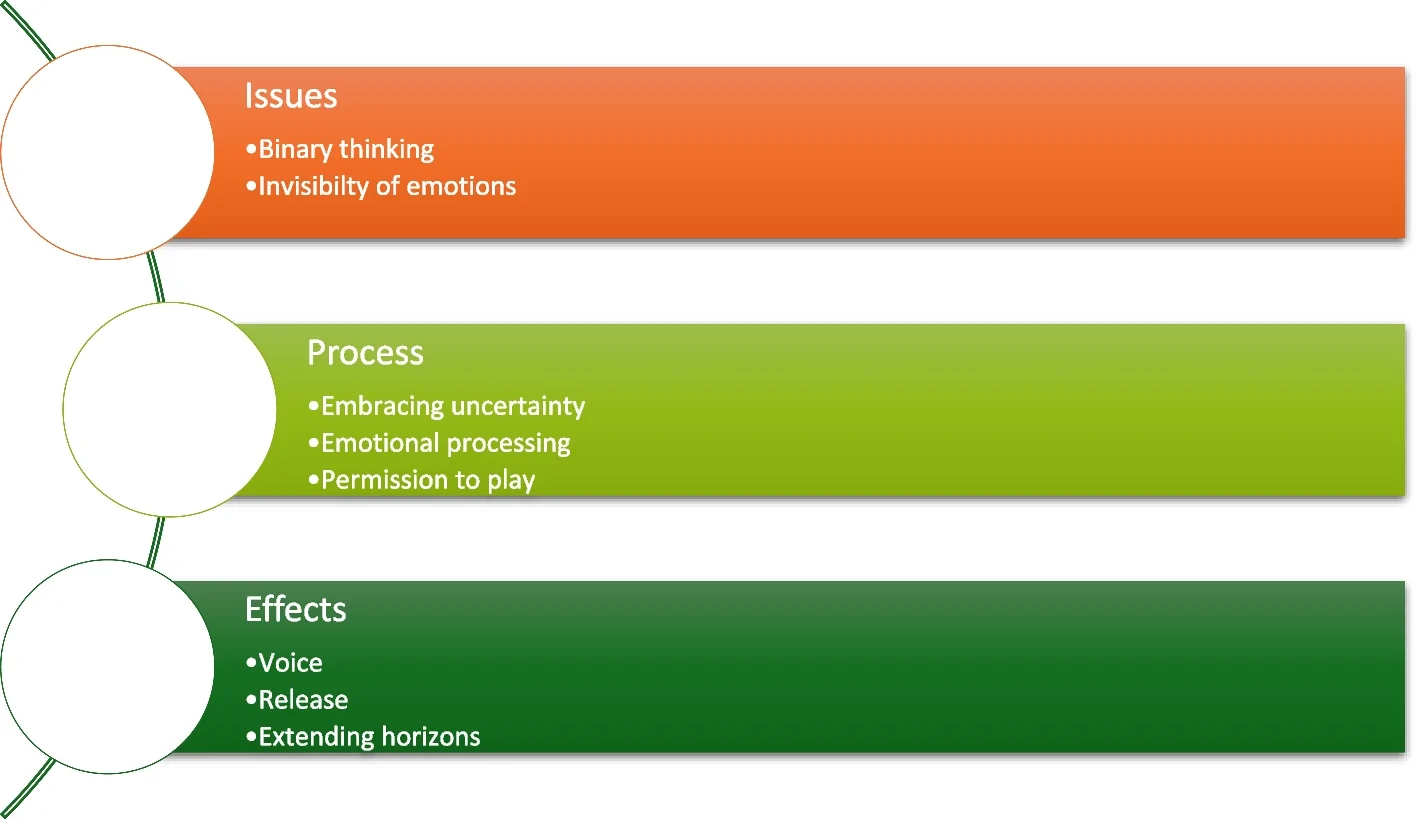
Three dimensions of results

The analysis generated three interrelated dimensions: first, issues that intensified uncertainty (binary thinking and invisibility of emotions); second, processes within creative enquiry through which participants engaged with uncertainty (i.e., embracing uncertainty, emotional processing, and permission to play); and third, perceived effects on clinical practice (voice, release, and extending horizons) (see Fig. 1). These dimensions should be understood as connected rather than sequential. Participants often moved between them, describing how features of medical culture shaped their experience of uncertainty, how creative enquiry opened a different mode of engagement, and how this in turn influenced their thinking and practice.

### Issues that exacerbate uncertainty

This dimension captures features of medical education and practice that participants felt heightened uncertainty. Two recurrent concerns were binary ways of thinking and the limited space available for emotional life in clinical settings.

#### Binary thinking

Participants often described medicine as organised around binary expectations. Doctor 3 characterised it as “very binary.” Four participants (Students 3 and 5; Doctors 4 and 5) referred to a culture of “right or wrong,” and two (Doctors 3 and 5) spoke of medicine in terms of “black or white.” As Doctor 4 explained, “there’s an obsession in medicine for right or wrong. […] For students it may be OSCEs and for doctors it may be a certain way you act.”

For some, this way of thinking was linked to forms of training that prioritised scientific certainty and algorithmic reasoning. Although these approaches were seen as necessary, they were also described as inadequate preparation for the ambiguity of real clinical work. Doctors 4 and 5 both suggested that such training did not equip clinicians for the “messiness” and “murky waters” of care. In these accounts, uncertainty was intensified when participants felt compelled to act within rigid frames that did not match the complexity of lived practice.

#### Invisibility and suppression of emotions

Most participants also described emotions as hidden, muted, or displaced within medicine. Student 2, reflecting on the uncertainty of illness through poetry, spoke of a “brush of grief.” Student 3 said that emotions are often “brush[ed] under the carpet.” Others (Student 3; Student 5; Doctor 5) described a fast paced environment that left “no time” to process feelings.

This was not framed simply as a matter of individual discomfort. Participants linked the suppression of emotion to wider structures of care and to professional expectations. Doctor 4 observed that “Often the doctors are invisible in care.” Student 1 made a similar point: “the focus is on the patient, the doctors are invisible.” For some, this invisibility was intensified by the pressures of the wider health system. Four participants (Student 3; Doctors 1, 2, and 4) explicitly referred to the state of the NHS. Doctor 1 described it as “broken,” while Doctor 2 said that “NHS England wants to take the feelings out of medicine, so we become like robots.” Student 3 added that many doctors were not appreciated and could feel “you’re not really an individual, you’re just kind of a number.”

Taken together, these accounts suggest that uncertainty was not experienced solely as a problem of incomplete knowledge. It was also bound up with emotional containment, organisational pressure, and professional cultures that left little room for complexity.

### Processes of creative enquiry

Participants described three main processes through which creative enquiry shaped their engagement with uncertainty: embracing uncertainty, emotional processing, and permission to play. These were not discrete stages. Rather, they represented overlapping ways in which creative enquiry offered a different mode of attending to uncertain experience.

#### Embracing uncertainty

Most participants described creative enquiry itself as inherently uncertain. Student 5 called it an “uncertain medium,” while Student 4 said that “being creative is being uncertain as well.” Several participants (Students 1 to 3; Doctor 4) referred to the creative process as “uncertain,” and Student 3 described it as “out of your hands.” Doctor 3 and Student 5, both of whom worked with sculpture, explained that the final piece is “never going to be exactly” what had first been imagined. For Student 5, this uncertainty was not only tolerable but sometimes “more exciting than daunting.”

Four participants (Students 3 and 5; Doctors 4 and 5) described creative enquiry as a space with “no right or wrong.” For Doctors 4 and 5, this stood in clear contrast to medicine’s “black and white” culture. Doctor 3 suggested that creative enquiry revealed “a lot more nuance to life and patients, and that there’s a lot of grey areas even within guidelines.” In this sense, creative enquiry did not remove uncertainty. It exposed participants to it in a different register, one that was less punitive and more exploratory.

Two participants explicitly used the language of “exposure therapy” to describe this process. Doctor 4 explained that “revisiting that same uncertain process, then eventually more negative feelings associated with that (uncertainty) will diminish.” Student 4 similarly described creative work as practice in placing feelings into an uncertain medium, so that one becomes more “OK” with tolerating uncertainty. Students 1 and 2 also described becoming more comfortable with uncertainty. For Student 2, this made it possible to “not shy away from it.” Student 3 described using creative work when she felt most stressed and uncertain. For her, the process became a way of moving towards uncertainty rather than away from it: “the more we embrace it, the more we become at peace with it.”

#### Emotional processing

A second process concerned emotion. Participants often described creative enquiry as a way of slowing down, attending to feeling, and making sense of experience. Some used the language of catharsis. Student 3 said that “emotions are interwoven with creativity. […] By using creative mediums, we understand the emotions behind it.” Students 3 and 5 described this link as “intrinsic.”

Several participants (Doctor 4; Students 4 and 5) said that creative enquiry helped them to “slow down.” Doctor 4 described it as space to “unpick,” while Student 4 said it prevented thoughts from going round “a 100 times over” and from “floating around in pieces” in the mind. Others (Students 1 and 4; Doctor 4) emphasised the value of having time to “sit” with emotions. Student 2 and Doctors 3 and 5 described a state of immersion or flow. Student 2, writing poetry, spoke of “spending time with the paper like without distractions” until “you come away and you’ve got a sense of like, ‘Oh’.” Doctor 3 described creative enquiry as “fully concentrating on (your) emotions” and learning to “digest your emotions.” Doctor 5 said it helped to “make sense of the situation.” Student 2 added that this kind of processing “actually allows you to grow.”

One participant, however, also pointed to possible risk. Doctor 5 cautioned that if one “open[s] the box” without preparation, “all the darkness spills out.” This suggests that creative enquiry was not universally experienced as benign or uncomplicated, even where it was valued.

Two participants, Student 3 and Doctor 2, also described points of contact between creative enquiry and spirituality. For both, faith was part of how uncertainty was processed and accepted. Doctor 2 used the image of an ocean: “Maybe we’re talking about the divine, Ivon. […] You want to put your emotions in there and actually that ocean is God.” Student 3 described a “creative spirit” and said that when uncertainty felt “out of your hands,” faith made it possible to place this “more in God’s hands,” which gave “a lot of peace.” She added that her faith had taught her to recognise uncertainty as part of life.

#### Permission to play

More than half of the participants described creative enquiry as playful, improvisational, or experimental. Doctor 4 linked play with something often lost after childhood. Student 2 similarly described creative enquiry as a form of “child like enjoyment” and said that “creativity is play. And human beings need that.” Student 3 suggested that play was not optional but built into the process: “you have to play.”

This playfulness was closely tied to imperfection. Doctor 1 described creative enquiry as “permission to think illogically,” as “the opposite of perfectionism,” and as a willingness “to fail and be stupid,” which she found “quite refreshing.” Student 4 reflected that without writing poetry she “would never have linked my own academics and my own feelings of perfectionism.” Doctor 4 similarly emphasised that the creative process is “totally a process for you” and “doesn’t have to be a work of art.” Student 3 described deliberately choosing unfamiliar media in order to “experiment,” which in turn “makes me feel I can make braver choices.”

At the same time, participants recognised that play required safety. Doctor 4 cautioned that “you can’t force it cause then people feel unsafe and then they don’t want to do it.” Students 1 and 2 also noted that taking time for creative enquiry could generate guilt, as it might be seen as secondary to more obviously productive work. Even so, participants’ accounts suggest that creative enquiry opened a provisional space in which experimentation, failure, and uncertainty became more bearable.

### Effects on clinical practice

Participants described three main effects of creative enquiry on how they experienced clinical practice: voice, release, and extending horizons. These should not be read as direct outcomes in a causal sense. Rather, they capture the kinds of effects participants themselves associated with creative enquiry.

#### Voice

For several participants, creative enquiry restored a sense of individuality and presence within clinical work. Doctor 4 said that watercolour allowed her to “take her veil off and feel human again.” Doctor 2 reflected that after 25 years of medical practice, creative enquiry had helped her realise: “There are two people in the consulting room. I’m as important as the patient in the room, fancy that Ivon.”

Some participants spoke of feeling valued again through creative practice. Student 3 said that “no one can make the same art as you […] you feel like an individual again.” Doctor 2 described feeling “heard and seen.” These accounts were especially striking when set against earlier descriptions of invisibility within medicine.

Participants also linked voice to authenticity in reflection and communication. Student 2 said that creative enquiry enabled “more authentic reflection as opposed to having to reflect on principles of good medical practice, which feels a bit stilted and it feels less human as well.” Doctor 4 similarly suggested that “by reflecting properly we can reconnect.” Doctor 5 extended this beyond self reflection, saying that creative enquiry helped him communicate with patients in a way that was “authentic and honest from your own point of view as well.”

#### Release

A smaller group, mainly clinicians, described creative enquiry as offering release from the strain of uncertainty. Here, the emphasis was less on understanding uncertainty than on obtaining temporary respite from it. Doctor 3 described ceramics as “stress relief” and “an escape and for [her] wellbeing,” adding, “I can’t imagine reading a guideline and feeling calm afterwards.” Doctor 4 similarly said that watercolour painting away from work allowed her to “do something that was calming” and helped her relax.

Students 3 and 5 added that the absence of fixed rules or parameters made creative enquiry feel “freeing,” with “no pressure.” In these accounts, creative enquiry did not eliminate uncertainty in practice. Rather, it provided a brief counter space in which participants could step outside its demands.

#### Extending horizons

Half of the participants described creative enquiry as widening perspective. Student 2 said it offered “different perspectives on something,” while Student 5 said it encouraged them to “think about the experience in a way that you’ve not thought about before.” Doctor 1 described this in terms of possibility: “What other ways could I look at this to generate more possibilities?” Earlier in the interview, she had drawn parallels between creative enquiry and design thinking, particularly the value of divergent thinking. Students 1 and 4 both described this as helping to “open your mind.”

Participants also linked this widening of perspective to patient care. Student 1 said that “the most beautiful thing about art is that everyone has completely different takeaways from it.” She connected this to medicine, explaining that people “will view a situation differently.” Student 3, a musician, described how writing songs from other people’s perspectives helped her “see things differently” and “step back” to see a bigger picture. Student 1 used the phrase “look at medicine in 3D,” adding that “you need to see someone in 3D” because attending to multiple dimensions of a person’s experience makes better care possible.

Across the dataset, participants described creative enquiry as uncertain, subjective, and improvisational. Within their accounts, these qualities supported engagement with uncertainty through emotional processing, experimentation, and broadened perspective, and were associated with a stronger sense of voice and moments of release.

## Creative enquiry: discussion

This study provides an exploratory account of how a small group of medical students and clinicians with prior experience of creative enquiry described its relationship to uncertainty in practice and training. Rather than treating uncertainty as a problem to be resolved, participants’ accounts suggest that creative enquiry creates conditions in which uncertainty can be more fully encountered, interpreted, and at times tolerated.

Three interrelated patterns were evident across the findings. First, participants located uncertainty within cultures of binary thinking and limited recognition of emotional experience. In these accounts, uncertainty was not only a function of incomplete knowledge but also a product of educational and organisational environments that privilege certainty, efficiency, and standardisation. Second, creative enquiry was described as an open ended process characterised by indeterminacy, emotional engagement, and play. Third, participants associated this process with changes in how they related to practice, including a renewed sense of voice, moments of emotional release, and a broader capacity for perspective taking.

Taken together, these findings suggest that creative enquiry does not simply help clinicians “manage” uncertainty. Instead, it appears to reconfigure how uncertainty is experienced. Participants described moving from a position in which uncertainty was constrained, suppressed, or problematised, towards one in which it could be engaged with more openly. In this sense, creative enquiry may function less as a tool for reducing uncertainty and more as a practice that makes it possible to remain with uncertainty without immediate closure.

In earlier iterations of this work, these patterns were summarised using the terms Personal, Protective, and Embracing. In light of conceptual concerns, we treat these not as a formal model but as a tentative heuristic that captures three orientations within participants’ accounts. “Personal” refers to the articulation of voice and the recognition of subjective experience within clinical work. “Protective” is better understood as temporary containment or holding of emotional intensity, rather than protection from uncertainty itself. “Embracing” reflects an orientation in which uncertainty is approached as an expected and sometimes productive aspect of practice. These orientations are not discrete stages or outcomes. They coexist and, at times, sit in tension with one another.

This interpretation aligns with theoretical accounts that position uncertainty as an inherent and generative feature of professional practice rather than a deficit to be eliminated. Participants’ descriptions of creative enquiry resonate with understandings of learning that emphasise disruption, perspective shift, and “thinking otherwise,” in which established ways of seeing are unsettled rather than reinforced. From this perspective, the value of creative enquiry lies not in offering certainty, but in expanding the range of ways in which uncertainty can be experienced and interpreted.

The design of the study places important limits on these claims. Participants were drawn from a small, self-selecting group already engaged with creative enquiry, and their accounts are likely shaped by this prior orientation. The findings therefore cannot be generalised to wider populations of students or clinicians, nor do they capture perspectives of those who may be sceptical of creative approaches. Rather than demonstrating effectiveness, the study offers insight into how creative enquiry is understood by those who use it.

For medical education, these findings suggest a shift in emphasis. If uncertainty is treated only as a problem to be managed, educational responses may focus narrowly on decision-making strategies and communication skills. By contrast, the accounts presented here indicate that engaging with uncertainty may also require space for reflection, emotional processing, and interpretive work. Creative enquiry may offer one such space. Its value lies not in resolving uncertainty, but in enabling learners and clinicians to engage with its complexity in ways that are cognitively, emotionally, and socially meaningful.

### Personal approach

Participants frequently described a sense of detachment within clinical environments, often accompanied by pressure to suppress or conceal emotional responses. Paradoxically, this appeared to intensify their experience of uncertainty. This observation resonates with Katz, who noted that physicians may become increasingly aware of their suppressed emotions, creating a cycle of emotional tension that complicates clinical judgement [55]. In this context, participants’ accounts suggest an ongoing tension between two forms of professionalism: one that accommodates emotional presence, and another that privileges detachment and control.

This aligns with Schattner’s argument that emotions are not peripheral but intrinsic to clinical practice [56]. Similarly, Doulougeri et al., in their analysis of 54 student narratives, found emotional suppression to be the most common regulatory strategy among medical students [57]. Such suppression may offer short-term functionality but risks limiting deeper engagement with uncertainty. In contrast, participants in this study described creative enquiry as offering time and space for emotional processing. This supports Ajjawi et al., who emphasised the importance of reflective space in enabling learners to work with emotions rather than bypass them [58]. Lawrence likewise argues that creative practices create conditions in which individuals can remain with emotion, rather than defaulting to purely rational modes of engagement [46].

A notable effect of this process was the restoration of voice. Participants described creative enquiry as enabling them to feel recognised and to express aspects of themselves that were otherwise marginalised within clinical settings. This finding is consistent with Brown and Younie, who suggest that creative enquiry facilitates emotional expression and enhances personal visibility [59]. The ability to articulate emotional experience may also have implications for clinical practice. Patel et al. note that unexpressed emotions in physicians can contribute to medical error and impaired judgement [47]. From this perspective, creative enquiry may support more reflective, attentive, and patient-centred care, not by eliminating uncertainty, but by enabling clinicians to engage with its emotional dimensions more fully.

At the same time, participants’ accounts also highlighted potential risks. One participant described how, when engaging with difficult material, emotions could “spill out” unexpectedly. This concern echoes SmithBattle’s work on arts-based learning, which notes that creative processes may lead to forms of self-disclosure that are not easily contained and may leave individuals vulnerable [60]. This underscores the importance of safe and supported learning environments when integrating creative enquiry into medical education.

Briefly, these findings suggest that creative enquiry does not simply provide an outlet for emotion. Rather, it may enable clinicians and students to acknowledge, articulate, and work with emotional experience as part of uncertainty, thereby extending professional practice beyond purely technical or procedural responses.

### Protective approach

Participants situated their experiences of uncertainty within a broader context of strain, with some describing the NHS as “broken.” This reflects wider concerns about systemic pressure and its impact on clinical work. Previous research has linked such environments to increased levels of uncertainty and physician burnout [32]. Participants in this study similarly described uncertainty as accompanied by anxiety and persistent concern, echoing findings from other clinical contexts [32, 61].

Within this setting, creative enquiry was often described as offering a form of relief. Participants emphasised its playful and improvisational qualities, which appeared to provide temporary distance from the demands of clinical practice. Importantly, “protective” here does not imply that creative enquiry removes or resolves uncertainty. Rather, it refers to moments of emotional containment or respite within ongoing uncertainty.

This interpretation aligns with van Gaalen et al., who found that medical students engaged in playful activities as a form of temporary escape from demanding routines, allowing them to experience uncertainty with less strain [62]. Similarly, Auerbach and Baruch describe participants in arts-based education as “players,” highlighting the role of play in reframing experience [42].

Participants’ accounts suggest that creative enquiry creates a bounded space in which the pressures of clinical uncertainty can be momentarily suspended. This space is not separate from practice, but offers a different mode of engagement. In this sense, creative enquiry may function as a form of temporary holding, allowing individuals to step back from uncertainty without denying its presence.

### Embracing approach

Participants consistently described clinical practice as shaped by binary expectations, often framed in terms of “right or wrong” or “black and white.” This reflects a well-documented tendency within medicine to simplify complex situations into discrete categories [24, 63]. While such approaches can support decision making, they may also obscure the ambiguity and uncertainty inherent in clinical work.

Scholars such as Simpkin and Schwartzstein, and Laughey and Finn, have criticised this tendency towards binary thinking, arguing that it can limit clinicians’ ability to engage with the complexity of real-world practice [33, 64]. Participants’ accounts echoed this concern, describing clinical reality as “murky” and resistant to simple categorisation.

This raises broader epistemological questions. As Thomas argues, the dominance of positivist approaches in medicine has encouraged a focus on measurable, observable phenomena, often at the expense of interpretive and relational dimensions of care [65, 66]. Participants’ descriptions suggest that this orientation may contribute to difficulties in engaging with uncertainty.

Against this background, creative enquiry appeared to support a different stance. Participants described becoming more willing to recognise, accept, and remain with uncertainty. This aligns with Domen’s argument that accepting uncertainty can enhance both clinician wellbeing and patient relationships [67]. Similar perspectives are found in the work of Malterud et al. and Engebretsen et al., who advocate for approaches that engage with, rather than reduce, uncertainty in clinical settings [68, 69].

At the same time, participants’ accounts reveal an apparent paradox. Creative enquiry is itself uncertain, characterised by open-ended and unpredictable processes. Yet repeated engagement with this uncertainty appeared to change how participants related to it. Several described this in terms of “exposure,” suggesting that familiarity with uncertainty in creative contexts may make it more tolerable in clinical settings.

This interpretation resonates with Beghetto’s notion of Dubito ergo creo—“I doubt, therefore I create”—which positions uncertainty as a generative condition for creativity [39]. Rather than eliminating uncertainty, creative enquiry may cultivate a different relationship to it, shifting from avoidance towards acceptance through repeated, voluntary engagement with uncertain processes.

## Limitations

This study has several limitations that should be considered in relation to Lincoln and Guba’s criteria for trustworthiness [70]. The use of convenience sampling introduced a clear source of bias, as participants were drawn from groups already engaged with or receptive to creative enquiry. Their accounts are therefore likely to reflect a favourable orientation towards such practices.

The sample was also relatively homogenous, particularly among student participants from a single institution, which may limit the diversity of perspectives and the transferability of findings. The absence of a comparison group further constrains interpretation. The study does not allow claims about whether creative enquiry is more effective than other approaches to engaging with uncertainty.

Although student participants had entered clinical training, they remained learners, and their perspectives differ from those of experienced clinicians. In addition, the study relied solely on interview data, without triangulation from other sources, which may limit the robustness of the analysis. Participants also varied in their prior engagement with creative enquiry, which may have shaped the depth and nature of their accounts.

These limitations are consistent with the exploratory nature of the study. The findings should therefore be understood as interpretive and situated, offering insight into how a specific group of participants described their experiences rather than providing generalisable conclusions.

## Conclusion

This study explored how medical students and clinicians with experience of creative enquiry described its relationship to uncertainty in practice and training. Participants identified recurring tensions within clinical environments, particularly binary modes of thinking and the limited visibility of emotional experience, which appeared to intensify uncertainty.

Creative enquiry introduced an alternative mode of engagement. Participants described processes characterised by uncertainty, emotional processing, and play, which were associated with a renewed sense of voice, moments of emotional containment, and expanded perspectives on clinical situations.

Taken together, these findings suggest that creative enquiry does not function as a technique for eliminating uncertainty. Rather, it appears to create conditions in which uncertainty can be more fully engaged. The previously described Personal, Protective, and Embracing orientations are best understood as a heuristic that captures these patterns, rather than as a fixed model.

For medical education, this points to the potential value of approaches that extend beyond technical and communicative strategies. Creative enquiry may offer a space in which learners and clinicians can engage with the cognitive, emotional, and interpretive dimensions of uncertainty. For policy documents such as the Medical Licensing Assessment (MLA) and the Royal College of General Practitioners (RCGP) handbook, this research may be a stepping stone to formally recognise creative enquiry as a valid and effective strategy for engaging with uncertainty, which could help standardise its implementation across medical training institutions.

Future research should examine how these practices are experienced by a broader and more diverse group of learners and clinicians, including those who may be sceptical of creative approaches. Further work is also needed to explore how such practices might be integrated into curricula in ways that are both supportive and contextually appropriate. 

## Supplementary Information

Supplementary Material 1.

Supplementary Material 2.

Supplementary Material 3.

Supplementary Material 4.

Supplementary Material 5.

Supplementary Material 6.

Supplementary Material 7.

Supplementary Material 8.

Supplementary Material 9.

Supplementary Material 10.

Supplementary Material 11.

Supplementary Material 12.

## Data Availability

All data supporting the findings of this study are available within the paper and its Supplementary Information. The study was conducted in accordance with the ethical standards of the institutional research committee and the principles of the Declaration of Helsinki. All participants provided informed consent to take part in the study and gave explicit consent for the publication of anonymised quotations and study findings.
