# Supplementary material for: How does creative enquiry enable us to deal with uncertainty?
Source: BMC Med Educ. 2026 Aug 8;26:1785. doi: 10.1186/s12909-026-10060-w (PMC13732821; doi:10.1186/s12909-026-10060-w)
Supplement: Supplementary file 1 [file 12909_2026_10060_MOESM10_ESM.docx]

**IK**
What does creativity mean to you as a medical student?

**P4**
I guess it’s exploring your own personal self. There’s also an element of sharing it and what you want to show in your creativity. You don't have to share everything, but I do think that there are benefits to sharing. You’ll be explaining what it means to you and talking to other people about what they think it means to them. And I think that is really big part of the creative process. Also if all this art was found one day, even if you didn't share it, after I'm alive it was found, what would people think? What would they take from it? And I think that's part of sharing.

Also you're exploring creativity by yourself and it's not being marked and it's not being submitted anywhere and it's just you improving on your own scale. That's part of the creative process as well.

As medical students lots of stuff is dependent on academics and grades and stuff. With creativity, it's not like you’re trying to fit into a box.

**IK**
Thank you. Could you talk about an example or several where you've used creativity to reflect on your past?

**P4**
I wrote a poem about burnout in medicine and social and cultural expectations. They were really personal to me. So in the poems I mentioned my Mum and my Dad and things like that. I think it is quite like therapeutic to like sit and evaluate certain experiences that have led to certain conclusions. So like maybe I would never have linked my own academics and my own feelings of perfectionism to family until you're really sad and thinking about how it all can make one picture.

So that was really interesting. And then also sharing it was really useful because I feel like a lot of people were able to see the intersectionality of everything. There's being a med student, but there's also being a woman and being a woman of colour. They're all different things that will lead to one feeling of just like being exhausted and everyone can feel exhausted. But maybe not everyone has all these individual factors. And I thought that was really interesting to explore.

Things that make people feel really lonely are actually stuff that everyone can feel or can relate to. Then it's really not that lonely anymore. And I think that’s a beautiful part of it.

**IK**
Yeah. Thank you. Have you done anything else related to creativity that's made you think differently on certain things?

**P4**
When I first started writing poems, it's when I was living alone for the first time. At the time, my friend had published a little short poetry book and she recommended to write some poetry.

And it helped quite a lot because sometimes I needed a lot of time to digest things and go through stuff a 100 times over. It’s a really good way of doing that in like a productive way. So it’s not just running through scenarios in your brain over and over. You're making it something creative and something beautiful. So by the end of all of that, you're not just sat with nothing and your own feelings. You can sit with something you can look at.

Before I was even in med school, I just started doing that just anytime things were really complicated and things were really hard. It’s an easy way to go through all the stages of emotions and then you can have poems that you can look back at weeks or years on after something has happened. It’s a really good example of your own personal growth and is tangible evidence that you've gotten better or you've grown. And I think that's helpful because you don't have to be so in your head.

**IK**
Could you describe a time in your life that made you feel uncertain?

**P4**
Applying to med school was a really uncertain time. I left home when I was 17 and that's when I was living alone. I took a gap year because loads of things changed all at once and I was not really sure. I was also a healthcare assistant. So I was dealing with lots of elderly patients and it was just after the COVID quarantine. So it was really uncertain because no one knew if COVID was going to come back or not.

I really wanted to do medicine my whole life but then thinking realistically whether it was going to happen. So I guess that's when I was journaling and writing poetry. It was a big collection of new things. When I left I was in year 13, so I was in school. But then school wasn't even the same because of COVID. And then I was a HCA and had this whole flat. I went from sharing a room with my sister to being alone. So I think there was a lot of things that I didn't really know about. I was probably a bit too young to be doing that. So it was like a lot of at one go. And I think that's what made that time really uncertain because it was all at one go.

When writing poetry, it didn't matter what I was doing. I could write one sentence and close a book. And that's the end of it. Compared to the other things at the time of living alone and applying for medical school writing poetry didn’t have pressure that everything else had.

**IK**
How would you typically approach uncertainty or navigate yourself around it when you come to an uncertain situation?

**P4**
I just really love talking to people about things. That's my biggest thing. I really enjoyed sharing stuff and I really loved it when my friend told me to do something because it was really easy for me to take her advice on board. I just think I'm somebody that likes surrounding myself around people and learn from them. I think I would say I'm quite an extrovert. I get my energy from other people as well. So no matter the situation, I can't imagine not calling somebody or not sitting with friends. I talk through things and all the options and all the outcomes and hope that people around me have the place to hear that. That's really important to me.

And that's kind of how I've always done it. And I will always probably do it is I will just find my people, walk it through with everybody and then sometimes it doesn't even have a solution at the end. But I think that's OK. I think that's part of it too.

**IK**
And now I'm just reading a statement to you. So in a scoping review, there have been multiple studies that have shown that creativity can enhance tolerance of uncertainty. So what do you views on this and why?

**P4**
Being creative is being uncertain as well. You are not sure what you're producing and you don't know what it will be like when it's finished. So I guess it's kind of an exposure therapy to uncertainty. You have to practise putting your feelings and thoughts to produce something or be creative. And then have to be OK with the fact that you don't know what it's going to be like and you won't be able to predict everything.

And that's like a really useful tool for approaching uncertainty. But also I think perspectives are really good. When you have to produce art from a situation, you have to force yourself to see it in like a different perspective.

I think people's brains go really fast and they think of all these things and it's really hard to navigate. So that's why it's a lot easier when you can express yourself to people or express yourself through art because you get to see stuff in front of you rather than have it all floating around in pieces.

**IK**
So why use something that is uncertain to deal with uncertainty?

**P4**
Sometimes there are no luxuries of certain things and you just really don't know. Nobody has an answer and there’s no amount of research. Sometimes things are just not certain. So then you can get stuck there and try and really look for answers and really look for an exact outcome. Or you can just embrace the challenge of it a bit. It's easier to digest when you can like do something productive, produce beautiful things and process your thoughts. It’s an optimistic way to look at the fact that you don't know everything.

**IK**
So do you have any examples of where you've had a creative approach help you engage or tolerate uncertainty in clinical practice?

Medical students are really trying to find exact scripts to follow or follow a flow chart of responses and treatments, but people won't fit into the boxes really nicely.

You have to adjust to what's in front of you, you have to still produce something good out of it, no matter what it is. You don't want people walking away disheartened or more upset than they need to be just because you're not willing to adjust your perspective. So I guess creativity plays a really big role in thinking on your feet and trying to make the best out of what you've got. It's the doctor-patient relationship; you want to work creatively with the patient. I know this is true because in OSCEs you could learn it all and have a whole script. But then something else happens and you just have to think on your feet. Creativity helps you consider a lot of things at once. It’s taking in all these thoughts, feelings and perspectives. So in a consultation, you have to take all this information and it might be new and confusing, but you still have to present it or talk to people. You have adjust yourself to each person and understand that they have different points of view and that’s what you learn from by integrating creativity.

**IK**
But now do you think it could ever be unhelpful?

**P4**
I don't think so. I mean, maybe I've not.

0:31:54.70 --> 0:32:24.70
**P4**
I’ve experimented enough for different things, but just not ever seen a scenario where I would think that creativity was the hindrance. I don't think I would ever turn to someone and be like, “Oh, I wish you were less creative”. Surely you'd want that from anyone? I just think people need stop seeing creativity as being really separate from scientific knowledge and facts. You have to be creative to make treatment plans. You have to be creative to fit your treatments into someone's life. Like, these are all things that integrate it.

**P4**
Creativity requires people being a bit more individualistic and it’s hard to get all these doctors saying the right thing if you have them be individual rather than giving them all the same script to work with. But even when we try and make doctors try and follow a script, they have to be creative anyway because a scenario will happen that won't follow the script.

**IK**
Is anything you'd like to add or anything you'd like to share that you thought?

**P4**
I feel like even if we know that it's beneficial and stuff, I don't think it's ever helpful to force creativity on people. I think that people will just get there when they get there and it will come in fun situations. And as long as people have the tools to access it, when they do find it, that's really great. But I don't think it is going to be something we can get a bunch of junior doctors to just start doing.
