# Supplementary material for: How does creative enquiry enable us to deal with uncertainty?
Source: BMC Med Educ. 2026 Aug 8;26:1785. doi: 10.1186/s12909-026-10060-w (PMC13732821; doi:10.1186/s12909-026-10060-w)
Supplement: Supplementary file 2 [file 12909_2026_10060_MOESM11_ESM.docx]

Appendix A – Promotional poster

Appendix B - Information Sheet

Participant Information Sheet

**Study title**

**How might creative enquiry enable you to deal with uncertainty?**

**Researcher’s name**

Ivon Kandiah, Medical Student ([ha23406@qmul.ac.uk](mailto:ha23406@qmul.ac.uk))

**Queen Mary Ethics of Research Committee reference number:**

**Invitation paragraph**

We would like to invite you to be part of this research project on creative enquiry in medical education if you would like to.

You should only agree to take part if you want to and is completely your choice. If you choose not to take part, there won’t be any disadvantages for you. Before you decide whether you wish to participate in this study, it is important for you to understand why the research is being done and what it will involve.

Please read the following information carefully before you decide to take part; this will tell you why the research is being done and what you will be asked to do if you take part. Please ask Ivon Kandiah ([ha23406@qmul.ac.uk](mailto:ha23406@qmul.ac.uk)) if there is anything that is not clear or if you would like more information.

If you decide to take part, you will be asked to sign the attached form to say that you agree.

You are still free to withdraw at any time and without giving a reason.

**What is the purpose of the study and what would taking part involve?**

This study aims to explore the medical student and general practitioner views on how creative enquiry enables you to deal with uncertainty.

If you are interested, we will invite you to come to an approximately 30-minute online interview. The interview schedules are designed to gather information related to your experience of exploring the creative enquiry, so the questions should not cause any distress. Further to this, you will be able to skip any interview questions or even stop the whole interview without giving an explanation, should you want to. The interview will be taped and written up as a document (transcript). After two weeks from interview, the data will be collated for the analysis.

Creative enquiry is still relatively new in medical education. We hope that this research will inform the understanding of potential value or issues with creative enquiry in the medical education curriculum particularly in relation to engaging with uncertainty.

**Why am I being invited?**

You are being invited to participate in this research study because you have completed the Creative Arts SSC in the last three years or are part of the Royal College of General Practitioners (RCGP) Creative Health Special Interest Group (SIG) or a clinician who is engaging with creative engagement in relation to clinical practice. You should not take part in this study if this does not apply to you.

**Do I have to take part?**

This participant information sheet has been written to help you decide if you would like to take part. It is up to you whether you wish to take part. If you do decide to take part you will be free to withdraw at any time without needing to provide a reason, and with no penalties or detrimental effects.

**What are the possible benefits of taking part?**

Exploring interviewees’ lived experiences and with opportunity for reflection in a safe place may be beneficial as well as support further understanding of creative enquiry, a new and growing field.

**What are the possible disadvantages and risks of taking part?**

This study is not designed to cause any potential distress to participants.

In the unlikely event that you feel uncomfortable in taking part in the interview, we will help you consider where best to turn for support. Ivon Kandiah will signpost interviewees before, during or after the interview as necessary, for example, towards student support or conversations with the research supervisor LY who is a GP academic.

**Expenses and payments**

**N/A**

**What information about me will you be collecting?**

We will collect the transcript data from the interview. This is to help us analyse patterns brought up between several participants.

**Where do I get more information?**

Ivon Kandiah is the lead researcher for this research project conducted within his Intercalated BSc in Medical Education. Professor Louise Younie is the educational supervisor for this study.

If you have any queries or would like more information, please contact Ivon Kandiah at ha23406@qmul.ac.uk

**How will my data be stored and who will have access to it?**

Your data will be stored in fully anonymised format on password protected computers, and only the researchers will be able to access it.

**When and how will my data be destroyed?**

The only individuals who will have access to the data will be the researchers and all transfer of data will be password protected complying with Queen Mary policy.

In line with the Data Protection Act (1998), all data will be stored on MS teams and destroyed after 2 years.

**How will my data be used and shared?**

The results will be written up for a dissertation report in an anonymised format. The data may be presented at academic conferences or written up for publication.

**Under what legal basis are you collecting this information?**

- Queen Mary University of London **processes personal data** for research purposes in accordance with the lawful basis of ‘public task’.
- Please read [Queen Mary’s privacy notice for research participants](http://www.arcs.qmul.ac.uk/media/arcs/policyzone/Privacy-Notice-for-Research-Participants.pdf) containing **important information about your personal data and your rights** in this respect.
- **If you have any questions** relating to data protection, please contact Queen Mary’s Data Protection Officer, Queens’ Building, Mile End Road, London, E1 4NS or [data-protection@qmul.ac.uk](mailto:data-protection@qmul.ac.uk) or 020 7882 7596.

**What will happen if I want to withdraw from this study?**

You are free to withdraw from the study at any point and/or to withdraw from the study without any explanation, up to the point of thematic analysis (up to 2 weeks after the interview).

**What should I do if I have concerns about this study?**

- **If you have any concerns** about the manner in which the study was conducted, in the first instance, please **contact the researcher(s)** responsible for the study Prof. Louise Younie.
- **If you have a complaint** which you feel you cannot discuss with the researchers, then you should **contact the Research Ethics Facilitators** by e-mail: [research-ethics@qmul.ac.uk](mailto:research-ethics@qmul.ac.uk).
- When contacting the Research Ethics Facilitators, **please provide details** of the study title, description the study and QMERC reference number (where possible), the researcher(s) involved, and details of the complaint you wish to make.

**Who can I contact if I have any questions about this study?**

**Supervisor for student project	Student Investigator**

Professor Louise Younie	Ivon Kandiah

[m.l.a.younie@qmul.ac.uk](mailto:m.l.a.younie@qmul.ac.uk) [ha23406@qmul.ac.uk](mailto:ha23406@qmul.ac.uk)

Appendix C – Consent Form

Consent Form

**Title of Research Study:** How does creative enquiry enable you to deal with uncertainty?

**Principal Investigator:**

Ivon Kandiah, Medical Student ([ha23406@qmul.ac.uk](mailto:ha23406@qmul.ac.uk))

**Queen Mary Ethics of Research Committee Ref:**

Thank you for your interest in this research.

Should you wish to participate in the study, please consider the following statements. Before signing the consent form, you should initial all or any of the statements that you agree with. Your signature confirms that you are willing to participate in this research, however you are reminded that you are free to withdraw your participation at any time.

| **Statement** | **Please initial box** |
| --- | --- |
| 1. I confirm that I have read the Participant Information Sheet dated 23.11.23 version 0.1 for the above study; or it has been read to me.  I have had the opportunity to consider the information, ask questions and have had these answered satisfactorily. |  |
| 2. I understand that my participation is voluntary and that I am free to stop taking part in the study at any time without giving any reason and without my rights being affected. |  |
| 3. I understand that my data will be accessed only by the research team. |  |
| 4. I understand that my data will be securely stored on MS Teams and in accordance with the data protection guidelines of the Queen Mary University of London until 16.05.25 in fully anonymised form. |  |
| 5. I understand that I can access the information I have provided and request destruction of that information at any time prior to thematic analysis, up to 2 weeks after the interview. I understand that following this time I will not be able to request withdrawal of the information I have provided. |  |
| 6. I agree to the interview being audio and video recorded. |  |
| 7. I understand that the researcher will not identify me in any publications and other study outputs using personal information obtained from this study. |  |
| 8. I agree to take part in the above study. |  |

Participants should read [**Queen Mary’s privacy notice**](http://www.arcs.qmul.ac.uk/media/arcs/policyzone/Privacy-Notice-for-Research-Participants.pdf) **for research participants** which contains **important information about your personal data and your rights** in this respect.

If you have any **questions relating to data protection**, please contact Data Protection Officer, Queens’ Building, Mile End Road, London, E1 4NS or [data-protection@qmul.ac.uk](mailto:data-protection@qmul.ac.uk) or 020 7882 7596.

________________	_____________	_____________________________

Participant name	Date	Signature

__________________	_______________	________________________________

Name of person	Date	Signature

taking consent

I confirm that I have carefully explained the nature, demands and any foreseeable risks (where applicable) of the proposed research to the participant and provided a copy of this form.

**Supervisor for student project	Student Investigator**

Professor Louise Younie	Ivon Kandiah

[m.l.a.younie@qmul.ac.uk](mailto:m.l.a.younie@qmul.ac.uk) [ha23406@qmul.ac.uk](mailto:ha23406@qmul.ac.uk)

Appendix D – Interview Schedule

**Interview Schedule: Exploring Creative Enquiry and Tolerating Uncertainty**

Introduction and Warm-Up

1. **Understanding Creative enquiry:** What does creativity mean to you as a medical student/ clinician?

Reason: To establish a common understanding of the term before delving into its application.

2. **Using Creative enquiry:** Could you talk about an example where you have used creativity in the past to reflect on clinical practice during or after?

Reason: To elicit a real-life scenario for detailed exploration.

3. **Encountering Uncertainty**: Can you describe a specific instance where you faced considerable uncertainty in your clinical practice?

Reason: To elicit a real-life scenario for detailed exploration.

4. **Approaches to Handling Uncertainty**: How do you typically approach or navigate uncertainties in clinical settings?

Reason: Understanding their existing strategies or frameworks for managing uncertainties.

5. **Views on literature:** In a scoping review, there have been multiple studies that have shown that creativity can enhance tolerance of uncertainty. What are your views on this and why?

Reason: To show that creativity does enhance tolerance of uncertainty and then hear their views on it.

6. **Examples of Creative Solutions:** Do you have any examples where a creative approach helped you in engaging with or tolerating uncertainty?

Reason: Illustrating the practical application of creative enquiry.

7. **Benefits of Creative Enquiry**: How might creative approaches be helpful in clinical practice?

Reason: Understanding the perceived advantages and outcomes of employing creative strategies.

8. **Challenges Faced**: How might creative approaches be unhelpful in clinical practice?

Reason: Highlighting potential hurdles or limitations in using creative thinking for uncertainty.

9. **Closing Thoughts**: Is there anything else you'd like to add or share regarding the role of creativity in tolerating uncertainty in clinical practice?

This is general interview schedule which may be amended.

1) Based on interviewee responses
2) Iteratively throughout the data collection phase of the project

Appendix E – Ethics Approval Form
