# Supplementary material for: How does creative enquiry enable us to deal with uncertainty?
Source: BMC Med Educ. 2026 Aug 8;26:1785. doi: 10.1186/s12909-026-10060-w (PMC13732821; doi:10.1186/s12909-026-10060-w)
Supplement: Supplementary file 4 [file 12909_2026_10060_MOESM1_ESM.docx]

**IK**
What does creativity mean to you as a clinician?

**P1**
Oh, that's a really good question. Creativity generally means creative pursuits to me outside of medicine. So dance, singing, art, photography, cartooning, improvisation, all of those things. Within clinical medicine it relates to the one to one interaction with patients being creative in our communication. It relates to being creative in working with colleagues. It also relates to innovation and entrepreneurship. I’m an NHS clinical entrepreneur and I think that quality improvement and innovation is really important not just for improving healthcare, but for our well-being as well. So lots of different facets of it.

**IK**
Thank you and you were saying about quality improvement. So this idea of creativity is key to that. So could you tell me more about how creativity helps?

**P1**
Absolutely. So I think it's an attitude really. So looking at what we do in a critical way and thinking how we could improve it. So how we could make it more inclusive for all patients or how we could make it more efficient, time and energy saving. All sorts of aspects of innovation and quality improvement. that it's quite broad.

**IK**
I’m sure you’ve used creativity in the past and we both seen each other in the creative SIG workshop. But I'd just like to broaden it out to whether you've done any creativity in the past to help you reflect on clinical practice? And just your views on that. How you did that? How you felt? That will be great.

**P1**
Well we could be here for a long time, I think. I've really enjoyed experimenting with different forms of creativity. My masters in medical humanities in 2013 to 15, I discovered graphic medicine as well as medicine on film, narrative medicine, all of all of those aspects of medical humanities. From that point I started doing cartooning graphic medicine for reflective practise. So that's quite a long time now since 2015 and then facilitating that for other doctors and trainees and dentists and people as well. I also use cartooning with other coaches because I'm a coach and I supervise other coaches coaching doctors in different organisations: the faculty of medical coaches, the coaching supervision academy and NHS London leadership academy. So lots of contact with the professional support and Health Education England. So I've facilitated creative workshops for them as well using cartooning. I've done it internationally in America, Istanbul, Paris, with doctors from different specialties and across the UK. So that's using it personally creativity for my own reflective practise but supporting that in other people as well and also for people that are supporting doctors. So coaches but also medical educators to different levels. For example, couple of weeks ago I was down in Dorset facilitating with lead appraisers who support appraisers who appraise GPs. I was doing a creative workshop with them to get them to think about how they can bring creativity into the work that they do with GPs to get their GPs to think about their own creativity for their well-being. So it's kind of multi-level really. And I'm very fortunate to have a lovely team of collaborators. Not just cartoonists, but also a jazz singer, comedians, improvisers, artists, poets, authors, magicians. Lots of different people that I collaborate with doing different types of creative reflection.

**IK**
Thank you. I think you're describing the idea that you've seen creativity at a multi-level, so in different stages of their development and training. You've also seen it in multiple different types. So I think you were saying narrative medicine, cartooning, and then now you're saying about photography and artists. So it'd be great to hear a bit more about the cartooning because I'm sure that's kind of something you've enjoyed. And how is that for you? And was that any special to the other different types of reflection?

**P1**
As a coach to doctors, I work with a lot of doctors that are burnt out or considering leaving the profession or having career dilemmas. So I'm very much aware that those people are at a point where they need support because things have gone the wrong direction for them. And I think we need to do more kind of upstream to support people with their well-being. So the cartooning workshops are one way of doing that because we get people to reflect on positive things that are going well, ‘sparkling moments’ in their work. In healthcare, we tend to always reflect on challenges, significant events, complaints, things that have gone badly or could have gone better. And that's very much true of the portfolios for trainees and medical students, but also appraisal for people that are practising. The structures demand that we reflect on negatives. So inviting people to reflect on what's going well, it's an organisational development tool called appreciative inquiry, which can transform cultures by improving attention to positive emotions and optimism and hope, rather than subscribing to this overwhelming negative discourse about the NHS being broken, failing and under-resourced and all of that. It's really quite unusual to offer people opportunities to reflect on positives. And the reason that's important is because burnout, which is a social construct, but Maslac defines it as low energy, seeing patients as all the same and the feeling you're on a treadmill and it's monotonous. The third aspect is low self efficacy. So the feeling that you're not doing a particularly good job Anybody could do what I do. What the workshops do is create the opposite of that so they're energising. They make people reflect on individual unique encounters with patients. And what went well and why and they make them think about it in a very different creative way. Breaking it down into a cartoon makes you think about what happened? Who said what? What was their facial expression like? What were they thinking in a thought bubble? It makes you really think about it in a lot of detail. So that tackles the second aspect of burnout, which is when you see everybody as the same. It makes people think about the unique particular of each encounter. And the third aspect is self efficacy. So it makes you think, “Oh that went really well. I am doing a good job”, which is really good for you. So the opposite burnout is engagement. Essentially this creative process coupled with appreciative inquiry and getting feedback from peers on what you've drawn in your cartoon, increases engagement and well-being, so that’s why I like doing it.

**IK**
If you could describe a time in your life where you faced uncertainty?

**P1**
During the midst of the pandemic in the middle of the lockdowns, I was working as a COVID-19 GP on the COVID-19 line, and that was a real time of uncertainty because people didn't even have oxygen saturation monitors in the early days. So we were having to make decisions about whether to send an ambulance to people and admit them to hospital or whether for them to stay at home and kind of wait out their symptoms in the hope that they wouldn't get worse.

So that was that was really difficult as a clinician sitting in my home on my own at my dining room table, talking to people in Yorkshire or Lancashire or Devon about their breathlessness and trying to make a clinical judgement about whether they were somebody that was going to need intensive care imminently or not. That was the most challenging clinical uncertainty that I've faced. But there were also uncertainties in that there were lots of safeguarding issues, domestic violence escalated and mental health crises. So a lot of the people that I was talking to were having all these other things going on in their lives that came up incidentally during those conversations.

At the same time being mindful of resources, we couldn't send ambulances to everybody. That was probably the most uncertainty that I've faced in my career.

**IK**

So what made the decision that you were taking during COVID more uncertain?

**P1**
At the moment there's a TV series called ‘Breathtaking by Rachel’. She was a journalist author who became a doctor and has written books since. But it's about that that time that we faced in COVID from the perspective of secondary care. She was a secondary care doctor. But it's really brought this back to me.

We didn't know that some people that seemed objectively not to be that unwell, they were mildly breathless. When you checked their sats, they might be in the 80s, so they were really ill. But just talking to them remotely on the phone we didn't know that. And at the beginning we didn't realise that was happening. So looking back it was really challenging to try and assess those people.

**IK**
How would you try and deal with it?

**P1**
A diagnostic reasoning is very much ingrained. I've been a doctor for 32 years and so I have kind of innate structures that you work through in trying to assess somebody clinically, whether it's remote or in the person in front of you. So I think that those are really important and that's how you manage the uncertainty. You've got a structure that you work through.

There's also a strategy called “Stop and think”, which we used to teach for Imperial. I used to teach clinical diagnostic uncertainty with final year students, and that's asking yourself questions like, “What's the worst case scenario here? And what haven't I thought of? I'm thinking about this anatomically and so how else do I need to think about it? What other ways should I be thinking about this?” So it's kind of interrogating your thinking on a meta level. Having those mechanisms that you use that's a mode of reassuring yourself about uncertainty and also you draw on a vast history of cases that you've seen, particularly cautionary tales. I think that's something that we use a lot in medicine. So for example, when I was a GP trainee, one of the salaried doctors in the practice I worked in Camberwell said to me, “Whenever you see a little boy with abdominal pain, check the testicles, because if you don't it will be torsion”. And that was because she'd had a terrible experience where she missed torsion. So we use cases, anecdotes and cautionary tales, which really stick in your mind.

Whether it's something that you've encountered yourself or it's something that other people have told you about or you've read it in the MDU cautionary tale.

So having structures, thinking in pre-programmed ways, thinking critically and analytically about your thinking, but also having in mind cautionary tales are a range of mechanisms for coping with uncertainty.

This person's breathless. It could be pneumonia, asthma, all the respiratory. But what else? Well, it could be renal failure or it could be a PE. You know, it's not necessarily respiratory. So interrogating your thinking to think, what have I not thought about? I'm thinking about the obvious. But what other anatomical systems could be involved? It could be the kidneys. Nothing to do with the lungs. Or it could be a panic attack. It could be psychological. And probably the worst case scenario is a PE that you've not considered.

I guess the other side of us managing uncertainty is having a belief in your patient not being a total idiot. You have to safety net and you have to trust them.

**IK**
In a scoping review, there have been multiple studies that have shown that creativity can enhance tolerance of uncertainty? So what are your views on this and why?

**P1**

That’s a really interesting question. It's a really good question. The direction that my thinking has just gone in is design thinking as another angle on what creativity is. The principles of design thinking are not to be attached to one solution, but to just generate lots and lots of ideas about different solutions. And what designers do is they create lots of different prototypes for their thinking, but they throw them away very easily. They're not rigidly attached to them. So I think there is that element of creative thinking that is divergent. So you open it up to what are all the possibilities, including the possibilities I haven't thought of, and what other ways could I look at this to generate more possibilities? You're not kind of rigidly attached to this one prototype idea.

So that's a really interesting aspect of creativity which overlaps with coaching thinking.

As a coach, we get people who are stuck with an issue to look at it from more different perspectives, and to think about more different possibilities of how they might tackle this issue. So that's divergent thinking. And then the second bit of the creative or design process is to narrow it down and discard those prototypes that aren't going to work for whatever reason. And then narrow it down to what you think's most likely or the most important for what you're going to do. So that's my kind of understanding of creative thinking.

So I suppose in in diagnostic reasoning, your criteria are the probability, the symptom to the symptoms and signs fit.

0:25:39.450 --> 0:25:45.250
**P1**
Aren't they? You know, those are the criteria that you use to narrow it down. Really that? Yeah.

**IK**
Does creativity have different parameters?

**P1**
Yeah, I suppose with the clinical reasoning, divergence are the questions like, “What haven't I thought of for this certain scenario? I'm thinking about it anatomically. How else could I think about it?” And then the convergence is eliminating it according to criteria that you've chosen.

**IK**
Could you explore examples where you've used a creative approach in engaging with tolerating uncertainty?

**P1**
Yeah, that's really interesting. I'm thinking about what we were talking about earlier, you know, remote work during the pandemic. And how we managed to carry on, although it was uncertain if we were doing the right thing or if we were admitting the right people to hospital.

So what aspects of creativity did I use to help me live with that uncertainty?

I used my peers so conversations with colleagues that were also sitting at their dining room tables and were doing the same decision-making. I was benchmarking what I was doing with what other people were doing and that helped to manage the uncertainty that we were working in a really challenging situation, but we were all doing our best. We are using the same kind of thinking and processes to make these really difficult decisions. So that was one thing. Well, they are creative conversations between colleagues.

I did also reflect using cartooning. And I was meeting with the Graphic Medicine Community every Sunday, most of them in America. We would cartoon together online.

And I remember one of the cartoons was quite humorous because it was a funny situation. I was talking to a patient and she didn't sound too bad. She was walking up and down stairs without feeling too breathless and those were the sort of questions we were using to try and decide whether people needed admission or not.

So I said to her, the way you're describing your breathing doesn't sound too bad, but it sounds really noisy. And she said to me, ”Oh, no, that's not me. It's the dog. Get down, Rover”.

So I guess I do use humour. That was a very funny situation in the midst of tragedy and real uncertainty and difficulty. But there were moments that were quite funny. Capturing that in a cartoon and using dark humour in medicine, is way to try and live with uncertainty. These are really difficult times, especially if they've got the bloody dog on the bed. (chuckles) It’s about looking for sparkling moments.

And there were other sparking moments as well. So there was a teenager who had been in an awful situation with her parents. Breaking up in the middle of the lockdown, domestic violence going on and this poor teenager was stuck in the house with all that going on. She was safe because her father had moved out. But she was very traumatised by all of this.

And so I asked her a coaching question, “What has helped you with this stressful time?” She said, ”I also do cartooning”. She said that she drew manga and that was her release of stress. It was such a strange coincidence. And the creative means of cartooning allowed us to connect at a different level. I've cartooned that cartoon of her cartooning and her telling me about her cartoon. (chuckles) It's multi-level and a really lovely example of parallel process. I think that the arts can sustain our patients as well as us.

The interesting thing happened six months later. Well, all of our calls were recorded. But somebody that I knew through the Royal College of GPs was auditing my calls to check my management of patients and he gave me written feedback on my calls. He happened to have listened to that one, and he'd been in one of my cartooning workshops, which was again a strange coincidence. He gave me lovely feedback on how I'd established rapport with her. And how we'd identified her coping strategies.

**IK**
Looking at creative approaches, how can it be helpful in clinical practice?

**P1**
I've thought about this a lot because it is the subject of my PhD. I think it works in all sorts of different ways. Going back to what we were saying about burnout, when doctors do creative work together and give appreciative feedback, they're seen. Often the doctors are invisible in care. The focus is on the patient, the doctors are invisible. So allowing people to share their sparkling moments and get authentic, appreciative feedback from colleagues is a way that they feel seen and valued.

So that's one way. I think a lot of the creative practises are embodied. The actual kind of embodied nature of improvisation or singing or drawing or creative writing. They're all very much embodied, and we live in our minds as doctors, so there's something really cathartic about doing something embodied.

**IK**
So what do you mean by embodied?

**P1**
So moving your body as in vocal warmups, singing, physically moving or the movement of writing or drawing or sculpting. That's another way that is good for us.

It's playful. It's permission to not think logically, so that's what I love about improvisation.

You have to think illogically, to do improvisation. It's playful and the thing that really taps into is self-criticism and imposter phenomenon. If you subscribe to that again, it's a social construct. With improvisation, you have to be not good enough to be an improviser. It’s kind the opposite of perfectionism. It's being willing to fail and be stupid. That's quite refreshing and creativity allows you to do that and you don't have to be perfect. Your cartoon can be a stick figure. It doesn't have to be a work of art. It doesn't have to be perfect. It's totally a process for you. It's not about the product. It’s permission to fail.

A joint endeavour that's creative. There's something really special in that I think.

**IK**
Thank you. How might creative approaches be unhelpful?

**P1**
I think generally it is helpful because it's useful for tailoring what you're doing to that individual unique situation. You're addressing the particularity of that individual patient in front of you. It's where the creativity is helpful.

**IK**
Is there anything else you'd like to add?

**P1**
A lot of my coaching of doctors and trainees is when they're at transition points.

**P1**
I hadn't actually thought that angle on it till you asked.
