# Supplementary material for: How does creative enquiry enable us to deal with uncertainty?
Source: BMC Med Educ. 2026 Aug 8;26:1785. doi: 10.1186/s12909-026-10060-w (PMC13732821; doi:10.1186/s12909-026-10060-w)
Supplement: Supplementary file 5 [file 12909_2026_10060_MOESM2_ESM.docx]

**IK**
What does creativity mean to you as a clinician?

**P2-Dr**
I mean, you've got creativity in your job, so there's creativity in a way being a GP. So being a GP, there's a lots of particularly deprived area. You can be creative in some of your plans that you make with the patients. Now we're involving social prescribers and some of that can be creative.

You can give creative things to patients; so you can talk about art and poetry and if we had arts and prescription, I'll be referring them to concerts and choirs and things like that. So you've got, what you do with the patient or for the patients. And then you've got what you do for yourself. I've come to this quite late so I think I only have discovered kind of writing and poetry in the last few years. People like you are being creative from being a medical student and that's the way to go. No one in any shape or form told me about creativity in my medical curriculum a long time ago. So for me the main thing around creativity is that it's really helped me to deal with some of the stuff that I hear from patients. The not good stuff. In other specialties they have reflection and they have psychologists and they have supervision. In primary care, you don't have any of that. So you have to deal with it on your own.

**IK**
Yes. So the creativity is kind of having to deal with different situations as a clinician or as a GP and using your expertise in different ways.

**P2-Dr**
Yeah. I mean, it's a space to actually somewhere to put your feelings into. So you couldn't put yours into your music. I could put mine on to the page. Other people can put theirs on to the page, like artists drawing. It's almost like a container to put all your stuff in. And a container then to take out all you know. It's almost like there's a container that you put in. Maybe it's like an ocean that you put all your kind of stuff in, but actually out of the ocean, then comes beautiful waves of goodness. Maybe it's also like the divine. Maybe we're talking about the divine, Ivon. So, you've really got me onto a ramble now.

**IK**
Yeah. And what do you mean by divine, if that's OK?

**P2-Dr**
I use that for God. I'm a Christian, and so I sometimes say the divine 'cause people identify with that more than when you say God.

**IK**
If it's helpful by me saying that I'm a Christian as well, then I can understand by you saying God. So if you do want to use it during the interview, then I would understand what you're saying.

**P2-Dr**
OK.

**IK**
And you also said that this is something you've come on to very recently. So looking back, reflecting on your past, do you think that you were creative but just in a different way that you didn't recognise that as creativity.

**P2-Dr**
Possibly, but I actually think I poured more of myself into my kind of work roles and it was almost about saving the world. As a Christian, Jesus says give the shirt off your back. I thought he meant it literally. So whether you're running around at church, running around in your job, working with the homeless, working in a prison, all of this, as well as trying to raise a family, it's actually quite stressful. And I think there was something about not allowing myself, because it's taken me a long time to realise that there are two people in the consulting room. Everyone tells you whether it's the NHS, and even churches that the ‘other’ is the most important. It has taken me long time to realise that I'm as important as the patient in the room, fancy that Ivon. Imagine that. Imagine that you are as important as the patient. Everyone talks about the patient and even now in my practice it is all about the patients and I know because I've got this secret little space now and the sequel kind of, I know actually it's not all about the patients. There's two people in the room.

**IK**
Thank you. And now moving on from that to talk about your own experiences of creativity. So could you talk about an example where you've used creativity in the past to reflect on clinical practice?

**P2-Dr**
Well, I've written some poems and things and some of it is around the frustration around working in a deprived area. I don't know why my patients are biologically 15 years older than they really are. Poetry is illumination. Poetry is activism. Poetry can be used in all kinds of forms. Yeah, so I've got a few pieces published in British Journal General Practice Life. One got picked up then and was put into the BJGP the proper magazine, which was the one around why life is so unequal. But when you think most people reading that are from Middle England, it did talk about what it feels like and means to work in a very deprived community. So I'd like to do a bit more like that, but it's just trying to find the spaces to do that.

**IK**
And when you mean space does that mean time?

**P2-Dr**
No, it’s more about what do I do with it? So if I've written stuff, where do I get it published? Should probably self publish a book or try and get it. You know, where do you go with it? And I guess that's the thing about art. How much are you doing it for yourself? I kind of want to get it out there. But where do you go? How do you get out there? The admin around writing or getting your art out there or whatever could actually be quite time consuming and it's how much time. How much time do you choose to give to it? How much time do you choose to give to your proper work? What is your proper work?

**IK**
And you said, correct me if I'm not summarising right, but this idea that it’s important to share the work that you do. So it's important to use it to reflect on yourself, but at the same time share that in different mediums or ways.

**P2-Dr**
Yeah. So if it's around kind of inequalities, share it. We hear the data, but we don't hear the feelings or the qualitative stuff. It's almost like NHS England almost want to take the feelings out of medicine, so we become like robots. So in fact that is what the NHS England want; they want access at all costs. So it becomes transactional. That's the easy medicine. But what you do with all the other stuff.

**IK**
Yes, thank you. So, now looking at an experience of uncertainty. I'm sure you must have had loads of experiences of uncertainty in clinical practice, but could you describe, if possible a time that you remember very clearly as soon as I tell you the question?

**P2-Dr**
Well, yeah, there's lots. I mean it's interesting, 'cause actually being a sort of older GP you sort of know where to go so your uncertainty becomes less the more we with experience. You know you've got a set of people to ask or you know you're referring to secondary care and if you go through a few departments actually then you know it's a huge element of psychosocial impacting. So yes, there's lots of medically unexplained kind of symptoms and that people complain about although I think you can always put it then in the box of psychological overlay. I haven't seen it for a while, but I have got one patient who says it began very suddenly after she had a hysterectomy. So she had a really bad sore throat, kind of reflux. She was losing weight. She couldn't eat, but she went through gastro. She went through ENT. She went back to the surgeons. They looked at the anaesthetic notes to see whether it was from something in the operation. And this is now 15 months later what she's been through. Then she was at the Homerton, then she wanted to go to all the departments of UCLH. She’s had CT scans and barium swallows and every sort of thing. And still there's no real improvement. Yeah, that was a bit of a sort of strange case and quite frustrating at times. Actually, she stopped seeing me now, so I don't know what she's doing.

**IK**
Yeah. So the idea that uncertainty can get better with time and with experience. But does it come once in a while?

**P2-Dr**

Of course it comes. But I guess you've got more places where you know where to discuss the case. So it doesn't necessarily feel scary or fearful. Sometimes it's bit like a puzzle that's quite interesting. Sometimes it's just boring because it involves lots of referrals. I think I'm quite careful of how much time I spend on each case now, because I kind of want to do other things. In fact, I only do 2 sessions now. I was doing 3 and now going down to 2 sessions in the in the practice. I'm also a clinical director for our primary care networks, so I do that as well. So I do other things and I don't want to then spend too much time on one bit of what I do, if you know what I mean.

**IK**
Yes. And just now you mentioned you’re a clinical director, so that must be a different role. But in that role, have you seen uncertainty differently? So it might not be with patients, but with management or how you have to deal with roles?

**P2-Dr**
Yeah, I mean again, I've been doing it now for five years, but it was very uncertain that PCNs came out of nowhere and it wasn't certain how it was going to all kind of work with the CCGs and the system. The system really didn't like us. That's quite a bit of opposition really. So yes, where we were we very quickly because of the 8 net primary care networks in Hackney. So we very quickly we went through forming, storming, norming. We formed a team of clinical directors and actually that was very supportive. I think I'm very strong on. I've done my kind of my values. I'm very relational and kind of sociable, sort of extrovert relational. And that's what is my primary motivation. So in fact, where I work, then you very quickly find your kind of team. That always kind of helps me. I always say you can do anything when you're part of a team or you have a team behind you.

**IK**
Thank you. And now moving on to the idea of navigating uncertainties. So you could talk about in clinical settings, but also just generally in life. Anything in your past experience. So how do you typically approach when you encounter uncertainty?

**P2-Dr**
Well, I have done some quite big things so we moved to South Africa. We then moved back. We we're there for 4 1/2 years. Getting married. It's quite kind of uncertain. You don't really know what's going to happen. Having kids. Again you don't really know. All of those are uncertain. And to a certain degree, life is uncertain. Everyday, isn't it? You know, we take our lives for granted. Both of us now got elderly parents. The two that are left are quite well, but they're going to slowly deteriorate now that they're 85 and 86. So there's a lot of uncertainty ahead - what the kids do. But in a way, I think having a faith makes you know. Everything's uncertain, but then actually everything is certain with God. So that's my rock. God is my rock. Although being a Christian doesn't exempt you from any uncertainty or any tragedy and any hardship, what it does do is that God is with you so.

**IK**
Yes, and just asking you a question now, different people that I've interviewed and some of them have described their faith and it might be in different religions as well. A key thing I think people say is this idea of putting your burdens on something else or someone else. So do you think there's any overlap really between the religious or God in the sense of “OK, I trust in something” and allows you to put your worries on something, does the creativity have some sort of overlap in the sense of putting your emotions down on a piece of paper when you write your poetry? Or are they both very distinct?

**P2-Dr**
No, no, I mean I think it is. I haven't got into writing prayer poems yet, but I think that is probably coming. I think that I'm too conscious of the secular world if I'm writing. So don't write kind of that sort of. But I read some of them. But yeah, absolutely. It goes back to the first thing I said about this kind of container. You know, you want to put your emotions in there and actually that ocean is God. And out of that comes the goodness of God and the faithfulness of God. I know what you mean. Sometimes it's also about walking my mind. My biggest picture of God is walking the Emmaus Rd. So it's a Jesus, a God who walks with us. So although my path, it might be like this (using hands to show a winding path) in fact I probably stray off the path most of the time the narrow path actually, even though you're straying God is still with you in the in the peaks and the valleys or whatever. So in fact I would navigate uncertainty with a God who is with me, that's slightly different. But how does that play out in creativity then? Don’t know.

**IK**
There’s also a question that I want to know for which the answer I'm not sure. I think both are of course distinct and they have certain qualities, but like you are describing now, some of them might be the same. So the idea of kind of giving you a direction or making you understand. But of course, certainly there are some things that are different, but it's very inspiring to hear from a different person and somebody who is much more experienced than me. Their take on it, especially like in clinical practice and in life. Thank you. And of course you can come back to this or we can share if you think that is helpful for other questions to answer. So I'm just reading out a statement now. In a scoping review, there have been multiple studies that have shown that creativity can enhance tolerance of uncertainty. So what are your views on this and why?

**P2-Dr**
I guess the starting premise is that dealing with uncertainty is very stressful. Constant uncertainty. So isn't there something around the more grounded you are as a clinician, the more unstressed you are, then the more you will tolerate. I guess if your own creativity, self-care, the arts help you to be grounded, then that's going to enhance your tolerance of uncertainty. In medicine you can run around like a headless chicken achieving some things or you can walk a bit slower and think and be more grounded and probably achieve more. That has taken me about 25 years to realise that. So I'm telling you now, Ivon. So you will know this at the age of 21. So the more grounded you are as a clinician. So grounded I'm taking to be someone who's clear headed. OK, there's all the self-care around, sleep, limited alcohol, not being up too late or all of that stuff. So you’ve done some exercise, some nature and maybe some journaling or something. So those kind of morning routines and time. So that clinician who comes into work is, perhaps has got more space than to think about the case. To think, to prioritise, to negotiate, to be compassionate. And of course, each day then brings up so much kind of stuff from the patients, if we're just taking the patients. That's without even taking into account your personal life. You've almost got to sort of every day. You've got to do something with that stuff that you've heard every day and just putting it in a compartment and shutting the box is not, which is what probably what most medics do - slightly dissociate and compartmentalise. As I said before, most doctors don't have supervision and that's just not included in doctor's packages, whereas everyone else gets loads and loads of time for all of that, but not doctors. A lot of doctors do or did drink, put all their stress into alcohol and drugs.

**IK**
Thank you so now looking at kind of uncertainty in the sense of the creative approach. So do you have any examples in the past where you've used a creative approach to help you deal with the uncertainty, so it might be in life or it might be in general practice?

**P2-Dr**
So mine would be kind of journaling and that sort of thing. Yeah, I would like to get into more kind of art and making and stuff, but I haven't really gone there. So it's a bit sort of still quite kind of left brain. I need to get more into sort of bit more right brained stuff. I used things like mind maps or colours. I've always got different colour pens with me. So actually when I'm writing notes, even in my diary, I often I just use different colours. Actually I want to do more kind of doodling and move into that. And what I am doing now is trying to really prioritise the art. So trying to book more theatre and poetry and use that as self-care whereas in the past it would be like, “Oh that's too expensive, I can't do that. Oh, that's too much time”. Now I'm like, that is how I’m going to deal with that and that's what brings me life. You need all of that stuff. That's quite new for me.

**IK**
Thank you. And now I'm just thinking what your view is on how might creative approaches be helpful in clinical practice?

**P2-Dr**
Oh, I think there's loads you could do. I mean, like for a team putting yoga at lunchtime, getting artists and arts in the surgery, poetry in the surgery. Arts and prescription for patients giving poems or psalms for patients. Yeah, so things like that. Having more creativity in away days. Most of the NHS has not thought about this at all. Now whenever I've got a way day I do often ask now if I could read a poem. And often people are shocked and they aren't quite sure how to respond because it's so abnormal. It's so dry these two hour meetings. They’re so dry. That's why I love Louise's symposiums and what you can do in 2 minutes.

**IK**
What about the differences in those normal away days and the symposiums or other things you do, what's the difference?

**P2-Dr**
Oh, they just completely different. You feel completely different. It’s like life. It brings you to life. And it's so simple really. And it's not time-consuming the way it is incorporated properly. Even online, it works remotely.

**IK**
Thank you and now looking at the the flip side. So how might creative approaches be unhelpful?

**P2-Dr**
I can't think, I can't think how they can be unhelpful. I can't really think how they could be unhelpful. I know you're preaching to the converted here.

**IK**
Yeah, very true. Thank you. And now if there's anything you'd like to add from the previous stuff, you know, on creativity, on clinical practice and if there's anything else you'd like to have shared or you think that I could have asked you where this I kind of didn't offer that space or that question? So is there anything else you'd like to to share regarding the role of creativity in tolerating uncertainty in clinical practice?

**P2-Dr**
I think we covered everything. But yeah, it's actually very stimulating, Ivon, to talk about this. I need to write some of this down properly.
