# Supplementary material for: How does creative enquiry enable us to deal with uncertainty?
Source: BMC Med Educ. 2026 Aug 8;26:1785. doi: 10.1186/s12909-026-10060-w (PMC13732821; doi:10.1186/s12909-026-10060-w)
Supplement: Supplementary file 6 [file 12909_2026_10060_MOESM3_ESM.docx]

**IK**
What does creativity mean to you as a clinician?

**P3-Dr**
Well, that's a really good question. It probably means different things to me in different parts of my life. But as a clinician? I think creativity means being able to personalise the care that I give to the patient and to create an individualised plan for them. And for me, creativity means being different. So doing something that's maybe out of your comfort zone or doing something that makes you enjoy and that will make you happy. And I think in my clinical work, it's probably making sure that the patient is happy with their treatment and that it's right for them. So that’s clinically what I would think about. Also, creativity outside of just the patient interaction would be with my colleagues, within the team and having time for reflection and I think creativity can really help with that side of things. And I think everybody's different in the way they want to do that. Personally I like actually making things. So I do ceramics and I find that as a way of having time to myself to reflect on that clinical work as well as make something.

**IK**
Right. You stressed at the start that might have other kind of definitions of creativity for other parts of your life. So how do they differ in that sense or what are they?

**P3-Dr**
Yeah. I use it as a stress relief. My work that I do with making ceramics, I use as a time to think more, be focused and be more mindful about things. I also think a part of being creative is allowing that space just for yourself.

**P3-Dr**
A lot of our jobs are thinking about other people, and I think the time I spend outside of work is making more time for me, which I think is important. I think within work you can be creative in so many ways with your patients and within your team. I think it's more about finding ways of working that are helpful, happy and innovative. It's not just about the artistic side of things.

**IK**
Thank you. And now could you talk about an example where you've used creativity in the past to kind of reflect on clinical practice?

**P3-Dr**
In university I did a similar project to what the students at Kings do. You talk to patients and hear the patients’ stories. And I remember I spoke to a patient for the first time who was towards the end of their life and I remember the story was really vivid and moving for me about his interaction with medication and how he felt about that. And then I used the story to create an art piece about it that involves sort of his face and medication sort of mixed together. I found it really helpful to kind of portray his story and also for me to process that and understand it. I think especially when you're training, you're not used to hearing a lot of emotion and sadness and trauma that people go through. So I think that helped me process those kind of feelings earlier on in my career. It was a sketch drawing that I did and I guess looking back, it was a negative interaction that I had with him in terms of how I felt afterwards, because I just felt sad and upset about his life and the way things were. I also was having that feeling of not being able to help very much. And so I think that process of doing something creative afterwards definitely helped, but like the transition from that part of my life to now when I'm using ceramics it is a bit different. I think it's more of an escape and for my well-being, I don't necessarily do the ceramics based on a patient interaction. It's kind of a more of a formative process for me to just think about things as I'm doing it. I wouldn't necessarily make a piece specifically about a patient. It's more about the process.

**IK**
Yeah, thank you. Could you describe a time in your life that was uncertain?

**P3-Dr**
Yeah, I think it's definitely throughout life for sure and that you can't separate them and I've experienced it a lot clinically. My worst time was when I qualified as a GP. You come out of training and you've had all this time where you've always been supported and you have meetings every week with a supervisor and talk about patients and have a support network there. And then suddenly you're let loose and you don't have anyone to turn to anymore. And I found that uncertainty really difficult for about a year whilst I was like building up more experiences with patients. And trying to understand how I felt happy in my risk management of patients, what I felt comfortable with because it's different for every doctor, especially as GP. You carry a lot of risk with these patients, a lot of uncertainty when you're not totally sure of what the diagnosis is and you kind of have to make yourself feel comfortable that you won't always know and that's OK. And that was a big learning curve for me. Definitely that year after training.

And I guess in other parts of my life, there's been lots of different uncertainties, you know, around where I've been wanting to live and moving house those kind of things. You just never know whether they're going to come through or not. I found that difficult to deal with. Yeah, all sorts.

When I moved from training to qualifying, I think what helped me was that I kind of had a few months where I felt really awful and thought, “Is this for me? I think I'm going to have to quit”. Reframing it in my mind and turning it around and think, “OK, what can I do to help myself?” and gave myself the best chance to keep going. I tried to spread my workload out over more days so I didn't have to work such long hours. I also set myself up for mental schemes where I had a mentor who would support me with any sort of clinical or more difficult case is that I wanted to discuss. And I also joined a Balint group. It’s a psychological intervention where GPs or doctors come together and discuss cases. Usually a psychologist is there and you bring the case, you tell the case to the group and then you then step back and then the people in the group discuss the case. You discuss the ins and outs of it and all the different perspectives, and then you come back in. And that's been really helpful for me managing your emotions around patients.

And then also when I would had been doing that transition, that's when I started doing more ceramics. So I made sure that every Wednesday afternoon that was my dedicated time to do that. And I wasn't doing anything to do with GP and I really needed that difference in my day and time where I wasn't thinking about patients and I could just make something and be proud of that and use like a different part of my brain.

**IK**
Do you think the uncertainty has reduced or is it just there in a different form?

**P3-Dr**
I think it will always be in my life. I don't think that would be a time where I don't have uncertainty. So I think I just have to have find ways of helping me manage it and there'll be periods where I'm in now clinically doing things and I feel confident and good and that might last a few weeks. But then there'll be a few weeks where I feel really uncertain and worried with my own mood and the type of patients I might be seeing. Maybe they're more complex that week. Maybe they're more emotionally difficult. You never know when that's going to happen. So I think it's good that I've sort of built in these strategies of managing that for myself.

**IK**

Thank you. And I think we've kind of already talked about but how do you typically approach uncertainty?

**P3-Dr**
Speaking to friends who also do medicine about any difficult times and normalising it with other people that have gone through similar experiences is really helpful. And I'm lucky that I've got friends from GP training and from medical school I can do that with. And having that support network has been really important.

**IK**
Yeah, great. And now just reading out a statement to you. So in a scoping review, there'd been multiple studies and that have shown that creativity can enhance tolerance of uncertainty. So what are your views on this and why?

**P3-Dr**
I think that's something that rings quite true to me. Doing something creative makes you realise that things aren't black and white. And I think when you live in a world like medicine, where things are very binary, a lot of the time or data-driven or guideline driven, you feel under a lot of pressure. And once you realise actually there's a lot more nuance to life and patience, and there's a lot of grey areas even within guidelines. That helps you actually feel that you're doing a good job or that you are managing things well in your own way and that everybody does things differently. And that's something we should celebrate rather than something we should condemn. And so I think creativity helps with that because it reminds you that life isn't binary. That everybody has a different perspective and that we'll see things differently and that's a really positive thing.

**IK**
Yes, thank you. And how did the ceramics help you deal with your emotions or deal with uncertainty?

**P3-Dr**
Yeah, I think at the beginning I did it with a friend who's also a GP. And so when we did it, we used to discuss all day over making something. So then I'd get the benefit of having her opinion and reassurance and then also being able to make something. It’s calming when you're making something and fully concentrating on all the emotions about what you're going through going through your day. And then after she left and I carried on by myself. It's probably just a physical action of using clay because it's quite physical in that you have to knead it and press into it. So that's probably helpful for getting out any frustration feelings. And then the actual process of like creating something. I love it because you don't know what's going to turn out at the end yet and you never know how exactly it's going to look. I like that there's always uncertainty with making clay, but it's really thoughtful and positive, and it makes you feel good and that you don't care as much. And I like that about it.

**IK**
Why would you want to do something that's uncertain when you're trying to deal with something like uncertainty?

**P3-Dr**
Yeah, it doesn't sound quite correct, does it? Yeah, I think it reminds you that it's normal and it's OK and it's reassuring and calming rather than reading more like facts and guidelines and maybe feeling overwhelmed by that.

And it is a stress relief. Like, I can't imagine reading a guideline and feeling calm afterwards. Doing something creative like ceramics, I feel really good afterwards and I feel proud of what I've done. The other thing as well that I love about it is that it involves other people at the end. So I'll give it to a friend for a gift or I'll sell it and make someone else happy. So I think I love that process as well.

**IK**
Yeah. So the sharing element of your work with others. It's way to release your stress. It's helpful give you your own time, but also the sharing element of it is something you enjoy. So I think we've already talked slightly on it, but how my creative approaches be helpful in clinical practice?

**P3-Dr**
I think it always helps with team bonding at my GP surgery. It would be so nice if maybe we could do or do ceramics together or create something and it just gets you thinking about people's lives that are outside of work. There's so much more than just clinicians as everybody has other interests or other parts of their life that are important to them and it makes you be able to share those things with them. When you create those bonds in your team, it's easier to then work together clinically. So I think that's really helpful. I think it helps with stress relief. Yeah, I think that's all I have to say on that.

**IK**
Yeah, and on the flip side, do you think that creative approaches might be unhelpful?

**P3-Dr**
I think it's always a useful space and a good use of time.

**IK**
Yeah. OK. Thank you. And just the last question, really like if there's anything else you'd like to add?

**P3-Dr**
No, I think I've said you've brought out things to me that I wasn't like aware of and not thinking about before. So that's really nice.

**P3-Dr**
I always think about the future and how as clinicians we can incorporate more of this more into our work and that's my hope. For the future that will, people will be able to use creativity as the day-to-day part of their workplace and I would love to do that more and I just hope that that can be done. That's important.
