# Supplementary material for: How does creative enquiry enable us to deal with uncertainty?
Source: BMC Med Educ. 2026 Aug 8;26:1785. doi: 10.1186/s12909-026-10060-w (PMC13732821; doi:10.1186/s12909-026-10060-w)
Supplement: Supplementary file 7 [file 12909_2026_10060_MOESM4_ESM.docx]

**IK**
What does creativity mean to you as a clinician?

0:0:50.400 --> 0:0:51.880
**P4-Dr**
I think creativity is an ability to kind of think outside of the kind of more formal or rigid box. I think it's an opportunity to bring a bit of who you are as a person or a professional. A bit of the your personal life into most of your professional life and marry the two. I also think it means an ability to express yourself as a clinician, which in terms also helps you to kind of connect with patients on a more human and real level.

Also, when challenges arise or when there's murky waters in clinical care, such as difficult case or a difficult circumstance that perhaps your scientific education doesn't prepare for, having that creativity helps you to approach that situation in a more holistic sense. And actually I think it's really beneficial for this current climate of clinical care.

**IK**
So is it that you're creative normally in your in your personal life or is that you think that applies to everyone?

**P4-Dr**
I think everybody has got their own because I guess I it's when I speak with medical students or patients or clinicians or whoever they are, I always think that one case scenario in a book won't be the same as how anybody else interprets it and everybody brings their own kind of uniqueness to a situation, whether you're a patient or a clinician. It’s about bringing the sense of who you are to a situation and of course, I think recently I've reflected not everyone might be as comfortable. I think we're doing that more openly with others. But I think in every person's life they are probably quite are creative in lots of different ways and there's there's not a hard and fast kind of I don't think definition. And so I do think that everyone has the potential.

0:3:29.500 --> 0:3:42.780
**P4-Dr**
Oh, I think he's already doing it, but it may not fit like other people's definitions of it. But for my definition, which I think is quite open, it's that sense of kind of almost in being able to express it an individuality, I think.

0:3:44.510 --> 0:4:6.30
**IK**
Thank you. And now really talking about an example in the past where you used where you've used creativity. So could you talk about an example where you've used creativity in the past to help you, you know, reflect on your experiences, it might be in, in medicine in or it could be just generally you know?

0:4:6.810 --> 0:4:18.930
**P4-Dr**
Yeah, yeah. I think I've got lots of examples. I guess one that sort of springs to mind is probably my the current role I'm in as an educator here at kings and I lead the clinical humanities.

0:4:20.610 --> 0:4:43.850
**P4-Dr**
Assignment, but I guess it's a core assignment and I do it alongside all of the other things I need to do in year 2 for delivering the GP programme, which of course is a lot of like clinical experience as well as well as kind of lectures and small group works and stuff. But alongside this I run the clinical humanities assignment where students have a chance in the Community to talk to patients and express.

0:4:44.530 --> 0:5:16.770
**P4-Dr**
The themes of their learning, more like qualitative information in a kind of creative way. Now obviously the the students do some amazing work as part of that from poetry and arts and sculpture. But I think for myself, just running that programme I think is is one of the reasons where I think creativity is not only like I obviously believe passionately in it in terms of like as an educational kind of strategy and as a course. But for myself, what it's brought to my own career as an educationist is I think I've learnt more there.

0:5:16.970 --> 0:5:30.130
**P4-Dr**
We have perhaps when I've set up a clinical scenario looking at like a urinary tract infection or a kind of, I don't know, eczema scenario is that.

0:5:30.170 --> 0:5:43.450
**P4-Dr**
With that has come like almost. It's like I'm learning from it in a way. What to hope the students would also learn. And it's funny, reflecting on it that I guess I must say, one of the main things is I think it's probably some of the most meaningful work I do.

0:5:45.10 --> 0:5:45.890
**P4-Dr**
Asides from that.

0:5:46.210 --> 0:6:14.770
**P4-Dr**
I think it is creative because it includes a lot of like I think being creative in a bigger forum is sometimes quite risky. So I think in terms of it has been a risk sort of educationally and as part of my role, which actually then when it works out well, bring some reward. And if it doesn't work out so well, which at times it is difficult to navigate, getting that sort of thing into the curriculum, it's almost the challenge is more like a passionate challenge that you want to do.

0:6:15.280 --> 0:6:18.160
**P4-Dr**
And then I think it is the output is that.

0:6:19.600 --> 0:6:48.960
**P4-Dr**
You know, sometimes with my other courses that I run, we get like a some nice evaluation data with bar charts and things like that. And I think, OK, that's great and you know it's nice to know 99% of students really liked learning about, you know, basic life support. But with this course, despite like the sometimes more mixed feedback. So some students won't see the benefit perhaps. And I think in year two that's can be understandable because it's so early on in like basically personal and professional life I think.

0:6:50.160 --> 0:6:54.520
**P4-Dr**
The the outputs that students that kind of do make that journey.

0:6:56.0 --> 0:7:22.200
**P4-Dr**
Provides is just so lovely because in a way it makes me reflect on clinical care because I sometimes have also forgot forgotten parts of it as a. As a busy clinician, like for example things like the jargon we use, they mean the most meaningful contact being you know how somebody first received, you know, receives a patient and when they've had to break difficult news. And I've seen that more in like creative output than reading it or discussing it.

0:7:23.520 --> 0:8:4.400
**P4-Dr**
It seems more meaningful. Also, it's really nice just to see a lot of more visual things or and have people reflect around those objects rather than kind of, you know, more almost sort of boring written reflection sometimes. Shouldn't say boring. But you know what I mean? Like, it brings it to life where someone's also got a piece to talk about and then the process is really interesting and engaging. So yeah, I think those things sometimes shock me because I think, oh, you know, I was passionately believing the students and their potential. But when I see that every year, I think to myself, it is really meaningful to do this work, to be more creative, even as my in my career as a medical educationist.

0:8:4.920 --> 0:8:31.600
**P4-Dr**
Because it's not not only the students that gain things, but it's our patients and and me, actually I'm sort of getting the secondary benefit of and then that spurs me on to do better as a clinician, as an educator and I don't think I have that same feeling with any of the other courses that I run that are all very meaningful. I work as hard as I do on this on that. But it there's something about that more potential to be creative that seems to inh order for me.

0:8:33.240 --> 0:8:34.520
**P4-Dr**
Give so many benefits I think.

0:8:35.410 --> 0:8:35.890
**IK**
Yeah.

0:8:35.570 --> 0:8:38.530
**P4-Dr**
Sorry, I don't think I answered the question. I think I did.

0:8:37.960 --> 0:8:54.440
**IK**
No, no, no. I think I think you're right. If correct me if I'm wrong, but the idea that whilst other people are kind of doing this creativity in the in that course, the creative humanities course, it allows you to reflect yourself and see free see it from a different perspective.

0:8:55.150 --> 0:9:31.670
**P4-Dr**
I think so. I think so. And then with our team as well, we work with a lot of artists and it's it is really refreshing to see like the other side of what having a different skill set there brings to how we approach things. Whereas sometimes when you're with all doctors all the time, we can all become quite like in a little bubble of kind of Oh yeah, that's a good idea because we all are sort of trained in a way to think and behave the same, whereas this is always like a really good reminder almost of our patients that actually they may not perceive everything that we think is a great idea is their idea and like it does make me reflect on sort of person centeredness.

0:9:32.940 --> 0:9:35.540
**P4-Dr**
And student centeredness I think at the same time.

0:9:34.780 --> 0:9:51.260
**IK**
Yes. And you said you had other examples. So not just this, but has there been times when you've actually, you know actively done the the work or stuff yourself and has that those benefits been different you know compared to looking at others?

0:9:51.840 --> 0:9:55.0
**P4-Dr**
Yeah. No, I think when I do it by myself.

0:9:56.550 --> 0:10:27.350
**P4-Dr**
I'm if I'm honest, I though I love. I really love the arts. I'm doing it for my own well, usually as a well-being thing, so more of a stress relieve. So the things that I do are so I love the art. So on a Wednesday there's this watercolour evening at our local pub and I think it was, they're a really friendly, like community orientated pub and I think the idea is just bringing people together from the local community and doing some kind of art so they don't like run a lesson or tell you how to mix your paints or anything.

0:10:27.550 --> 0:11:0.550
**P4-Dr**
They just have, like the food, drink and I'm sure they benefit from that as well. But then they have loads of different water colour sets. Me and my friends go there and often go on Wednesday, which is the busiest day of my clinical week. So I do full clinic on a Tuesday and then on Wednesday I'm at the university and it's usually the day where we do a lot of meetings and strategy and I feel like my brain is totally saturated by the end of the day, I can't make any decisions. I feel like that feeling. You know, when you go and do an Oscar at the end, you're like, I don't think I have anything more to give and I can't make sense of anything.

0:11:1.890 --> 0:11:32.250
**P4-Dr**
And then doing that watercolour night, I think it is like it's mindful, it's kind of a chance to connect again and remember that I actually I really like used to be someone quite fun and I used to like, you know, and it's weird. It's like let's you reflect on, you know, what the day has been. And then I think that it's almost feels like your kind of veil is taken off and you can be human again. Just doing that kind of activity. And then it makes me actually see. So I always have a thing in my head that I don't make any.

0:11:33.50 --> 0:11:34.810
**P4-Dr**
Rush decisions on Wednesday because.

0:11:35.560 --> 0:11:52.560
**P4-Dr**
I feel like I am quite saturated by then with everything, so if I've seen an e-mail at work or I need to think about an exam or some assessment or something, I need to do, I think to myself I'm going to park it until after Wednesday and part of that rehabilitation for myself is going to that watercolour thing and drawing.

0:11:54.120 --> 0:12:7.600
**P4-Dr**
And I think also I've surprised myself that I haven't lost all of my skills because I did it for GCSE and I got an A star and I really wanted to do it for a level and I guess part of it is you do think oh actually I can make something and it looks vaguely nice and you get a little bit of a pat on the back from that.

0:12:8.160 --> 0:12:39.920
**P4-Dr**
But I think it's more of their well-being side that it just swapped like engages a different part of your brain which can be active at those stressful times. So I think I've done that. The other thing I do is like I'm I really like going to the tape to I'm like a member there and I got it first as a present. I think after the pandemic, just to kind of help the arts in the post pandemic way. But I've really found it almost like a really nice place to go by myself without any friends just by myself.

0:12:40.280 --> 0:12:44.80
**P4-Dr**
To have that quietness in their museum space, but also.

0:12:45.760 --> 0:13:13.840
**P4-Dr**
To just yeah, to kind of I would find over a lot of things overwhelming still and I think in terms of become at times anxious with being a mom and the Pope. You know, I think we all affected by the pandemic and I think just going and reflecting in that more sort of creative or artistic space almost gives you again that freedom to relax and it and be your sort of self and that's more it's not shared with anything it's I don't know. I don't think I've actually reflected until you've asked me this actually.

0:13:14.610 --> 0:13:15.90
**P4-Dr**
So.

0:13:29.0 --> 0:13:29.360
**P4-Dr**
Yeah.

0:13:16.40 --> 0:13:31.920
**IK**
No, thank you. So, yeah, the the idea of creativity can be doing the watercolours and going to the Tate Museum can be a stress relief, as many loads of people say gives you that kind of break from your normal work.

0:13:33.640 --> 0:13:37.480
**IK**
And then also kind of refreshes you to go back if I'm right?

0:13:37.200 --> 0:14:10.520
**P4-Dr**
Yeah, yeah, definitely, definitely. And I think it's quite mindful 'cause, you know, everyone's always like you should practise mindfulness. But The thing is when you are trying to paint, you have to be mindful. It's like you can't do your phone at the same time or take a call or, you know, try and really multitask. I even tried to like once drink a glass of wine and spilt it all over the table because I think it is. You have to be concentrating on the what you're doing and then and so and myself and my friend who go realise we can't really even we sometimes chat. But if you start chatting even.

0:14:10.930 --> 0:14:24.610
**P4-Dr**
That, and that's a lovely thing that you share that creative with someone you actually can't do it. So there have been periods of time where we were silent and for me that is quite hard to be silent. I find silence is quite difficult in a way, so.

0:14:26.170 --> 0:14:34.890
**P4-Dr**
I might be the ideal interview person, but yeah, I yeah. So even having that makes me actually practise it in a way that is more.

0:14:36.410 --> 0:14:50.730
**P4-Dr**
I think it's spoken to me better than any of the other sort of way that mindfulness has been taught. You know, like doing that grounding exercise and all this. I think more when I do painting then I feel it's mindful. Like I really think about how what I'm holding and what it sounds like and.

0:14:51.60 --> 0:14:52.540
**P4-Dr**
Being in that moment and stuff.

0:14:53.270 --> 0:15:12.950
**IK**
Yes, thank you. And now kind of looking at the other side of the the interview, which is the uncertainty. So I'm sure you've you've had loads of uncertainty in in your past, but could you describe it could be possibly a time period of your life or?

0:15:14.510 --> 0:15:24.550
**IK**
A place that you kind of as soon as I tell you the question, probably you'd say, oh, that was very uncertain time in my life. It could be in clinical practise.

0:15:25.30 --> 0:15:25.470
**P4-Dr**
Yeah.

0:15:25.280 --> 0:15:28.480
**IK**
And and then also generally in life as well, so.

0:15:28.560 --> 0:15:37.200
**P4-Dr**
Yeah. OK. So you want just to discuss that on period of uncertainty or with a link to creativity or you're not, it doesn't matter?

0:15:38.990 --> 0:15:39.470
**P4-Dr**
Yeah.

0:15:42.950 --> 0:15:43.430
**P4-Dr**
No.

0:15:36.340 --> 0:15:45.420
**IK**
I think we'll we'll be linking it to creativity later, but if that helps you to talk about that now, then of course, but it's just kind of breaking it one at a time.

0:15:45.230 --> 0:16:13.510
**P4-Dr**
Yeah. So a period of uncertainty, I think probably like lots of people is probably the pandemic. And then on top of the pandemic, I had this kind of slightly quite difficult situation where I kind of was there at the beginning of the pandemic like heavily pregnant. And then I had my second child, like literally about six weeks after Boris Johnson went to Saint Thomas's and was intubated. Then I was going there having a baby.

0:16:13.820 --> 0:16:45.500
**P4-Dr**
So I think it was probably that's the most uncertain time, like also at the same time like I had a bereavement of my my mum passed away like not from COVID but six weeks prior to that. So there was a time where I really honestly felt like I didn't know what I was doing. Like I I did know. But for the first time in life, I always had a plan. I was kind of knew what the next steps were, what I was going to do. But it was now this time when I was having a baby in the middle of the pandemic, then I probably needed to return to work after.

0:16:45.540 --> 0:16:46.100
**P4-Dr**
Maternity leave?

0:16:52.620 --> 0:16:52.780
**IK**
What?

0:16:47.0 --> 0:17:0.480
**P4-Dr**
And they couldn't really, sort of. I had to take every day as it came, and they couldn't. They couldn't. I wasn't sure what the future would bring. 'cause. I'd sort of been knocked off, I think on multiple levels, and I just couldn't see through.

0:17:1.840 --> 0:17:19.400
**P4-Dr**
I think I was thinking about. I remember at the time, yeah, I'm going into the creativity, but do you know that book by Charlie Mckeesy, the one about the the boy, the mole and the horse or whatever it's called the fox the it's like these amazing watercolour pictures. And I think he was an artist that in the in the pandemic did quite a lot of stuff.

0:17:20.50 --> 0:17:42.170
**P4-Dr**
I found weirdly his work during that time, like the only thing that sort of got because it said, you know, it was like motivational in a way that you could, like, see the word for the trees and all of those sort of and see the light, the under tunnel, those kind of things I think were which get got me going. Like we didn't know which path that would be, but hopefully there would be a light somewhere. But yeah, I think in my time.

0:17:43.570 --> 0:17:55.570
**P4-Dr**
That was probably the most uncertain thing that I've ever faced like that. And then I think by the time I returned to work, things were quite uncertain, but I'd found my own ways to deal with it. I think I had a bit more.

0:17:56.210 --> 0:18:25.970
**P4-Dr**
Information or I'd had sometimes do some work on myself. I guess with with everyday clinical work, I face uncertainty all the time with patients like the climate of the NHS. At the moment I am actually a bit worried about, well, not a bit worried but like everyone, the state of the NHS in the hospitals, I feel like nothing. I honestly feel sometimes with secondary care. It's no fault of anybody working so I can get care. I think it's a result of funding and the political comment we're at but.

0:18:26.610 --> 0:18:29.370
**P4-Dr**
I actually tell patients like, I'm not sure.

0:18:29.900 --> 0:18:48.20
**P4-Dr**
What is going to you know when you're going to get that appointment? What the next step is we we're having trouble kind of either getting that information or we're going to try and do it slightly differently, but I couldn't. Unfortunately, I can't tell you when that will be or when your your operation is going to come because there's so many different things going on.

0:18:49.260 --> 0:19:0.940
**P4-Dr**
And then again, sometimes I with budget cuts and things services that I thought actually we could get you sorted in two weeks and would be clinically really good. I can't rely on those, so.

0:19:1.360 --> 0:19:16.880
**P4-Dr**
It's more like. Then it comes back to you, especially in general practise, you kind of holding that uncertainty because there's no one else can give any more information to to move that sort of clinical uncertainty forward. And it's almost comes back to you and that sometimes also about risk again I think.

0:19:32.150 --> 0:19:32.510
**P4-Dr**
Yeah.

0:19:17.900 --> 0:19:36.780
**IK**
Yes, and thank you. And and now I think you've already discussed kind of as you were talking through, but in those kind of experiences of uncertainty, how do you typically approach it? So it's just generally how do you how would you approach well, how did you approach it in those times you know?

0:19:36.790 --> 0:19:57.350
**P4-Dr**
Yeah, I think it's really hard to like when you're really stuck. I think it is about slowing it down a little bit. So sometimes you can think, oh, because everything's so pressured, there's this feeling of everyone, like, what's going on, what we're going to do. And sometimes you put that in yourself. I think sometimes it is like slowing down and trying to really sort of unpick what's going on.

0:19:58.360 --> 0:20:15.800
**P4-Dr**
I guess in on a flip side I'm quite a problem solver, so sometimes I think how would I get myself out of this and make it step plan? But I guess when you're really feeling uncertain, you don't really know what that plan is or you can't make a plan, so sometimes it's just sitting with it, which I think.

0:20:17.400 --> 0:20:29.480
**P4-Dr**
Does help. That's why I like looking at art in a bit and I and I don't like them more precise art. So like literal paintings, I like the abstract side because I like the messing there.

0:20:29.880 --> 0:21:0.40
**P4-Dr**
Actually, I like the messiness of life, and I think I've learnt with time to think that sometimes the messiness is OK, you're like, you know, like, I've I wasn't always like this. But with time and I think their speciality, I've chosen having kids having bereavements, having ups and downs, I'm kind of OK with it not being perfect or I'm OK with not always knowing the answer despite my Nat. Also on the flip side, my natural thing to problem solve, I think that's you know, I like diagnosis and medicine and.

0:21:0.640 --> 0:21:3.760
**P4-Dr**
Making sense of things, but then I also quite like.

0:21:4.850 --> 0:21:9.170
**P4-Dr**
Just sometimes going, I don't know why that's happened and that's a bit bonkers, but that happened.

0:21:10.390 --> 0:21:31.350
**P4-Dr**
Could we learn something from it? Yes, maybe. OK, we'll take that. Could we learn, like, if anything, never to do that again? OK. And sometimes I don't even know how to. I sometimes I talk with colleagues and that's another way us just going. I don't actually know what happened there. And I don't think we ever will. And we're OK. I'm OK with that. And so again, I think talking it through.

0:21:32.830 --> 0:21:42.310
**P4-Dr**
Uncertainty sometimes helps because it either might help you to kind of feel a bit more certain or give you that confidence to feel like that. Or it might be that someone else also goes.

0:21:43.430 --> 0:22:5.630
**P4-Dr**
I would feel very uncertain about that and I think validating your feelings sometimes helps, but I'm sort of like I do like the messiness of life. I think that's why I chose general practise. I like the fact that it doesn't fit in a box. I like the fact that it can be messy. I like the fact that, you know, it can be a little bit out there or eccentric or bit crazy. And I'm kind of good with that. I think now more and more.

0:22:7.590 --> 0:22:17.830
**IK**
I think you're already kind of talking about this idea of creativity as one of the ways you approach, but of course, sharing with people, talking with people.

0:22:22.300 --> 0:22:22.780
**P4-Dr**
Yeah.

0:22:19.270 --> 0:22:27.630
**IK**
Other ways as well of trying to deal with it, but there was I think two key things that you you said, which were really interesting.

0:22:29.920 --> 0:22:34.880
**IK**
And I just want I think you already said you kind of it was based on the art you do but.

0:22:36.760 --> 0:22:39.720
**IK**
I think the first thing you said was.

0:22:40.200 --> 0:22:44.160
**IK**
You're very clear with your patients, you know, saying that you're not sure.

0:22:44.360 --> 0:23:0.480
**IK**
The outcome or when your next appointment will be or things like that and at the same time, I think during or using the art, it allows you to kind of appreciate the messiness and accept it as it is, so.

0:23:2.20 --> 0:23:9.860
**IK**
It'd be great to hear a bit more on on that. So how are the art kind of allowed you to accept the messiness and?

0:23:9.520 --> 0:23:10.40
**P4-Dr**
Yeah.

0:23:11.130 --> 0:23:13.770
**IK**
Did did the art actually help you at all in you know?

0:23:15.320 --> 0:23:25.960
**IK**
Yeah, in your approach in, you know, asking patients or telling patients that being clear with them and saying what I'm not sure, but that's kind of something. I don't think everyone would say.

0:23:26.340 --> 0:23:27.980
**P4-Dr**
Yeah, yeah.

0:23:27.300 --> 0:23:28.220
**IK**
So yeah.

0:23:29.380 --> 0:23:45.380
**P4-Dr**
Yeah, I mean, I guess it's more like, yeah, I think that's more from the fact that I think look more I think looking more at art and doing my own thing and seeing other people's interpretation like obviously I look after 200 students and.

0:23:46.940 --> 0:23:57.420
**P4-Dr**
Everyone's interpretation of the project I lead is different and as well. When I'm doing my own art and then with my friend, I'm like I see how different the thing we've chosen to paint is the style, the.

0:23:58.140 --> 0:24:0.100
**P4-Dr**
Movement and actually.

0:24:1.760 --> 0:24:30.600
**P4-Dr**
None of that is rock. None of it's wrong, and I think there's this obsession with medicine, and I think for medical students, and I understand why, because of oskis and how exam driven we all are and I think it's our fault as educationalists that we drive that home with students that you have to get certain Marks and and behave a certain way is that sometimes it's people are searching for the right way to do it or you know there's sometimes like a really clear wrong way which is usually like in patient care like unsafe care or.

0:24:31.280 --> 0:24:32.200
**P4-Dr**
Unprofessional. Unprofessional care.

0:24:32.650 --> 0:25:3.930
**P4-Dr**
But other aside from that, there is no real right answer to do it, and so I think that's what I see all the time when we do things in museums and we get students to do this thing we call visual thinking strategies, looking at paintings. And I I think I already did this, but reading more about it has made me think, oh, you know, when we first look at something, we often zoom into one bit and we don't know why. And then sometimes you take a look back and you see something different and then some other times you're like, don't know what that is. I think that is also.

0:25:4.10 --> 0:25:31.770
**P4-Dr**
Something that we do in clinical care all the time, so I might as soon as patients has chest pain, I might try and do a spot diagnosis just based on my own, probably bias about, oh, they look slightly overweight and the smoke, I don't think they're probably having a cardiac chest pain. But then when they're saying something else or they say it's actually epigastric or they're is explaining something else like I'm putting that information together a little bit and then they might mention something. Totally other thing that I'm like actually I don't know what that is.

0:25:33.350 --> 0:26:9.350
**P4-Dr**
So I think sometimes like I do see I see the the sort of the the parody with like looking at different kind of situations that can be mirrored in art. And I don't know which one happened like I think I'm able to do both because I'm also thinking about how to how to teach it. So I think about my process as a in clinical reasoning or something when I'm making a diagnosis and I'm able to link that to the art. And then when I see the art, I sometimes think about, oh actually this is like patient care or it's telling stories like a history or something. I don't know which one came, which one came first. I think they probably enhance each other.

0:26:9.900 --> 0:26:42.500
**P4-Dr**
I think the messiness thing is 'cause. I have naturally quite mess. I'm not one of these people that, like has perfect handwriting and kicks everything in the box, and I don't. I'm I think some of that is personality driven, but I think that has led me to kind of then find art or creative means that match my my personality. You can find some very specific like very sort of neat art. Can't you like graphic art or, you know, things that are really pointless and is like, I actually don't like pointilism because I think it's too.

0:26:43.240 --> 0:27:13.0
**P4-Dr**
Nice. So I think you can find something to match your own personality and then bring with that to clinical care. So it doesn't mean that you have to love everything. I think also like mentors here who are into the arts. I think those that are naturally creative are also OK sometimes with saying that you don't have to either know that this is good for you right now with teaching the arts. But also like you don't have to know the answer. And I think that's all given me confidence with the fact that I see them as like.

0:27:13.390 --> 0:27:30.830
**P4-Dr**
Mentors or role models, and they've managed to get to a high place in their career or not high place. But you know somewhere you feel like you would like to be and they've done that by being a bit more vulnerable. Saying I don't know and not losing their power or whatever. So I like all of those things and I think about more and more about the arts and inclusion.

0:27:32.670 --> 0:27:49.270
**P4-Dr**
I think like I said at the beginning, I think everyone has the ability to be creative in lots of different ways because I think that's human nature goes hand in hand with creativity and that's why I like kids normally paint at school, don't they? And no one judges when they're, like, making when my kids are my kids, are doing these scribbles.

0:27:49.780 --> 0:28:14.620
**P4-Dr**
Because it's a way to express at that age and we've we all do that we can all do that, but to different degrees like I think later on we cast our own aspersions on what's better and then others. But I think every person that has that, but I think recently with the arts and creative and inclusion, I think a little bit about how we can include all of our students and even the arts for me.

0:28:15.900 --> 0:28:20.580
**P4-Dr**
I'm not naturally kind of. I didn't. My parents were into the arts, but they were first generation.

0:28:21.160 --> 0:28:36.40
**P4-Dr**
Immigrants. It wasn't kind of a thing. You either did it and it was like a hobby. It wasn't like anything. Being creative was like, great, if you were going to be an artist or. But otherwise it was a hobby that went along with your job or something like that, whereas.

0:28:38.210 --> 0:29:11.290
**P4-Dr**
I and I think that the way that it's maybe is in this country with like arts being sort of associated with, I don't know higher, I don't know educational status or I don't know what to say in the way our galleries are set up and stuff. I can see that it is sometimes it's quite a turn off to to students and patients. They'll be like Oh no, I never did well at that we it's all about like being very good and being able to put it in the best galleries especially we're really lucky in London to have lots of galleries but when you go around you don't think this is the most inclusive place I've been to town. So I think sometimes that.

0:29:11.330 --> 0:29:14.930
**P4-Dr**
Turns people off because they think they that they haven't had the potential to develop that.

0:29:15.860 --> 0:29:44.820
**P4-Dr**
And now they kind of are being, you know, asked to do this thing that naturally they feel excludes them. And I think when I asked students, have you been to a gallery before? I am quite surprised at how many haven't been. But I can, despite living in central London now, I think that is because of the kind of, I'm not sure, it's almost been the most welcoming sort of place to be for everybody. I don't know, maybe that was a bit controversial. What I just said, but yeah, so sorry.

0:29:43.800 --> 0:29:44.960
**IK**
No, no, I can, I can.

0:29:45.980 --> 0:30:7.900
**IK**
Yeah, of course. And and now we're just moving on to the the creativity part. So I'm just gonna read out a statement. So in escaping review in Escoping review, there have been multiple studies that have shown that creativity can enhance the tolerance of uncertainty. So what are your views on this and why?

0:30:9.270 --> 0:30:38.30
**P4-Dr**
Yeah. I think as I I think I've met managed before, I think when you went creativity and fostering those skills, a lot of as what I've said is like embracing the kind of being able to come and come out of your safety zone and express yourself in the way that you want to. But also the fact that if you go through that programme process, you also more tolerant about that with other people. So I think what people struggle with a lot is kind of other people's.

0:30:38.370 --> 0:31:8.650
**P4-Dr**
Behaviours and things like we often can like just about make peace with our own, but that if someone else sort of seems outside of the box, we find that really hard. So I think it's the tolerance on on of I think it's more like general tolerance from creativity because you you see the possibility that, I mean everybody has a right to express themselves in the way as long as it's not deliberately unkind. So I think you already think that I think with creativity it is about risk taking. So sometimes you take a risk and it might go wrong.

0:31:9.680 --> 0:31:40.560
**P4-Dr**
But sometimes with creative activities it it's in a in a safer zone than clinical care. I think so in a way. Once you've done that, you feel a bit more like, OK, I'll practise that and that was safe. Now when I go to clinical environment, which might be slightly more threatening, you can bring certain aspects of that and be OK with the outcome. I think it's also like that messiness of life. I think uncertainty comes with its normally the more like the human value side. It's like that we feel a bit threatened or.

0:31:40.910 --> 0:32:7.30
**P4-Dr**
We are unsure about the future with that's with the uncertainty we haven't got the right answer and all of those concepts come. I think when you go through a creative exercise that you normally even a tiny thing like drawing with your eyes closed, you will go through that, it will be something that's not the best. You know there won't be a world expert in drawing with their eyes closed. You know there might be, but still there's, you know there's different interpretations. It's something that you don't normally do.

0:32:8.270 --> 0:32:12.510
**P4-Dr**
So I think all of those kind of and they also like just even have giving it a go.

0:32:13.30 --> 0:32:30.110
**P4-Dr**
I think she's a flexibility which I think you need to be able to enhance to kind of deal with uncertainty because the flexibility is like that. You realise that actually a lot of life is uncertain, there is no answer even though we might really want there to be.

0:32:30.150 --> 0:32:44.270
**P4-Dr**
And also this aspect of like you can't control what else is going on. Sometimes you can only control your own response to it. So when you kind of you know when you're creative, I think it is that you can to an extent like.

0:32:46.380 --> 0:33:15.700
**P4-Dr**
Control what you're doing, but it may not come out the way you wanted it to. Or you know, if you if you attribute other things to that like I'm not good enough or this is gone wrong, then it kind of just defeats the whole object of being creative in the 1st place. So I think this being able to give it a chance and have that openness is also good at good skills for approaching kind of the uncertainty that it's like sometimes you just.

0:33:16.540 --> 0:33:17.740
**P4-Dr**
Have to give it a go and then.

0:33:18.900 --> 0:33:23.140
**P4-Dr**
Deal with the consequence or the next steps as as they arrive.

0:33:23.860 --> 0:33:29.980
**IK**
Yes, and and I think there was, I just wrote down like as you were speaking something so.

0:33:35.300 --> 0:33:35.700
**P4-Dr**
Yeah.

0:33:32.520 --> 0:33:40.400
**IK**
I'll ask the first I I just asked someone at a time, but I think you were describing kind of creativity as a process can be uncertain.

0:33:40.890 --> 0:33:41.370
**P4-Dr**
Yeah.

0:33:41.620 --> 0:33:52.500
**IK**
So you, you know, like you're saying you don't know what the outcome is and that's OK you can have a you can give it a go even if you've never done it before. So my question is if.

0:33:53.790 --> 0:33:56.710
**IK**
If you're trying to deal with uncertainty.

0:33:57.470 --> 0:34:9.630
**IK**
Why do something which is uncertain, which is creativity cause a process of creativity is uncertain, as you say. So why do something uncertain to deal with uncertainty?

0:34:9.720 --> 0:34:43.560
**P4-Dr**
Yeah, I guess so. But I guess it's more like practising that feeling and being able to, it's almost like exposure therapy, isn't it? If you if you just like, keep revisiting that same uncertain process, then eventually they're kind of the more like negative feelings associated with that will diminish, will diminish. And eventually you'll sit OK with it. And I think that is a little bit like how it might work in clinical care, you know, or when you take blood for the first time, you absolutely you, you know, I remember trembling and being terrible at it.

0:34:43.800 --> 0:35:19.520
**P4-Dr**
Of course got, but the more times I did that, that became one of the most certain things I could do on renal medicine as opposed to, like, all this dialysis and looking at blood. And then so that's why I think exposure to that, like throughout at different times in a Safeway can help. Obviously if you were like exposed to it in a in a very exposing way like you know they said whatever you create now is now going to be like linked to you for 12 years. You'll never shake it. I think then that wouldn't be working, but I think there's certain things you can do which is like building your tolerance to uncertainty perhaps.

0:35:19.740 --> 0:35:28.420
**P4-Dr**
And you might and again, not everyone's going to love that feeling, but they'll at least practise and be sort of more accepting of it to it, to an extent.

0:35:29.840 --> 0:35:31.680
**IK**
Here and the other thing was.

0:35:39.260 --> 0:35:39.580
**P4-Dr**
Day.

0:35:41.760 --> 0:35:41.960
**P4-Dr**
Oh.

0:35:44.550 --> 0:35:44.870
**P4-Dr**
Direct.

0:35:54.860 --> 0:35:55.180
**P4-Dr**
Yeah.

0:35:58.180 --> 0:35:59.140
**P4-Dr**
Bye bye bye. Yeah.

0:35:33.760 --> 0:36:1.840
**IK**
You were saying that there's certain kind of aspects of their creativity or the art which in which you can kind of translate into your care. So my question my question is how does doing a painting so something like I don't know using watercolours and drawing a picture, how is that going to relate to you talking to a patient? Because I think you kind of said about this kind of exposure risk taking thing.

0:36:1.420 --> 0:36:2.580
**P4-Dr**
So yeah.

0:36:8.630 --> 0:36:9.110
**P4-Dr**
Yeah.

0:36:5.20 --> 0:36:13.900
**IK**
How? How does it help you? You know when you're painting to help, you actually put that into a clinical care situation. Yeah.

0:36:11.860 --> 0:36:40.460
**P4-Dr**
You like clinical care? Yeah. So I'm thinking it's different. So normally I think I like narratives. So I like the the story that goes. That's why I like. I'm not too. I don't take a history now that I'm qualified like a history, I took a Med student at the school where it's a little bit more, let's say scientific, whereas I think. And so when I draw, there is some kind of and painting. There is some kind of story or feeling or.

0:36:41.780 --> 0:36:50.460
**P4-Dr**
Experience that I'm trying to capture, even if it's abstract and with colours, there's like a feeling there, so I think it I think also it's kind of.

0:36:51.60 --> 0:37:20.700
**P4-Dr**
It's almost like an empathy thing, where sometimes you're trying to capture or understand a feeling. So I think sometimes I use that and I think when I'm looking at art, I definitely trying to think of, I think of it weirdly, a story. When I look at anything like what's going on, like, I guess the more abstract that's harder to do. But if I see a photo, say, or I take a photo or I'm trying to capture that same thing, the essence of that, that encounter or that feeling.

0:37:21.570 --> 0:37:55.330
**P4-Dr**
And so I think that makes me reflect on my patient care. When I'm doing those things 'cause I think sometimes it is about that more like they call it soft. I hate that. But you know, the softer interactions in when you meet patients. So it's not about diet. So I think we talked about you said the uncertainty and the diagnosing and stuff, that's all there. But actually just the human connection side, I think and the empathy, that's something that, you know, I was feel I was have a feeling when I'm in clinical practise like it's not like obviously I have a feeling like.

0:37:56.170 --> 0:37:58.210
**P4-Dr**
I know what's going on and that's a great feeling or.

0:37:58.820 --> 0:38:15.180
**P4-Dr**
And I need to check it, but it's almost like I have a feeling about our interaction with me and a patient. Like, did we hear to our for? I think they like me or something about them, they seem sad or you know, it's that emotional kind of understanding, a bit like emotional intelligence, I suppose.

0:38:16.540 --> 0:38:28.860
**P4-Dr**
And that's quite important to me in my clinical practise. I think it's one of the things that I I thought about when choosing a speciality that I want to connect with my patients and learn about who they they are kind of thing behind.

0:38:30.300 --> 0:38:31.500
**P4-Dr**
Just making a diagnosis.

0:38:32.710 --> 0:38:52.950
**P4-Dr**
And so, yeah, I think the human factor is connection. I find this a lot easier to explore through art than it is to kind of read anything or read a journal or anything like that. I think it naturally makes you reflect when you paint and think about emotions and think about how you're feeling and how somebody else might be feeling.

0:38:53.430 --> 0:38:59.910
**IK**
Yes. Yeah. Thank you. And then there was one thing you said, I think a bit earlier on.

0:39:0.420 --> 0:39:0.780
**P4-Dr**
Yeah.

0:39:1.270 --> 0:39:1.990
**IK**
This kind of like.

0:39:3.570 --> 0:39:6.90
**IK**
It can be a parody, almost the idea that like.

0:39:8.930 --> 0:39:16.50
**IK**
When you're when you're doing the drawings or when you're doing things it, it can make you remember a story.

0:39:17.850 --> 0:39:23.770
**IK**
Or a time that you've actually you've had and put that on the paper, or do whatever you're doing so.

0:39:37.790 --> 0:39:38.270
**P4-Dr**
Yeah.

0:39:39.890 --> 0:39:40.370
**P4-Dr**
Yeah.

0:39:25.770 --> 0:39:41.850
**IK**
Could you tell me more of like how how that allows you to put it on the paper or how does it allow you to, you know, remember that situation is that is it, how does it like you were saying just now like kind of not reading guidelines or something, but how does the art actually make you?

0:39:43.310 --> 0:39:46.270
**IK**
Reflect, you know, in a in A at a deeper level.

0:39:47.10 --> 0:40:17.770
**P4-Dr**
Yeah, I think sometimes for you to kind of reflect on a deeper I think reflection is all quite personal, isn't it? So sometimes when you're reflecting clinical care, it's about reflecting on almost like in a more scientific way or we we often are asked to reflect in our portfolio or from our tutor or whoever that is or a boss at work. And we kind of almost can fake can fake it. So you can almost say what you think they want to hear.

0:40:18.970 --> 0:40:54.170
**P4-Dr**
I think the thing when I do art is like I I know that no one's checking it or needing or. I don't need to portray myself in any in this particular way. I I actually just need to do what's meaningful for myself. And that's kind of what we really want. All of our students to do or when we teach juniors is reflect on something that's actually meaningful for themselves and how they could learn or think about doing something different. But I think because of this sort of nature of how we're doing it, perhaps sometimes when we reflect it becomes like this kind of slightly weird thing where we have, like, everyone's trying to answer what we think we should be doing.

0:40:54.500 --> 0:41:16.380
**P4-Dr**
So I think when I'm doing the arts, I don't sometimes don't know how I feel until I start doing it. And I think sometimes when I have that mindfulness moment where then I can clear a bit and then I'm looking at what I've drawn and I'm trying to then be a little bit more critical of myself as like, why was I feeling a bit weird today or what? What is it that sort of being able to relax?

0:41:17.740 --> 0:41:26.660
**P4-Dr**
Helps me to think a little bit more deeper about myself and what I'm bringing to the situation rather than trying to be like more like other people want to hear when we reflect so.

0:41:27.540 --> 0:42:0.60
**P4-Dr**
But I think that is ultimately what we want better. It's like you really think about yourself and why what you've brought to this, what, how others have affected, how actually the situation today is different from last week and that's sometimes quite hard to do because it feels like we don't have the space in clinical care to always do that. But the arts weirdly, something about it makes me think you've got an infinite space to to do that. If you feel psychologically safe. Obviously if you do something exposing that every. But I think it's what you you get rid of the judgement thing.

0:42:0.270 --> 0:42:14.550
**P4-Dr**
For someone watching you or doing anything and it becomes more like personal to you and you feel like you've got this infinite space to, like, reflect properly or deep deeper about yourself because there isn't a right or wrong answer.

0:42:15.140 --> 0:42:21.700
**IK**
Yes, thank you. And and and now kind of looking at these kind of last two questions.

0:42:22.120 --> 0:42:22.440
**P4-Dr**
Yeah.

0:42:23.240 --> 0:42:24.160
**IK**
One kind of looking at how.

0:42:31.790 --> 0:42:32.270
**P4-Dr**
Yeah.

0:42:40.950 --> 0:42:41.390
**P4-Dr**
Yeah.

0:42:26.760 --> 0:42:51.240
**IK**
How could creative approaches be helpful in clinical practise, which you've already kind of touched upon with all your answers, but I think you also touched upon earlier on with how it it can, it might not be accessible to all. So how might creative approaches be unhelpful in clinical practise, so be it'll be great to hear your views on on both sides, you know, yeah.

0:42:49.790 --> 0:43:19.990
**P4-Dr**
Bye. Yeah. So obviously I look I think by nature of the course I run, I obviously think it is really useful for clinical care, because I think I've done a lot of reading around it and I've tried different approaches. I think these museum based trips are really nice just to get out of the clinical environment into a more levelling areas. It's it's unlikely most of anybody will have had a his degree in history of art and they might have. But even then applying clinical care to the arts, I don't think many people have got a degree in that so.

0:43:20.400 --> 0:43:51.480
**P4-Dr**
I think it's a very levelling thing and always saying the well-being aspect of just coming out of that very more kind of prescriptive stress inducing clinical environment, even if it's in a seminar room on campus, is really beneficial. And I think then doing things in galleries. I think there's lots of parallels you can learn about clinical observation from paintings, lots of research to support that which are in. There's also I've tried a few activities with reflection. So you know, picking a piece that makes you think about joy.

0:43:51.840 --> 0:44:22.560
**P4-Dr**
A quick piece that makes you feel so you know these, like, very nice, kind of hopefully nice prompts that the students can have a chance to think. Actually there's not going to be a wrong answer here is there. Like there isn't the right painting or the right and again that makes you think actually you everyone's opinion is valid. That's extremely important for your own self esteem. When you go into the clinical environment and then this complexity and ambiguity thing like sometimes we don't know the answer but we can understand one part of it and that could be enough or.

0:44:22.680 --> 0:44:47.600
**P4-Dr**
Understand any of it and we need to look at that again or ask for expertise or ask for help. Or do we need the answer? So I think that helps with clinical care there. I think there's a lot about working in teams and reflecting. So like we've tried some activities like doing poetry, poetry in galleries where everyone's gone. I don't like poetry or I can't write it. But then when you work as a team and you will write one line, then you can put together a poem.

0:44:49.40 --> 0:44:53.440
**P4-Dr**
And then, yeah, so I think like there is this and also reflecting with peers.

0:44:54.220 --> 0:45:23.780
**P4-Dr**
Going somewhere and giving everyone the permission to kind of not have to kind of like, I don't know, be on the rat race of, like, learning all the stuff for the exams, but actually the actual skills that might sustain your career, you can develop those because now when I I did my master's thesis on burnout in newly qualified dot GPS. And I think the thing they thought looking back was that when they went to those kind of training events where they meet with other trainees, the main thing they enjoyed was.

0:45:24.50 --> 0:45:52.970
**P4-Dr**
Little outings with their peers and it's kind of like the thing that people enjoy later on when you're working is catching up with your friend, usually in a kind of more creative environments like normally you don't catch up with your friend in the hospital canteen. It's like you get away from that and you catch up with your friends watching a film or chatting or, I don't know, you know, making like baking cakes or whatever you wanted to do or trying out new food in a restaurant and things like that. And it is more about.

0:45:54.130 --> 0:45:55.10
**P4-Dr**
Thinking actually.

0:45:55.220 --> 0:46:3.180
**P4-Dr**
Other skills that probably going to sustain you and prevent you from burnout later. So yeah, so that was I think you can get all of those probably million other things.

0:46:4.980 --> 0:46:8.580
**P4-Dr**
What might it was the other one, like what would be the pitfalls or?

0:46:7.900 --> 0:46:10.900
**IK**
How it could be unhelpful did if any.

0:46:9.740 --> 0:46:21.980
**P4-Dr**
How can you be unhelpful? So yeah, I do think that the arts generally has got bad press. And I think if we're from working with the artist that I work with on my project, I think they also agree.

0:46:23.340 --> 0:46:44.60
**P4-Dr**
That perhaps it hasn't reflected the diver like I think artists have like on the whole are very I mean I love the art. So I'm always really inspired by everything they create. I think sort of textbookly they haven't been the most diverse of cohorts or maybe the people that we meet in the public eye are not the most diverse.

0:46:44.560 --> 0:47:0.720
**P4-Dr**
And therefore, I think sometimes our students can't see themselves in that person. Like I talked a lot about my mentors or people that I've met through my career. But I think sometimes if, if if a student or people can't see themselves in that.

0:47:2.0 --> 0:47:14.640
**P4-Dr**
I think the way that we're sort of our education system is set up with our it's like kind of like if you it soon becomes apparent, doesn't it, who's good at art and who's not. And I think people have gone with that that label through.

0:47:15.100 --> 0:47:47.180
**P4-Dr**
The rest of their life, unfortunately. So I think sometimes people are like being told right. Do you know what you're really good at science and obviously you're going to be a great doctor, but you know your your heart was rubbish or whatever it was. So people think you can't do the two things together or they were like, oh, you know, so and so is not the most creative one in our family. But he went on to do science or she went on to do science. And she's like a fantastic doctor. But do you know, like it's not like normal for it to come together because there's a very seems like especially early years quickly. It's very defiant. Like I think of my son's age.

0:47:47.820 --> 0:47:51.900
**P4-Dr**
Which is like 3 and is very free like everyone can do it. It's like.

0:47:52.340 --> 0:48:17.340
**P4-Dr**
Optional. It's like we should all do it and then something happens where my older son's now, Sara. Nate, where they. They're still drawing to express or what they're doing creative activities to express. But people have started saying so and so seems really good at it. So in theory is really good. And then that label I think stays and then the way that we're set up, it's like the arts become like an add on or you pursue them, but you don't, we don't crossover for a while.

0:48:18.740 --> 0:48:22.460
**P4-Dr**
And I think that can be quite excluded. I think in a way.

0:48:22.940 --> 0:48:52.620
**P4-Dr**
And then I think diversity in what we I think galleries are trying to to, to change that now and it depends on which gallery you go to. But for example like I teach at the welcome as well as the Tate Modern and they're very different from when I went to the core child gallery in Somerset House where and also the Guild Hall where actually in a way it's a nice teaching experience where you're like, oh, none of the paintings or the art looks like anyone that I know or have met in my life.

0:48:53.690 --> 0:49:3.330
**P4-Dr**
And then it's quite hard to teach those clinical concepts, but you can teach. Actually, diversity is really important. So I've had those pitfalls.

0:49:5.10 --> 0:49:30.90
**P4-Dr**
I do think like there's a pressure, isn't there to come out as a doctor and if people don't, can't see the direct benefit and and I think it is hard if some people to see the direct benefit of the art in clinical care if they don't get that, if they can't understand it or they're not at the point that they're open for it, then it's really hard to force someone to do a clinical activity. But normally if there's it's a bit like behaviour changing.

0:49:31.150 --> 0:50:1.950
**P4-Dr**
If there's a seed of OK, I don't. I think this is going to be a bit rubbish, but I'm going to give it a go because everyone says it's good, like even that is usually enough to to kind of give make people have a go. But I think if you come with zero or you definitely come with like, I'm going to get nothing from this. This is a waste of time. It's going to take me away from doing what I want to do. Then you probably are going to get nothing from it. I think so. It's really sometimes it's hard and I think you can't again it's like.

0:50:2.430 --> 0:50:14.390
**P4-Dr**
Doing yards also makes you realise that that it may be the wrong timing for somebody, or it might be they're not ready or they're something else going in their life that makes them less open to that.

0:50:14.430 --> 0:50:32.510
**P4-Dr**
And I think that's OK, but it would be great if you could then revisit that and then that because I think that that is a reason why when we're younger, we all do do this as children, but we all do any element of play or creative like play is creativity really isn't it like so? And human beings need that. So I think.

0:50:34.110 --> 0:50:46.270
**P4-Dr**
I think yeah, it's just, but I think you can't force it because that's sometimes worse that you're like, you have to do that 'cause then people become feel unsafe and then they don't want to do it.

0:50:46.310 --> 0:50:51.550
**P4-Dr**
So they've got to be something that you can hook people in on. I think otherwise it's hard.

0:50:58.60 --> 0:50:58.180
**P4-Dr**
But.

0:50:59.750 --> 0:51:0.430
**P4-Dr**
Thank you.

0:50:52.510 --> 0:51:1.630
**IK**
Yeah. Thank you. And it was really interesting your views and your points. So thank you. But now to to.

0:51:3.40 --> 0:51:10.320
**IK**
Kind of end. If there's anything you'd like to add or or share you know regarding the role of creativity in tolerating uncertainty.

0:51:21.140 --> 0:51:21.260
**P4-Dr**
Hmm.

0:51:11.780 --> 0:51:27.300
**IK**
And if there's possibly like, I don't know, a question that I probably didn't pose that you thought, OK, I could have answered this or I wanted to say this, but I wasn't given the space to do so. So if there's anything like that, that would be great.

0:51:27.50 --> 0:51:51.90
**P4-Dr**
Yeah. No, I think your questions were quite, I think I think they are something that perhaps like you said, when you're asking why it is interesting to actually really stop you and make you think why? Because I think if you're naturally bought into it, you feel like your brains already gone into that and you know, you think it's helping, but actually like unpicking that to find out why is is really interesting.

0:51:53.680 --> 0:52:26.800
**P4-Dr**
And I'm not sure which one came first. I think you know. Do you know? I don't know where. Naturally some of the people that you're talking to are probably going to be already have made that conversion, whereas in a way, I think it would be really interesting to talk to people who who, who aren't sure and haven't already made their mind up. And I wonder if it must be quite hard for you to recruit those kind of people because they naturally are not self selected into doing a study like this or have you been able to kind of just sample like the average student that you know was like kind of you don't know which feeling they.

0:52:27.600 --> 0:52:28.520
**P4-Dr**
Which way they might go?

0:52:35.950 --> 0:52:36.350
**P4-Dr**
Yeah.

0:52:28.810 --> 0:52:37.410
**IK**
Yes. So I haven't, I haven't done that too, but I think that was it would be something to really think about afterwards. It'll be interesting, but for now it's I think.

0:52:41.30 --> 0:52:42.990
**P4-Dr**
Who've done it? Who's who's here?

0:52:43.640 --> 0:52:44.120
**P4-Dr**
Yeah.

0:52:38.850 --> 0:52:47.410
**IK**
Is, I think, easier to ask people who've done it or people who are in certain groups. So like people at the like, all of the educators at the conference or.

0:52:48.30 --> 0:52:48.430
**P4-Dr**
Yeah.

0:52:49.50 --> 0:52:51.370
**IK**
The students at at the Barts, so.

0:52:55.820 --> 0:52:56.260
**P4-Dr**
Yeah.

0:52:52.870 --> 0:52:57.910
**IK**
Then it's I think easier also for ethics as well. Compared to me just saying that I'm going to interview.

0:52:59.200 --> 0:53:3.760
**IK**
People who haven't done it and then they'll ask how and those kind of questions.

0:53:5.50 --> 0:53:8.90
**IK**
Yeah, but I think it's really interesting, yeah.

0:53:3.70 --> 0:53:33.230
**P4-Dr**
Yeah. Yeah. Why are you doing that and stuff? Yeah. No, exactly. No, I just. What I'm thinking as we speak 'cause I 'cause, I think as we're speaking, I'm sort of more like, I wonder which one I am actually thinking. Which one converted me for like was it the fact I naturally was open to the art meant that I was able to do, you know, deal with uncertainty or was it like I the other way around? Like do you know like or was it the? And then I was able to deal with the arts or was it the other way around, but I think.

0:53:33.750 --> 0:53:40.70
**P4-Dr**
It is something I think you're right. Like it probably not that you're right. See, I don't think you pushed me into saying, but I think it's right that.

0:53:41.310 --> 0:53:47.470
**P4-Dr**
Doing those activities throughout my professional career and not just stopping at GCSE and never doing that like.

0:53:49.510 --> 0:54:5.950
**P4-Dr**
It kind of, it has kind of brought like a flexibility to my mind, I think and an openness to try out things which are outside the the box. And I think I've always had that kind of feeling. So no, I don't think I've got any other questions. I was just thinking aloud about.

0:54:6.660 --> 0:54:8.740
**P4-Dr**
I wonder what other people are saying and things.

0:54:9.430 --> 0:54:16.990
**IK**
Yeah. No, thank you. So I, I'll, I'll stay on, but I said, OK, if I just stop there, the recording, if that's OK with you.

0:54:16.790 --> 0:54:23.550
**P4-Dr**
Oh yeah, sure, sure. No, I think I'm. I'm very good at talking. So I could probably talk for three hours and you wouldn't be able to kind of keep up so.

0:54:23.850 --> 0:54:24.450
**P4-Dr**
Perfect.
