# Supplementary material for: How does creative enquiry enable us to deal with uncertainty?
Source: BMC Med Educ. 2026 Aug 8;26:1785. doi: 10.1186/s12909-026-10060-w (PMC13732821; doi:10.1186/s12909-026-10060-w)
Supplement: Supplementary file 8 [file 12909_2026_10060_MOESM5_ESM.docx]

**IK**
What does creativity mean to you as a clinician?

**P5-Dr**
Two parts to that question isn't there so.

What does creativity mean to me? And then as a clinician, because and that's an interesting phrasing of the question because I suppose as a human being, creativity means something which is new and responsive and original. It often has a medium, so it might be the spoken words or something that is transcribed or potentially artistic. And I guess it's often responsive to an internal experience, which, maybe triggered by experiences in the world.

**P5-Dr**
I guess if you ask as a clinician, that's I guess slightly changes my answer because it makes me start to think that I have to have maybe a special type of creativity that's got something to do with being a doctor as well. At the same time that gives it a slightly different context.

**IK**
Yeah. So I don't know. It might also be as a medical student or through your medical career. Have you seen creativity and if so, how would you kind of describe that or categorise that as being creative in the medical sphere?

**P5-Dr**
I think there's a certain element of creativity within the job itself. Often when it comes to more difficult parts of the job, like with communicating with patients. Potentially having difficult conversations about DNACPR or breaking bad news about a cancer diagnosis or to be honest, in any form of communication one can be creative. It can be an opportunity to create in a job that is very uncreative where there are so many guidelines that there are these parts of the job where you get to inhabit a more creative space. So, it's something that's a lot more intuitive than your day-to-day algorithm generated medicine. So you can be creative, responsive to the person sitting in front of you and trying to be creative with your communication and how you reassure them. You try and give someone what they need and try and be authentic and honest from your own point of view as well. And then there's creativity more in the sense of processing difficult experiences and reflecting experiences as a practitioner. For me that has always been in the form of writing. After difficult experiences, writing and the vast majority of the time writing in a personal sense to make sense of what's happened. To process what's happened. Also an extension to that, it can be used in the form of activism as well. So I've used creativity a while a while ago to take that emotional response and write articles about experiences in order to bring attention and life to a problem.

**IK**
Yeah. And it's very interesting. You talked about the activism. People really don't talk about what you do with the creativity. So you were just kind of saying now this idea of writing articles on your experiences. So how is that being creative? Is it because you're talking about your experience, and that alone is being creative, or is it because you're processing it? I don't know.

**P5-Dr**
Yes, it's a good thought. I mean it's both, isn't it? So I think on the one hand, the creativity let's say you know…I'll give an example. So it was a long time ago when I was a junior doctor. I was working in a low income country doing some humanitarian aid work and seeing some very challenging things. So it's managing children with severe malnutrition and I would go home and try to make sense of it. And that was a personal thing. So that was a personal thing to process it and in the end, lots of things happened. But anyway, one part of what happened was I wrote an article for a newspaper. It was the intention to be an activist, to shed light on the issue. But then it's also a response. It's a therapeutic response for me. It's like, “Oh, what can I do about the situation?” And so, it's a way of turning a subjective experience not just into something that's reflective, but into *something*. The act of doing is actually makes you feel better because it's like a response to a problem in the world.

**IK**
Yes. And I think you touched upon earlier of creativity as this kind of responsiveness. Using an experience that you've had and that kind of triggers you to an emotional response or doing a form of art or writing in what you were saying. So do you think creativity means you have to you need a past experience in order to be creative?

**P5-Dr**
I'd say two things. So I think that on the one hand, just by the very nature of existing, we all are experienced in this mystery. This mysterious kind of condition, of consciousness and life. So I mean, you can make creative, which may just be a response to the human experience. Just that everyday mundane human experience, I think on the other hand, at the age of 21 as a medical student, you may have held someone's hand when they're dying. You've definitely seen a dead body. You've definitely seen a death, you may have delivered a baby. A patient may have told you their deepest, darkest secrets, which they've never told you know their husband or wife, for sort of 40 years. As a medical student, as a doctor from a very early stage in your adulthood, you're exposed to incredibly deep human experiences. And you have your own emotional response to that. And so I think there is a unique profundity. Extreme human experiences that you're exposed to and you're part of. It's a unique position compared to a friend who's doing accountancy or something like that. You know, it's such an early stage in one’s development. So the material there to create, to fuel an emotional turmoil, but also be creative. Like a lotus flower growing out of that chaotic garbage it's quite significant I think.

**IK**
Thank you. And now you've already mentioned you're writing, as a junior doctor, but you could talk about really an example where you've used creativity to help you reflect on your past experiences in clinical practice?

**P5-Dr**
Developing a writing practice is very helpful. Sitting down immediately after, or maybe the day after something that was been quite affecting, potentially a traumatic experience or traumatic event and just putting things to paper is quite therapeutic. That's what stories are like. We live our lives and we make sense of our lives through stories. And that's why stories are so appealing to us. And I think sometimes by writing and giving something a narrative structure that is an act of making sense of what's happened. Then looking back on that, like days, weeks, I mean in my case even eight years later, looking back at particularly traumatic events that I've written about that is quite therapeutic. And it's just helpful. It's helpful because I think often these very affecting and traumatic events, especially earlier in our careers and earlier in our lives, can define our values and things that matter to us. And I think it's quite difficult to think of these things in the abstract, but when one sits down and looks back at a series of creative or written reflections, looking back it can start to make sense of your life. I've used it in deciding how I want to take my career next. Looking back at traumatic or difficult events that have shaped me has been very helpful recently in deciding about making some potential career changes.

**IK**
Yeah. And I think you just touched on this idea of making sense. So, how does the actual piece of writing or the process of writing make you make sense of it?

**P5-Dr**
It's a good question. Writing is a fully reflective exercise. So, I think when you're thinking or ruminating you can only really have like half or one sentence at a time and that one sentence just kind of loops and cycles and loops and cycles and then it might become quite painful. So you just push it away. When you're talking to a friend, that can be very helpful. But when you're talking to a friend, there's always the tendency to make something entertaining or not burdensome and hold back a little bit from the actual sheer horror of the experience. The difference of writing is that you get the first thought out and then it's on the paper. So it's out of the short term memory and that gives a bit of space in the mind for the next thought to come out and then that could go down onto the page and then that gives space in the mind for the next. One thing you can just do is vomit each passing thought onto the paper and that in itself is just kind of getting it out of the system. But then there is something in the crafting that, you know, the crafting of the words. The self reflective human experience makes sense of the world through a limited variety of dramatic archetypes. I think by taking one's experience and turning it into a story helps bring structure to that experience. That always has the danger of trying to think of a better word but obscuring some of the truth or authenticity of the experience, because often we like to bend narratives to that particular structure. I think storifying things, you know, making things into narratives is quite fundamental to who we are as human beings, and so it fundamental to making sense of experience.

**IK**
Yes, thank you. And now looking at the other side of the interview. So do you mind if you could talk through a time which was uncertain?

**P5-Dr**
Interesting. I don't want to give you anything too traumatic because I don't want to burden you with all these experiences. There’s one thing that really comes to mind. And the reason is because I was looking through some of the things and some of my notes recently that I'd made, so it’s slightly fresh in my mind. It goes back to this time when I was working in this hospital doing some humanitarian work and I had just assisted in a caesarean section and it was quite a sort of obstructive labour. so the. The baby was in quite a bad condition when it came out and we handed the baby to the midwife and she started suturing the mum and the baby was taken away. I think the mum was under a general anaesthetic so she was quite sleepy the mum got taken back to the ward and I went out of the hospital to write up the operation note about the caesarean section. And I sort of look down. I was sort of writing a note and on this bench I remember it being a long, cold, dark corridor and it was about 2:00 o'clock in the morning and there was this little pile of blankets next to me. I thought, “OK, it's just a pile of blankets”. And then the pile of blankets started moving. And you know, it's just me and this bench and I was writing these notes completely. And then I looked down and I realised it was the baby.

And I picked up this… This baby's been left alone on this wooden bench outside the operating theatre and the baby was sort of breathing about once every 15 to 20 seconds, you know. So it was not doing well at all.

The baby had been attempted to have been resuscitated, I guess by the midwife. And at this point, the baby was now taking its last breaths. Although this was quite a common occurrence in these obstructive labours, it was just really, really shocking, you know, just to kind of be left alone in this cold corridor with just this baby that was dying and I just held this baby. I had no idea what to do. And then eventually, the midwife came and she gave the baby back to the mum. Just to spend the final moments together.

This was just like a really, profoundly moving and shocking experience and one of those things that no one tells you. What do you know? Like no one kind of helps you make sense of that in your medical training. And so, yeah, one thing I did do was obviously make sure that the baby was taken back to the mum.

But yeah, once all that was done, I can't remember. I probably went home and cried.

And then the next day, I don't know exactly how long later, I just wrote about it.

And, Yeah. There you go. There's a traumatic experience for you.

**IK**
Is the limited resources also the reason why the baby was in that situation or as you were saying kind of a common occurrence?

**P5-Dr**
Yeah, the infant mortality rate is quite high and there's quite a lot of obstructed labour.

People often labour up to 24 hours before seeking help, so by the time the baby comes to hospital often they’re not in a good condition and it's got some hypoxic brain injury or it is dead and you know C section is done to save the mother's life, but not the babies.

**IK**
Yes. And I think you said that after you wanted to write. Did it help you process that? And was this what led you in taking a more outward in trying to make this clear with other people. Or was this just an event that you've reflected on for yourself?

**P5-Dr**
It's a good question. Thinking back, it was a while ago. Again writing. It was a relief. It's a relief to put the words down to kind of share the experience, even if it's just with the screen. I do most of my writing on the screen. So yeah, and in terms of activism, there were experiences not dissimilar from that made me want to write articles and shed light on some of the injustices that I've seen. So absolutely.

I think on the other hand, one does sometimes feel that some experiences, you know, there's not really anything to be gained. It's just a really bad thing that happened and I'm not sure how sharing that with other people would necessarily help. And also people don't want to hear some things that are quite traumatic and people don't necessarily want to hear it.

I think with that information I just buried it. You know, I just wrote it down, but it was shared. It was shared with myself and it was buried down somewhere.

**P5-Dr**
And again, talking about the reflection stuff. You can come back to that few days later and sort of relive the experience, but it's now outside of you. It's not a ruminating thing because it's committed to that piece of paper. I've looked at that recently and, you know, years later. But even looking at things years later, it's very therapeutic and it's very, very helpful and you know, helps one forgive oneself. Reminds one of things, that one's been through, but at the same time it helps one in terms of forgiveness. You can let things go but I still think now, “Did I do the right thing? What should I have done?” Maybe I should have restarted resuscitation at that moment. But I think there was a part of me that just knew that how long the labour that they'd already attempted resuscitation and that the baby was too far gone.

**IK**
How did the writing make you deal with the experience?

**P5-Dr**
Thinking back to that moment or that time, there's something about writing when something's fresh in one's mind. I find my memory is not great. When you look back to experiences it could be very much biased by the mood one’s in when one's looking back. When you write something down, it's quite real and it's quite honest, but it's fresh in your mind. I think as I was saying before that through the process of writing one can get multiple thoughts out, instead of just having one dominant thought looping through one's brain like, “Oh, I'm not a very good doctor because that baby died”. One gets to kind of put out the whole story.

Looking back from eight years ago and having something external on that piece of paper, it's externalised and I think that's easier to respond to. It gives you space, doesn't it? Because it's not something inside that you can't get away from. It's something external to you. It just makes it less scary. It's written down on a piece of paper compared to when something is rattling around in your brain.

**IK**
Yeah, thank you. Now, how do you typically approach uncertainty?

**P5-Dr**
Hmm. How do I approach a certain situations? I guess it's different professionally and personally. If we're talking professionally, I find it quite easy now. In some ways to approach uncertainty, you have to make a decision with the information you have available to you. I'd like to involve patients in the uncertainty as well. It's applicating a bit of responsibility, but I think that's important to say. Look, I don't think it's this. I don't think it's that, but it could be. So we've done the test. We've pretty much ruled it out, but you know if things change and you're not getting better or whatever, you have to come back. You know, I don't think you've got a blood clot, but you know, I'll say I'm, you know, so I like to involve patients. I think patients hate arrogance and they don't mind people who are trying their best for sure. So I think showing them your thought processes and involving them in these decisions is probably a best way to do it.

**IK**
Your approach is kind of involving patients or embracing the uncertainty with what the outcome might be or what treatment to take. So do you think that's come about because of your writing or because of your creativity or was that something that you've already had?

**P5-Dr**
I don't know. I don't know. I think it's a lot of things.

**IK**
So there's a statement I'm just going to read out. So in a scoping review, there have been multiple studies that have shown that creativity can enhance the tolerance of uncertainty. So what are your views on this and why?

0:40:7.205 --> 0:40:11.485
**P5-Dr**
Creativity can enhance tolerance of uncertainty. Interesting. What are my views on that one? Sounds good. I like it. It makes me want to go and do more creative work now because, you know, if you tell me that it’s going to help me with my mental health, you know, it will help me cope with uncertainty.

0:40:47.550 --> 0:40:48.630
**P5-Dr**
There is a distinction between the things that we do that are sort of evidence-based, guideline-based following algorithms etc. And the things that you know often, we're making decisions outside of that. I think in a way that medical learning is very algorithmic. And I think the other stuff is just not taught. Yeah. And it strikes me that the act of creativity by definition, is not an algorithmic thing . By being creative, one does have to harness uncertainty.

0:41:52.790 --> 0:41:56.230
**P5-Dr**
That's not creative. And so I wonder if it's.

0:41:57.520 --> 0:41:58.520
**P5-Dr**
That's a kind of.

0:42:3.270 --> 0:42:9.590
**P5-Dr**
You know, exercise is a similar part of the brain. You know, the part of the brain that's.

0:42:9.630 --> 0:42:15.790
**P5-Dr**
Create. You know, creativity is an actual uncertainty. So yeah, I wonder if by being creative.

0:42:17.430 --> 0:42:21.830
**P5-Dr**
One inhabits a space necessarily, and habits and becomes comfortable.

0:42:23.450 --> 0:42:25.370
**P5-Dr**
Within a zone of uncertainty and so.

0:42:27.430 --> 0:42:36.110
**P5-Dr**
It's a very analogue, sorry. Yeah, very analogue space. It's not like a digital, you know, algorithmic space. It's a space of kind of shades.

0:42:39.60 --> 0:42:48.420
**P5-Dr**
And it's a space where there isn't always. You know, you can't necessarily be right or wrong with creativity. And so again, there's a there's a, there's a, there's a parallel there with.

0:42:51.150 --> 0:42:56.790
**P5-Dr**
With uncertainty isn't that, you know, like uncertainty is a place where things are not necessarily right or wrong.

**IK**
If creativity is uncertain, then why use that to deal with something that is uncertain?

0:43:17.780 --> 0:43:24.780

**P5-Dr**
It's a good question. Well, I think like, I mean that there's a, there's a maybe a.

0:43:42.380 --> 0:43:45.820
**P5-Dr**
A something to say to that which is.

0:43:47.700 --> 0:43:58.460
**P5-Dr**
Often the certainty that we we look for in medicine is in some way imposed by us, you know, and it's somewhat arbitrary. You know, we say, ah, you know, if.

0:43:59.840 --> 0:44:0.120
**P5-Dr**
You know.

0:44:4.170 --> 0:44:4.370
**P5-Dr**
If.

0:44:5.620 --> 0:44:24.620
**P5-Dr**
Test number is about this threshold. Then the patient will benefit from from this medicine. But you also know that like actually you know that's a very you know that's a very it's gonna that's actually very it feels like a black and white world but it's not you know it's a great world and I love the quote that I can't believe who said it but.

0:44:27.140 --> 0:44:48.100
**P5-Dr**
You know, at least 50% of what we know in science right now is wrong. The problem is we don't know which 50% that is, and I think that's so true. Not not, not to disparage science because a lot of you know, like, you know, things that, you know, a lot of things that's good in the world has come from science. But I think to pretend that.

0:44:50.720 --> 0:44:57.720
**P5-Dr**
The things that we know in, in, in inverted commas, you know that we actually know them is is quite.

0:44:59.400 --> 0:45:17.400
**P5-Dr**
Is quite false, and so certainty in some ways the guidelines you know that we follow is a way of imposing certainty in the world that helps us relax and feel better. But the more one does, the more one understands how these things are put together and the more one sees and experiences, one starts to realise actually very, very uncertain.

0:45:18.140 --> 0:45:24.940
**P5-Dr**
Even within the confines of the guidelines. So yeah, I wonder whether with creative acts or maybe just.

0:45:26.560 --> 0:45:46.560
**P5-Dr**
Experience or I don't know. I do a lot of meditation, but that is just learning to be OK with things the way they are. And so, you know, it's kind of how the world is. The world is a very uncertain place and if one tries to fight that and makes it more certain, but it isn't, then that's potentially quite.

0:45:48.40 --> 0:45:48.80
**P5-Dr**
A.

0:45:48.560 --> 0:45:57.360
**P5-Dr**
Inauthentic thing to do, but when one learns to just kind of, yeah, you know things are difficult. Things are uncertain.

0:46:2.980 --> 0:46:7.300
**P5-Dr**
You're not fighting against reality, then, are you? You'll just accepting things as they are.

You're tolerating it.

0:46:8.650 --> 0:46:21.210
**IK**
It's the creativity kind of does that as well that that like the meditation does the creativity have that same characteristic of kind of acceptance and embracing, or are they different?

0:46:19.930 --> 0:46:26.650
**P5-Dr**
I think so. Well, I think it's sitting. It's sitting with things, allowing them to be and allowing them to unfold.

0:46:29.690 --> 0:46:32.10
**P5-Dr**
Because in some ways I mean it's a bit of a mystery, isn't it?

0:46:32.940 --> 0:46:35.580
**P5-Dr**
Actually, where creativity comes from, it's something that's.

0:46:37.80 --> 0:46:40.520
**P5-Dr**
You know, if one is.

0:46:42.170 --> 0:46:43.290
**P5-Dr**
Consciously.

0:46:45.130 --> 0:46:47.170
**P5-Dr**
Top down, directing it then.

0:46:48.670 --> 0:46:48.710
**P5-Dr**
It.

0:46:49.180 --> 0:46:55.260
**P5-Dr**
But it kind of fails to be, but stops being creative, doesn't it? It's something which.

0:47:22.430 --> 0:47:22.710
**IK**
Yeah.

0:46:57.510 --> 0:47:26.990
**P5-Dr**
Unfolds almost autonomously, and then the conscious part of you kind of can shape and control that. But it's that's, you know, it's something that is in its essence, quite mysterious. So I think it is something that is quite uncertain, you know, and and we we tame it with our skills and our crafts. But yeah, I don't know. It's just sort of a thought, but yeah, potentially sitting in the creative act is quite an uncomfortable and may be the source of a practise where one increases one's tolerance for uncertainty, but again, that's a thought. I don't know. I'm not sure I I won't claim certainty there.

**IK**
Do you have any examples where creative approaches have been helpful, or do you think they can be helpful in clinical practise and the flip side of?

Do you think it could be unhelpful?

**P5-Dr**
Sort of reflective writing.

0:49:41.980 --> 0:49:45.900
**P5-Dr**
So that's I think I think that is very helpful. That's very helpful for healthcare staff, clinicians to process and make sense of their experiences.

0:50:3.30 --> 0:50:9.950
**P5-Dr**
I don't work in an area that allows a lot of space for maybe exploring things like that.

0:50:11.390 --> 0:50:31.870
**P5-Dr**
Or elaboratively or with patients. It's just. Unfortunately, it's not something it might be different if you're doing something like palliative care or in AGP practise, I don't know, but it's just not, unfortunately, in acute hospital setting, it's not really something that much experience with kind of trying to share with patients.

0:50:33.500 --> 0:50:43.180
**P5-Dr**
I think it it on the first point, if I think it becomes pointless and potentially counterproductive when.

0:50:45.970 --> 0:50:47.810
**P5-Dr**
It just becomes another tick box.

0:50:48.970 --> 0:51:6.450
**P5-Dr**
You know, you probably felt this or you probably come across peers when it's just like, oh, God, we have to go and do something creative now. Oh, goodness sake. You know, like, I have to do my and we actually. I mean, we just have to, like reflection, you know, even if you're doing something really technical in medicine, like hospital medicine.

0:51:8.30 --> 0:51:15.670
**P5-Dr**
We have reflective practise so it was like almost everything. You know you do, you have to put a little reflection in there and it just.

0:51:16.740 --> 0:51:20.180
**P5-Dr**
It becomes this hoop that people have to jump through and people.

0:51:22.880 --> 0:51:29.760
**P5-Dr**
Just pay lip service to it and it and it drives a bit of cynicism and I think it wastes it. Can't waste time.

0:51:31.300 --> 0:51:33.740
**P5-Dr**
So I think no. I think when reflection.

0:51:35.830 --> 0:51:59.710
**P5-Dr**
Like if it doesn't feel helpful then you haven't reflected, you know you you you've, you've you've tricked yourself, you know that's the thing about reflection. RP. Reflection is rubbish. I've I'll just do all this like reflection, inverted comments. It's like well, if that's not reflective then is it, you know like if it if it was useful then then you know if it was actually true reflective reflective practise then it would be useful. So I think that's a danger.

0:52:2.550 --> 0:52:9.230
**P5-Dr**
But no, I don't think you're gonna get many health care staff that comes to their training and just say, oh, you know.

0:52:11.960 --> 0:52:20.600
**P5-Dr**
I didn't learn how to calculate because I spent too long, you know, playing the trombone or whatever it is, you know, you know, I don't think.

0:52:22.120 --> 0:52:25.600
**P5-Dr**
You know, personally that we're in any danger soon of like.

0:52:27.270 --> 0:52:30.190
**P5-Dr**
Filling the curriculum and syllabuses with too much of this stuff.

0:52:31.800 --> 0:52:50.480
**P5-Dr**
So that's the main danger that I see, that it drives cynicism and it becomes a tick box culture. And then it just gets Co opted by the BLOB of, you know, medical training. Oh, we've got to get all the people to reflect. So they've got to do something creative because then as we're saying, you know, that becomes not creativity, doesn't it?

0:52:51.500 --> 0:52:53.300
**P5-Dr**
That's just following the algorithms again.

0:52:55.710 --> 0:52:56.310
**IK**
Yes.

0:52:55.710 --> 0:52:59.110
**P5-Dr**
But I mean, yeah, I mean always I guess what also has to be wary of.

0:53:0.390 --> 0:53:5.950
**P5-Dr**
You know, asking people to open the box and then not being prepared.

0:53:7.180 --> 0:53:12.780
**P5-Dr**
I'm not being able to deal with what happens when all the darkness starts to spill out.

0:53:12.820 --> 0:53:14.940
**P5-Dr**
So I guess that's the other. That's the other risk, isn't it?

0:53:15.820 --> 0:53:22.60
**IK**
Yeah. So kind of it also gain too much in one way, but.

0:53:22.780 --> 0:53:27.100
**P5-Dr**
Well, there's the hole then too. You know, in in disasters.

0:53:28.420 --> 0:53:43.300
**P5-Dr**
Yes, mental. I think it's like psychological first aid. So again, that's kind of some training that I've done. So you know people that have been through, you know, something like a refugee crisis or or something like that, you know, psychological first aid is.

0:53:44.740 --> 0:53:46.860
**P5-Dr**
Essentially, the people that want to talk about.

0:53:48.480 --> 0:54:17.960
**P5-Dr**
The bad things that happened, you give them space to talk about it. The people that don't want to talk about the bad things that happen, you allow them not to talk about it. You know, you don't force everyone to, you know, there I think there is something in sort of modern pop psychology that like if you've got problems, the best thing to do is is kind of like have those problems explored and you know, in, in sort of you know degrees and you know like to find granular detail. But I I think.

0:54:18.340 --> 0:54:32.780
**P5-Dr**
Yeah. So I guess that's a potential danger in medical practise, is that you tell everyone I was really good to get all your feelings out. And yeah, I mean, for some people, those things are so difficult to deal with. Their lockdown for a reason. So you got to be a bit careful.

0:54:34.680 --> 0:54:47.80
**IK**
So this idea that you don't want it to become mandatory or as you say tick box but there was a key thing I think you were saying about the difference between reflective writing and.

0:54:48.330 --> 0:54:54.810
**IK**
Kind of written portfolios or or things to submit for certain tasks to complete so.

0:54:56.150 --> 0:54:59.830
**IK**
What makes the reflection useful in your in your mind?

0:55:0.460 --> 0:55:4.660
**P5-Dr**
Well, again, I mean, yeah, and that's the other thing, isn't it? You know, there's there's the example of.

0:55:6.270 --> 0:55:36.190
**P5-Dr**
I can't there. There was some medical trainee who wrote some reflective piece. It's probably quite famous now in their portfolio and it was about how they've made a mistake. And then at some later point this thing was used in evidence against them, you know, in some form of training review. I don't know if it was agmc think or whether it was just a training review that someone, you know reflected truly reflected and and was quite honest and frank. And then that was that was turned around against them.

0:55:37.110 --> 0:55:41.150
**P5-Dr**
Yeah, I guess it has to be done within the confines of.

0:55:44.60 --> 0:55:46.780
**P5-Dr**
If there's if some degree of.

0:55:48.190 --> 0:55:51.950
**P5-Dr**
A safe space safe spacing as much as that's possible.

0:55:53.240 --> 0:55:54.800
**IK**
Yes, thank you.

0:55:55.940 --> 0:56:9.700
**IK**
And I think there was loads of interesting points and viewpoints that you've you've shared. So thank you very much. But really just to ask you if there's anything else you'd like to add or or you know share?

0:56:11.20 --> 0:56:22.460
**IK**
Regarding the role of creativity in tolerating uncertainty in clinical practise, well, if there was like I don't know, a question I didn't ask, I didn't pose that you wanted to say or share.

**IK**
Or anything else really that you you wanted to add?

**P5-Dr**
Thank you for the free therapy.
