# Supplementary material for: How does creative enquiry enable us to deal with uncertainty?
Source: BMC Med Educ. 2026 Aug 8;26:1785. doi: 10.1186/s12909-026-10060-w (PMC13732821; doi:10.1186/s12909-026-10060-w)
Supplement: Supplementary file 10 [file 12909_2026_10060_MOESM7_ESM.docx]

**IK**

First off, what does creativity mean to you as a, as a medical student?

**P1**

Creativity. Just an expression of oneself, thoughts and beliefs in any manner in which comes to me. For me, the creativity comes through like art and drama and music. So using my body or using not necessarily my words.

**IK**

So you said ‘anything that comes to you’, could you tell me more about what you mean by ‘when it comes to you’?

**P1**

Oh, I guess I have to be like, my inspiration has to be sparked. So whether it's a conversation with someone or it's something I see in the street or in a movie that I've watched, I'll have a certain thought or view that I wish to express. To other people, or even to myself, sometimes just for self-expression.

**IK**

So is it something that comes like out of the moment, or is it something you've always thought about in the past?

**P1**

I think it depends. I think it's more in the moment, but sometimes I'll find myself thinking or pondering about a certain thing for a while and then deciding to be creative with it. And some things I'll try and express through words or through a conversation with someone, and it doesn't exactly work in the way I wish it to come across, so I'll use a creative means to help better both my understanding and then also other people's understanding.

**IK**

OK. Thank you. So moving on from the idea of creativity, as you said, expressing one's thoughts and through kind of those different art forms, could you talk about an example where you've used creativity in the past? And just reflecting on that and how that might have helped you during medical school or during clinical practice and after that really. So during the use of creativity and also afterwards.

**P1**

I mean the only thing that really springs to mind is my creative inquiry that I did last year. Can I talk about that?

**IK**

Yeah, of course. Yeah, whatever you'd like to do.

**P1**

OK, so I did. I did an art piece such as the drawing on based on a conversation I had within the creative enquiry course and the conversation was about flourishing. And then as I was interested and reading about flourishing, I came across the idea of the third space. And because the third space is very ethereal. Like it's not a thing you can't like, there's no way of me. Like I can't even describe it now using my words. And then I felt that while I was trying to reflect on the conversation that I wasn't finding the words to describe what I'd read. So I decided to put it into an art form, which was the drawing. And then through the drawing, I was able to find the words in which I wish to use to express myself.

**IK**

OK, and you just said something about flourishing. So with the idea of flourishing, you were talking about this. You were doing it during your creative arts SSC. So could you tell me more about what you do in the creative arts SSC or how you use your creativity there?

**P1**

Yeah. So the creative Arts SSC is a really good programme that Barts do. It's a self selected component. So it was two weeks off timetable and we were with Louise who ran it and she brought in different people each day to show us a new aspect or a new way of expressing ourselves creatively so. A notable session would be like our collaging session, so we literally just did 3 hours of collaging. And it was very therapeutic. I think medical school well, I know medical school is very stressful and a lot of people don't find the time to, you know, sit down with themselves and their thoughts and just use their hands or be creative for a bit. So I think that space was really lovely. Like, she created that over the two weeks. Like, I wasn't thinking about my work. I wasn't thinking about how stressed I was or the difficulty of moving from secondary school to university or like what I was going to cook in the evening. I just had the time to be creative. So we did things like photography, we did a drama workshop, collaging as I said, and also even when we were like discussing things with Louise, she would always let us like, doodle on a pad of paper or something which just let our creative minds flow. And it was just nice. It was also really nice to see other people who were creative in different ways than I was. So there was a lot of discussion around how different people use their creativity and how conversations spark different like inspirations in different people, even though we've had the same conversation. We can create different pieces of art or be creative in different ways from it. And I think that was the most interesting thing.

**IK**

OK. Thank you. So just to summarise like what you were saying. One key thing you said from the creative arts course, was it allowed you to have a break from what you do normally at medical school with all the stresses of exams. Did you say exams?

**P1**

Yeah. Exams and stuff.

**IK**

And then you also said it allowed you to have that flow. So I'd like to know more about that. But also you said it allowed you to have different conversations. So you had one piece but when you did it or your friends were doing or I think different people, you could all have different takes on it, essentially. So could you just tell me a little bit more about… you could take it one at a time or I could remind you as you go?

**P1**

OK. I think the main thing about medical school is that you're constant. I mean university in general, but obviously I only have the medical student standpoint is that you're constantly working and there's constantly things to be doing. And for someone who is creative, I like the idea of being able to go home and draw something or take some photos during your day. But I think there's a lot of guilt placed in taking time for ourselves, especially as medical students that we place on ourselves. Like no one else places that pressure on us. But I felt like as I was starting the creative arts SSC and I was like, why don't I draw more? Why don't I take photographs of things that I find pretty and why don't I do these things that I enjoy? But it's because we never find the time to just sit down for ourselves and do something creative for ourself. So I think the ‘flow’ was just being allowed that time. Because it was on our timetable, there was no guilt associated with being creative because I was still doing something for the university. But it was like Louise was giving us the space to just do what we wanted to do and be creative in our own way. And then the discussions, I mean the most beautiful thing about art is that everyone has completely different takeaways from it. Like everyone looks at a piece of art differently. So I mean you can relate it to your medical school life. Like everyone's going to take something different from a conversation or from a consultation. So being able to see different people's points of views, it just opens up your mind a little bit to be like, yeah, you have one view which isn't necessarily right or wrong, but then other people will see the same image with different things. And that depends on what they've been through or conversations they've had or even just if they've had a conversation that morning that's put them in a particularly different mood, it could have an impact on how they see things for the rest of the day. So I think it's just using that knowledge and then being able to relate that into our medical practice, so that everyone we speak to and everyone we try and treat or help is going through different things and will view a situation differently. And it's just being open minded and inquisitive and eager to see things from different people's points of view.

**IK**

OK. Thank you. And I'll move on to the next question. Talking about uncertainty now, so could you describe the time when you've had to face a lot of uncertainty. I might be during medical school or in clinical placements or in clinical practice or anywhere in that medical background. Have you seen or have you experienced a lot of uncertainty and could you just tell me more about that if that's possible?

**P1**

Umm, I don't think I could pinpoint it to a specific thing. I think the whole concept of going to university and going to med school is full of uncertainty. I think the fact that coming to university, I was like, everything's happening all at once. Like you're moving out of your home, you're suddenly having to cook for yourself. You're having to be on your own structure. At university, all the work is very self dependent. There's no one reminding you to hand-in an essay and then going on wards, it's up to you to make the most out of it. And then a lot of us, as medical students who kind of feel like not a waste of space on the wards, but like, what am I doing here? Like I can't really do anything yet. Our course is 5 years. I just think the whole process of growing up and also shaping ourselves into doctors and we're also trying to keep on top of a work life balance. It's just the whole thing is full of uncertainty. Sorry, I don't know if that's…

**IK**

No, no, no, I think that that's very… I think you said a lot. So one thing you were saying is this idea of transformation in that sense. So tell me if I'm not summarising in a way that makes sense or explains your view, but if I'm right, you were kind of talking about this idea that from secondary school to med school, it's been a transformation. And I think you were saying independence. So you're saying like you had to do the cooking, you had to look after the flat. There's rent, things like that. And then you talked about these new environments at clinical placements. How were they uncertain? Is it because they were new to you or were they all so fast-paced or you've never experienced that before? So is that what made it feel uncertain or made that feel, you know kind of uncomfortable?

**P1**

I think it's a real mix of what you've said. I think mainly it just being new was the most uncertain. I can deal with fast-paced stuff. I even don't mind being put into unsteady waters for a minute, but it's just like the feeling of coming to uni. It's like from then on you're never really certain about anything again. If you really think about it, it's quite a lot to manage. It's everything you were saying, it's the independence, it's the new environments. You're being treated like an adult, but you're not really. And it's just a lot of things. I moved to university. I didn't know anyone who was coming to university. Like, you're making friendships, you're having relationships. It's just like everything is so uncertain because it's just, all so new. And then everyone's at different stages in their lives and everyone's gone through different things. I feel like at home you can be in quite a bubble in secondary school because you've grown up with these people, you're used to them, you're used to seeing them every day. You have a school routine you hang out with the same people on the weekend, then you go home and you have meals done for you and whatever. Then here it's you're seeing different people every day and spending time with different people and doing new things and like I don't know. It's just a lot. Everything adds to the uncertainty of it. Like if it was just a new environment or just new people, I think it would be less uncertain. But I think it's the fact that going to university and going to medical school, it's all happening at once and that's what makes it so uncertain.

**IK**

Yeah. So lots of things happening at once, but have you had that uncertainty experience before or was that just something you've never experienced before and that's just all brand new now at medical school?

**P1**

Yeah, I faced a lot of uncertainty in life, but there's always someone or something protecting you when you're younger. When you're applying to school, my parents helped me all through it or if I was uncertain about a friendship or something happened, there would be other things in place that I was certain about. So I think it's OK. I felt uncertainty in my life. But when you're young and you're surrounded by so much certainty alongside things that are different it doesn't feel as bad. It doesn't feel as momentous. But when you're coming to uni, there's more uncertainty than there is certainty and therefore it makes more of an impact in day-to-day life.

**IK**

Yeah. And you said like this certainty. So what made it feel certain to you? So I think you said family as a key way of making things become certain.

**P1**

Just like familiarity. Routine. Those kind of things make…I mean, nothing in life is certain if you really think about it. But when you're young, you don't have time or you don't have the ability to think about things not going right because you're young and you're, you know, full of joy or whatever, and you're going on play dates and you're just having fun. Just kind of living life. Like you don't have responsibilities and therefore uncertainty is so trivial and therefore, most things just feel quite certain in your life. But as soon as you're placed in a new environment and you have all these responsibilities, things that may have felt certain before will suddenly feel uncertain because you've come to the realisation that nothing is really for certain and things can change and things can happen.

**IK**

OK. Thank you. So we've talked about uncertainty, but how would you typically approach it, because I can see that you're saying there's lots of things that can be uncertain in life? So how do you go about trying to navigate yourself in those situations? So you could talk about anything you want. But it could be about med school or clinical scenarios, anything like that.

**P1**

How do I cope with uncertainty? I just…give it my best shot. I don't know. I feel like there's no other way of, really…I mean, there are people that I speak to about it. I'll always speak to my mum about it or I speak to my sister or it's always nice to know people are going through the same things as you. So I think especially in medical school, everyone's going through the same things. Everyone's uncertain about the same things. And that mutual uncertainty can make things feel a lot better because you're all kind of going through it together. So I spoke a lot to my flatmates about it and you make friends very quickly through the uncertainty of med school, because everyone's like, “Oh, we all feel the same way”. But there's a beauty to being uncomfortable and being in a new environment because that's just the beauty of growing up and having new experiences is that you're not going to feel safe or certain all the time. So I feel like it's not a bad thing to be uncertain or have uncertainty, but it's just something that I think you have to recognise is something that you're going through and think, “Am I dealing with it in the appropriate way?” So if you're feeling uncertain or uncomfortable, you have to sit with it and you have to say, “OK, I'm feeling uncomfortable. I’m feeling uncertain. What am I going to do? Am I going to meet up with some old friends? Am I going to call my mum this evening? Am I going to go to this new society thing and try and feel certain in some friendships”. I mean, you can't just sit with it and then just hope that it passes and not do anything about it. That's my view.

**IK**

Yeah. Yeah. So you one thing is trying to be with people that have the same views as you could help you and then you kind of putting yourself out, you know, you have to do something, take the initiative. So yeah, OK.

**IK**

So just reading out to you, in a scoping review, so a review of a lot of articles together, there's been multiple studies that have shown that creativity that can enhance or can help tolerance of uncertainty, so helping you deal with it essentially. So what are your views on this? So what do you think about that?

**P1**

I think personally I would completely agree with it and I think being creative is a really, if you are a creative person and you use that as a means of expression and it's something that comes naturally to you, then it is going to help you in times of uncertainty. Whether you realise it or not. But I don't know if that's true for people who aren’t naturally creative, because if you're not naturally creative and you're trying to deal with uncertainty trying to be creative through that uncertainty might just lead to more uncertainty and more feelings of like loss and more feelings of, “Oh, I don't really know how to cope with it”. Like everyone copes with things differently. If you're a creative person and then you recognise that you’re just naturally drawn to doing something creative to help yourself relax or feel certain, then that's great and that's going to help you. But if you're not naturally drawn to it or you've tried it and it hasn't worked before, then it's not going to work for you. I think it's a very personal thing, being creative. But then also being creative means different things to different people. I don't know, going on a walk and writing a story in your head to take your mind off things could be creative to someone. Not everyone sits down and writes a song or makes a pot, I don't know.

**IK**

So why do you think that some people are naturally creative and some people are not?

**P1**

I think it's just what you've been exposed to. I think if in childhood or in youth you've used a creative means and you've enjoyed them, then as you grow up naturally it's going to come to you as you felt a sense of comfort in being creative before. So you're going to feel it again and again and that's kind of an expected. Like, that's a certainty in feeling better through a creative means. But if you haven't had that exposure or you didn't enjoy it or there are negative connotations associated to it when you've tried it before you're not going to feel the same natural want to do creative things. So it's not of a natural thing and more of a past thing. I think if you've had no creative experience, you're probably less likely as you grow older to try something new because you're going to just stick to what you're comfortable with and what you know. Whereas if you've had exposure to it when you're younger, then I guess naturally it's going to come to you.

**IK**

OK. Thank you. So do you have any examples where you've had creativity help you in dealing with uncertainty? So I know you said that some people are naturally creative, some are not. But if I'm right, you've had some creative experiences in the past. So do you have examples where creativity might have helped you in dealing with those uncertainties that you described earlier?

**P1**

Well, yeah. Well, I love art and I love studying art. I did it as when I was younger and that's something that's come naturally or I've just been exposed to it and I've enjoyed it, so a lot of the times even if it's just passively enjoying art. So I go to an art exhibition or even just discussing an art piece. Sometimes when I'm stressed, I just like to tell someone about a piece of artwork that I've particularly enjoyed or I particularly find fascinating and it just helps me calm down and it's kind of something that I didn't even realise helped me until I realised I was doing it. I think someone turned to me and they were just like, “Oh, you're really stressed”. And I was like, “I just want to tell you. I'm just going to speak for 30 minutes and then I'm just going to feel better. And I literally just told them everything there was to know about this particular movement in art. And then I just felt so much better. That's not even being creative in a sense, that's just using my creativity and my interest in creativity to help me.

**IK**

Yeah. So do you think that creativity itself allowed you to share because you're saying that you were telling somebody an art movement? Could you say more about like the sharing aspect of it, because you were sharing?

**P1**

OK. Well, yeah, I guess my creativity allows me to have the knowledge to be able to share with other people. So I don't know It depends. I think some days I'll just want to colour and I'll want to fill something in, paint a mug, something silly. Other days I'll want to share knowledge about a painting that I find fascinating and then share the creativity through that means because not everyone's interested in being creative. But if it's something that I enjoy, then I'll want to share it with other people because it's a passion of mine. So I think being inherently creative or enjoying things of a creative nature helps.

**IK**
OK. So how might creative approaches be helpful in clinical practice? So we were trying to talk about you as a person and your own creative approaches. But how do you think it might be helpful in clinical practice or at med school or in general you know for somebody?

**P1**

I think a lot of people find it difficult to acknowledge their own emotions or their own thoughts about a certain situation or placement or conversation or whatever. I think unless you're used to having those kind of conversations with yourself, it doesn't come naturally to a lot of people. And I think in personal experience, if I've dealt with something particularly difficult or I've had a conversation or something sparked something in me that has made me question how I deal with the situation or how I view a certain patient or conversation or situation or whatever, then I think being creative can be a really good way of understanding yourself as a clinician or as a healthcare professional in that kind of setting and who you are and different scenarios. You're faced with so many different people and if you don't understand them face value and then you sit down with that and you're like, “OK, why don't I understand what someone's been through? Why is this difficult for me to process?” Maybe being able to express it creatively or use a creative means to understand might help and therefore better you as a doctor because at the end of the day I think if you are creative and you can use different mediums to express yourselves and therefore I just think you can see the world more 3D. As doctors or healthcare professionals, you need to see someone as 3D. You can't just see them as the medication they're on or the disease they have. You have to see them as a whole. Like everyone looks at a piece of artwork differently, everyone's going to look at a patient differently and then you use all of your knowledge to treat the patient. So if you as an individual can look at someone through different avenues, then you're going to be able to provide the best care is my personal opinion.

**IK**

And just really curious to know what you said. One thing you said is understanding self. So it'd be great to know how the creativity made you understand yourself in your personal opinion. Also you said this idea of 3D which is interesting. So could you tell me more about the 3D of a patient or 3D of yourself? What you meant by 3D.

**P1**

OK, so understanding self. I think we have to know ourselves as human beings before we can be tasked to treating someone else and understanding someone else. There's a beauty in knowing, our own limits, our own psychology, our own thoughts and emotions and what we're comfortable with before going out and treating people because we'll have lives in our hands. I think if you don't understand yourself, the person holding this life, how are you supposed to keep this person safe? If you don't know who you are, then how are you best treating the person that is in front of you? I don't know if I'm articulating that well, but I just think that you can know everything there is to know about a disease or everything there is to know about medication. But if you can't hear or you can't listen or understand someone, it's just you're not going to be able to treat them efficiently. In terms of 3D it's this whole, holistic approach to care that sometimes someone just wants to be listened to or they're like giving you non verbal signs of something that they're not saying with their words. And it's being able to hear the patient, listen to the patient through all of the disease and medication and understand them as a human being. But at the same time that's a lot. Doctors have to deal with a lot of stuff they have to deal with a lot of people talking to them and putting their grief and putting their problems. So we need to be able to understand ourselves, so we don't get affected by what we see every day. We see a lot of horrible things on the wards and we hear a lot of people say difficult things to us. So we need to be able to go home at the end of the day and sit with that information and say, “OK, this is affecting me in this way. This is how I'm going to process it”. And if you don't do that from an early age through medical school, then you're not going to be able to do it as a doctor and then things are going to affect you and then affect your practice.

**IK**

Yeah. OK. Thank you. And now moving on to the challenges. So we've talked about how creativity can help you. You said it helps you understand yourself, makes you listen and then recognise things early on and pick them up instead of letting them linger. But the other side is how could creative approaches actually be unhelpful in a way? Or could they actually be challenging?

**P1**

I think the main challenge of it just comes from sitting with yourself and being uncomfortable because if you're already uncertain about a situation, there's an art to making yourself feel more uncomfortable, to become comfortable. I think that's the challenge in being creative. A lot of emotions that we have, we don't recognise as having those emotions because they're uncomfortable or they're uncertain and that we just want them to pass. No one wants to sit in a feeling of grief or a feeling of sadness or anxiety for a prolonged amount of time. So to be able to be fully creative and fully reap the benefits of being creative is to sit with uncomfortable emotions. From personal experience, when the art is difficult or being creative is difficult, you feel better after you complete it. It's just a transparent method, but you have to have the ability to ‘A’ accept your feeling it as a motion and then ‘B’ sit with it long enough to create it into something. So let's say you're feeling sad about something, me being able to draw it out onto a piece of paper and transfer some of that sadness onto the piece of paper helps. But for me to do that I have to really sit with, “What's making me feel sad? Why am I feeling sad?”, which could make me feel more sad or more upset. But then once it's on that paper, there's a certain level of detachment from it that makes me feel better at the end of the day. But that's not the same for everyone. Some people will just start feeling better through creativity or it won't help at all. From a personal experience, sometimes you have to get more uncomfortable to really make the art express what you want it to express or mean something, whether it's to yourself or to show anybody else.

**IK**

Yeah. Thank you. And then moving on to the last one is just a summary. So is there anything you'd like to share at all regarding the role of creativity in tolerating uncertainty, in clinical practise. I think you've already said a lot, but is there anything else you'd like to add or are there any questions you would like to have been asked?

**P1**

Not really, I think I said a lot of things. I think if creativity works for you, then it should be something that you should explore. I think everyone should be open to being creative and at least try it once or twice before they completely give up on it. But then I also understand that not everyone expresses themselves in the same way. So what works for me in dealing with uncertainty might not work for the next person, but I find particular comfort in knowing that I have creativity in times of uncertainty.

**IK**

Yeah. OK. Thank you. So just to summarise what we've talked about you started off talking about how you think creativity is the idea of expressing one's thoughts and sometimes it can come from moments. And then we talked about flourishing and the idea of how it helped you with stress and allowed you to look at things in different ways, different lenses. Then we were looking at how there's a lot of uncertainty everywhere at med school and it's quite tough to pinpoint where it is. But at a personal level, creativity has helped you through and not everyone gets creativity naturally, as you said, and it might be hard for others to use that as a way. But for yourself, it's helped you and it can be challenging sometimes to use that as a way through uncertainty in different scenarios with patients or how you want to become as a clinician or how you are now. So I hope that's a good representation of what you said.

**P1**

Yeah.

**IK**

But if there's nothing else you'd like to say, thank you very much and I’d like to end the interview now.
