# Supplementary material for: How does creative enquiry enable us to deal with uncertainty?
Source: BMC Med Educ. 2026 Aug 8;26:1785. doi: 10.1186/s12909-026-10060-w (PMC13732821; doi:10.1186/s12909-026-10060-w)
Supplement: Supplementary file 11 [file 12909_2026_10060_MOESM8_ESM.docx]

**IK**
What does creativity mean to you as a medical student?

**P2**
Hmm…well…as a medical student…I think…creativity might have just meant things outside of medical school, but engaging in creative inquiry, I feel like I've been able to…kind of amalgamate creativity in medicine. And yeah, kind of finding…pockets of…creative inspiration in medicine, definitely for me.

**IK**
OK and I think there's two things you said. One was, you saw creativity outside of medical school and from listening to you from last week you've already studied different degrees: biomedical science and the medical humanities. So I might open up the question more if that was a bit closed just to ask about as a medical student, because you've been in different roles. So it might be better to ask what does creativity mean to you just generally in all of the things you've studied but also if you could explain what you meant by saying outside of medical school? Why is the creativity outside of medical school?

**P2**
Well, I guess before I ever did like the creative enquiry SSC with Louise. I…wouldn’t have seen myself as being like a creative in medical school, like a creative person. I was interested in arts and humanities and medical humanities, but I hadn't considered myself being like the active participant in the creativity before I had come to medical school. So yeah, that's something that really has just developed since being at medical school. And then what does creativity mean to me? Well…I guess for me it means like making connections and getting into kind of like a flow state. That's what it's like for me when I'm being creative.

**IK**
Yeah, and what made medical school not creative, or why do you see that the creativity has to be outside?

**P2**
I guess…it's the emphasis on the heavy kind of…well, yeah, the emphasis and the heavy focusing on the biomedical sciences. I mean, even with all the time that we do dedicate to that stuff, it still feels like, I don't know, there's never enough time to cover everything.

**IK**
Yeah, so you've got too much of the theory and clinical knowledge that you don't have that time to be creative.

**P2**
Yeah, like sometimes it feels as if, like being creative has to be relegated to second up, you know.

**IK**
Yeah, true. Thank you. And then the next part. You've already had loads of experiences, but could you talk about an example or you could talk about more than one where you've used creativity in the past to help you reflect on clinical practice or anything you've done?

**P2**
Yeah, for sure. Well, throughout the years and especially since doing the SSC, I've used poetry writing to reflect on clinical practice. For example, when I was on my GP placement in third year as a reflective piece, I wrote a poem on tackling obesity in primary care. But it was kind of like more of a first person perspective. And then my fourth year dissertation was on poetry and grief, I guess my own personal experience, but I hope that through exploring it in a creative way, I can kind of put that into practice, the things that I've learned. And what else? Oh I wrote a poem not so long ago. This is in November of last year, reflecting on anaesthetics. And I guess again like my kind of personal and professional selves, in relation to patients and things like that.

**IK**
Yeah. And then you said something about it allowing you to put what you said into practise what you were seeing or what you were doing. So why use creativity to express those things? Surely you could have done a portfolio or could have just written something? So what was unique about the poem?

**P2**
Well, I think it …it kind of like…Well, at least for me it allows for more authentic reflection as opposed to having to reflect on principles of good medical practise, which feels a bit stilted and it feels less human as well. I don't know yet. It kind of reconnects me to the human side of medicine I feel.

**IK**
Yeah. And that's what you mean by the authentic. And then could you describe an instance where you've had to face considerable uncertainty? So it could be in life. It could be during medical school. It could be before. Is there something that as I tell you the question, probably there might be something that stands out to you? And if so if that's OK, could you talk me through how that felt and what made that uncertain to other events in your life?

**P2**
My major uncertainty that I've experienced has probably been through my mum's illness. Because she had MS and I guess it was kind of like the ambiguity and the uncertainty of her being physically present but psychologically absent. The uncertainty of her, ”It's her there,” but she's so different and not knowing exactly the trajectory of the illness and things like that. That's probably like the major uncertainty and kind of being like, “I don't know if I'm coming or going like what the hell”.

**IK**
Yeah. Yeah. Thank you for sharing because I’ve not being in a situation like that, but I kind of can see what you're saying about being physically present, but psychologically absent. So how did you try to deal with that? Because I'm sure that must be something.

**P2**
Well…I guess I tried to make sense of it through writing poems. That definitely helped. I guess I would say this is kind of like the conclusion that I came to with my dissertation. Some things just will never make sense but that's something that you can accept. Through writing poems I was able to like explore that.

**IK**
Yeah. And you were saying sense-making. So I think even for me I'm trying to understand myself when I try to explain to somebody that creativity can essentially make you make sense of situations and a lot of people are sceptical about that. So it's really interesting you talked about sense-making idea. So how did the poetry help you make sense of the situation?

**P2**
I'm sure there are lots of different practises that people could explore, but for me like just spending time with the pen and paper like without other distractions and just seeing what comes out. And then going back to the flow state amending the words on the page and then you kind of just get stuck into it and then you come away and you've got a sense of like, “Oh”. You see things in a different light or maybe you have a different perspective on something or even if you don't you spent some time thinking about it and you have something that you're proud of at least.

**IK**
So it gives you that space to let you remove those distractions and just focus on yourself in a way. I hope I'm summarising it right. Just so that I can understand.

**P2**
Yeah, yeah, that makes sense.

**IK**
OK. Thank you. And I'm just reading out something now to you. So in a scoping review, there have been multiple studies that have shown that creativity can enhance the tolerance of uncertainty. So what are your views on this and why?

**P2**
To engage in the creative process, you have to get comfortable with being uncertain because you don't know what's going to come out. So I guess if you're creating something which is personal, there's a sense of vulnerability. I don't know. I'm not sure how to describe it. Please can you repeat the quote?

**IK**
Yeah, so in a scoping review there have been loads of studies and they've said that by using creativity, so they've done it on music, dance, arts-based museum work, essentially they can all enhance you tolerating uncertainty. And my question was what do you think about that? Do you agree with that? Do you not and kind of why? Why do you think if so?

**P2**
I do agree with it, but I'm not sure how to put into words like why…Like…I think...maybe through like creative practices or exploring or experiencing creative things...maybe you just open yourself up to be more receptive to things being uncertain. And then you can better tolerate uncertainty...Yeah, I'm not really sure.

**IK**
It might be better if I frame it again to your own experiences and going back to the how did the creative enquiry help. How did that help you? Or did you see a change in yourself from doing the creative enquiry.

**P2**
Well…I think…Like…through creative enquiry it gives you a safe space to be in uncertainty. And then when you spend some time in it, then it feels more tolerable moving forwards. So being with it and not shying away from it.

**IK**
Thank you, because you can already tell that it is a difficult topic. A lot of people think about ‘how’ because everyone I'm interviewing have experienced creative enquiry but it's sometimes hard to define how does it help with the uncertainty so thank you. But if that’s OK I can move on to the next question.

**P2**
Yeah, sure.

**IK**
So do you have any examples of creative approaches where you've had to engage with tolerating uncertainty? You were already talking about your mother, but it might be a different example, just to hear a different perspective or if you've done something different to poetry.

**P2**

Oh well, I suppose when I took time out, when I interrupted my studies, then I was doing a lot of different creative things that weren't poetry but were still creative. So when I was working for my friend doing creative workshops, we were helping people with pottery and making terrariums with plants in.

**P2**
And I think that was really valuable to me because it was being creative, but in a more tactile way as opposed to a cognitive way with writing poems. It was more about using my hands and I found that quite comforting going back to doing those kinds of things because I hadn’t done that in a long time. There was a child-like enjoyment and exploration of things.

**IK**
Yeah. And as you say it’s hard to express in words. If I’m right that’s why you’re using a creative way like the pottery or the poetry to express yourself in a way, without words. Well, you are using words but in a different way.

**P2**
Yeah, exactly.

**IK**
But there's also one thing I just remembered. I think earlier you were talking about in third year you wrote a poem about obesity but you chose to do that in first person.

**P2**
Some of it was in first person, yeah.

**IK**
So if that's OK to ask, why first person or why did the poetry allow you to speak in that kind of voice? Or did it give you a voice?

**P2**
Well, to be honest, before coming to medical school, I had a judgmental opinion on people who were obese but then after coming to medical school and learning about health inequalities and obesogenic environments that we live in, my views kind of changed and then I was able to put that into the poem. Just thinking more about someone managing different comorbid illnesses alongside being obese. So it's kind of like a bit about diabetes or someone being diagnosed with diabetes and their anxiety with that.

**IK**
Yeah. And there was another one where you were saying in fourth year you did a poem about grief. I'm sure that's a big emotion, but how did the poetry help you express it?

**P2**
Well…I guess… with grief, for me at least anyway, sometimes it feels kind of impossible to put into words. I guess through poems, especially being a grieving medical student, I can give not necessarily a unique perspective, but even just to share my experience or how I feel, it can be a window into what it's like to be grieving. Even expressing it helps because it makes, for me at least, less isolated.

**IK**
So have you tried to share your poem with other people or have people read it and then you talk to them about it and how was that for you?

**P2**
Yeah. Definitely I've had that experience. Most of what I write I share on my Instagram page and then mostly friends or sometimes people I don't know will like say that it resonated, and for me that's so meaningful because they aren't necessarily having an identical experience, but they can still find something valuable from the poem. I guess that's also what I like about writing poems. They're up for interpretation.

**IK**
Yeah. Yeah, they’re up for interpretation. So you can take different views on the same thing.

**P2**
Yeah, exactly. And you don't even necessarily have to be explicit about what you're talking about or what you're trying to get across. I might personally know exactly what I'm trying to say or I know what experience I'm reflecting on but other people might not necessarily get that exact thing. There's still value in it for them or it resonates in a different way.

**IK**
Yeah. But then does it allow you to fully put your emotion of that experience in a way on the paper? You were saying that you're thinking of a specific experience or a specific thing, but probably only you know that So when you're using poetry, do you think you can fully put that idea onto the page or in a way that you can remember?

**P2**
Yeah, I think so. It’s more about the emotion that's conveyed or the feeling that's conveyed and even for people that haven't had the same experience, they might still have the same kind of feeling, because the feelings are universal.

**IK**
Yeah, thank you. And I'll move you on to the next question because of time. I think you're saying really interesting things, so that's why I wanted to hear more. The next one was how might creative approaches be helpful in in clinical practice? You could answer with whatever you want, but I'm just saying that there was something you said earlier, just to remind yourself. I think you were talking about, you talked about the difference as a medical student and then clinical practice and that was your most recent poem. And you were already kind of talking to me and other people that on last week about how you're going into this new chapter of your life and you're excited, but you're also thinking about what's going on. So just to hear your perspective on that. And more about that poem because I missed half of it as well.

**P2**
That poem that I read it was one that I wrote pretty much this time last year when I was on obs & gynae in Harlow. A lot of what I write is related to grief, to be honest, but it was about grieving and being on obs & gynae and Mother's Day was approaching. Obs & gynae, for me at least, the new beginnings and certain losses felt so palpable. It was everywhere. That's why I was thinking about in that one. But then more recently, I've been reflecting on how I balance my personal feelings with the suffering that we'll see in clinical practice and already see as medical students. But it maybe it will feel more different when you're like actively involved in someone's care. How can I stay compassionate without being weighed down by another person's story or suffering? How can I kind of relate to what they're going through?

**IK**
Yeah. I was just thinking through the ideas that you were saying so, thank you. Looking at how it could be unhelpful because you talked about grief and It is a big emotion and I'm guessing from what you're saying that it's a way to process it. Also you were talking about other things as well, but have you ever found it unhelpful? Whilst doing creative stuff, do you think it hindered you?

**P2**
Not exactly. I mean, to be honest it makes me feel more inspired, but then at the same time I guess everything is kind of like painted with a brush of grief. I do feel like at the moment where I'm preparing for finals, I feel like I need to stay surface level with how much I go into my grief feelings or whatever because it's like I've got more important things to do, which feels like I don't want to have to deny my feelings, but you know, exams are exams.

**IK**
So you're saying that sometimes the creativity can make you open up too much that you need to focus on tasks and things you have to do now.

**P2**
Yeah, that's my experience.

**IK**
Yeah. OK, thank you. And then just the last thing that if there's anything else you'd like to add that I probably didn't ask you or you wish you wanted to say? It can be on anything on the role of creativity or uncertainty. Anything you want to say.

**P2**
I'm not sure. I guess maybe reflecting on the event last week and like today, I see how like engaging creative enquiry has really like transformed my experience as a medical student and well, not just as a medical student, as a person like as a whole. Yeah, because before I tried creative enquiry myself, I wouldn't have worked through things or feelings or experiences with a creative practice, but now I do and that's become a regular thing for me because it's been a few years. It's really been transformative for me. I was interested in like, medical humanities, but I didn't see myself as someone who create things in that field. I was more just studying it and now I really can't imagine myself not writing and I want it to be a part of my career in the future as well.

**IK**
And you're saying that you do it regularly, so why do it regularly?

**P2**
It feels good. It's cathartic. I like the feeling of connection when people see my work and I get their feedback. I like it.

**IK**

Yeah. Thank you for your time.
