# Supplementary material for: How does creative enquiry enable us to deal with uncertainty?
Source: BMC Med Educ. 2026 Aug 8;26:1785. doi: 10.1186/s12909-026-10060-w (PMC13732821; doi:10.1186/s12909-026-10060-w)
Supplement: Supplementary file 12 [file 12909_2026_10060_MOESM9_ESM.docx]

**IK**
What does creativity mean to you as a student?

**P3**
Hmm, that's a good question, I think it's just a form of expression. A way to express your thoughts, your feelings, the ability to be able to create in a way that is free and without limitations and I feel that's the main thing. Especially as a medical student, it's a different way of thinking. I think we are encouraged to be quite logical and have that kind of approach in our studies. But being creative for me is kind of going outside those bounds and being able to explore without the key kind of constrictions of logic or science.

**IK**
Yeah. And what do you mean by outside of that logical sense?

**P3**
I think in the creative space, everything is sort of valid. There is no right answer. There's no right or wrong way to do things. It's about individual expression. It's about allowing yourself to explore without any kind of expectation or answer which I think is something that is important to not only your well-being but your critical thinking. I think it's really vital to keep that alive.

**IK**
And is that what makes you enjoy creativity or?

**P3**
I think creativity for me is more than anything more of a cathartic process. It's more about processing thoughts, feelings and often I find before starting a creative project I don't even know what I'm going to do. Say writing a song, I just have a small idea and then as it goes on, I've found it's actually a way that we process the things that happen in our lives. I think that's something that is intrinsic in us and human beings. We were doing it since the beginning of time. We want to take what we see and what we experience and kind of put it in an art form. And it's a way of almost connecting with others as well.

There's so many facets to what makes creativity important.

**IK**
Yeah. Thank you. And now thinking about a specific example of creativity that you've used in the past to reflect?

**P3**
With the song that I did, it's something that I sort of experienced a while ago. I feel like I gained so many valuable lessons from those experiences and you see it from a new perspective. I think creativity is so vital to empathy as well and understanding other people's stories because we can sort of understand them on a surface level. But by using creative mediums, we understand the emotions behind it.

Understanding other people's perspectives are really important and I think that's what I gained by doing this song because it was less of about me. It was about a patient and it allowed me to step out of my own perspective and go into hers and see what she was feeling and really empathise. But I think also it works two ways, because you then evaluate your own role and in a certain situation. So say if you've gone through something difficult, it's often that we are hard on ourselves. I think by going through the creative process, we learn to be kind of to ourselves, to have empathy and kindness for ourselves and others, which I think is important.

**IK**
How is it that that song made you kind of empathise or make you think emotionally?

**P3**
The experience of seeing a patient that I grew close to deteriorate during my time at the hospice was tough. Ultimately we have to recognise that the struggles that they're going through are so individual, and they are so unique. We can't forget their internal struggles and what they're going through. So that was where I was kind of seeing it from, from that lens.

**IK**

Could you talk about a time that was uncertain?

**P3**
Honestly, there have been so many times. If I look back on it, I think I often find it's quite ironic when I'm almost the most stressed and there is the most uncertainty, I use a creative medium as a coping mechanism. So even during exams and it's quite stressful. I've got a lot but that's actually when the creative ideas start coming. It's almost like my brain's way of saying, “Let's take a step back and mentally process these feelings of uncertainty”.

**P3**
And if you just sit down for a second and step out of your head for a second. You don't just go in the same sort of thought cycles. You have to be reflective. You have to rely on the creative process. And I think that's why the freeing feeling, I think when we're dealing with uncertainty and maybe anxiety, we're always having to think, “Oh God, maybe I could do this or that”. But the creative process is almost an ‘out of your hands’ in a way, and that I find to be quite freeing.

**IK**
Why use creativity? Because.

**P3**
We can't sort of neglect that part of ourselves. I think often when we're most stressed about things or we hyper fixate on certain things, it's because we have almost forgotten to create. I feel like each person has a plant inside. You have to kind of water that, create and nurture that creative spirit and that side of yourself. Otherwise I think there is an element of when we think that we have complete control over things, that's when we end up in stressful situations. I feel like with other coping mechanisms for me personally, there's almost a pressure associated with it. For example, meditation has never really resonated with me because I have to be doing it properly or I have to be going up to the standard. But with creativity there isn't it right or wrong. I just have the ability to be free with my creative expression.

And I know no one ever has to see the songs. It's not like you do a piece of work for uni where there’s a grade to it. There is a standard that you have to adhere to, but with creativity, you have the ability to say who you want to share it with and what it looks like. And I think that's incredibly powerful. And it can empower you to take that initiative in your life, I guess.

0:10:59.480 --> 0:11:4.800
**IK**
Yeah. Thank you. So I didn't think of it kind of making it as you're saying.

0:11:5.920 --> 0:11:9.760
**IK**
It allows you to be free because there's no expectation. If I'm right.

0:11:15.900 --> 0:11:16.20
**P3**
Hmm.

0:11:11.240 --> 0:11:16.200
**IK**
But you also said earlier this idea of helping you step outside so.

0:11:18.650 --> 0:11:35.850
**IK**
And at the same time it is part of you because you're saying it's like this kind of creative spirit in everyone where everyone has this part that needs to be nurtured. So how does this thing that's part of you if I'm right?

0:11:37.410 --> 0:11:38.330
**IK**
Or P everyone.

0:11:40.150 --> 0:11:46.150
**IK**
Also make you step outside of yourself and what you really meant by that. I hope I'm clear, but.

0:11:47.180 --> 0:11:47.980
**P3**
Yeah, yeah.

0:11:49.380 --> 0:12:14.100
**P3**
I guess it's in a way like for me when I feel like I have creative ideas, I don't feel like it's a very conscious process. It's almost unconscious. You can't control it in a way. If the idea comes, you just kind of have to work with it. You have to do what you have to do. Say it. Like for me, I have to, like, write down the song. I have to get it down. And I think that feeling I feel like just can't be replicated with anything else. But it's almost like.

0:12:15.460 --> 0:12:20.900
**P3**
A yeah, it's like this, almost meditative process, especially if I've done it so many times, sort of throughout my life, it feels.

0:12:22.420 --> 0:12:27.20
**P3**
Yeah, it's quite cathartic. Very, like meditative it feels.

0:12:28.700 --> 0:12:42.780
**P3**
Really comforting, I think. And I think every each person has that different thing for them that gives them comfort, makes them feel like they're able to. So we talked about flourishing a lot in the SSC, like feel like they're flourishing, feeling like they're able to.

0:12:45.110 --> 0:13:16.350
**P3**
I don't know. It's hard. It's hard to articulate. But yeah, I think it's it's a huge feeling that I I don't think can be replicated with other forms of work. There's something about the creative process and I think also being humble in the creative process because with other work that you do, there is a sense of you have to play, put the hours in, you have to. But with creativity, of course you can homeless skills, but there is an element of it which you almost feel like it's it's like it's like a gift to you that you have to.

0:13:17.110 --> 0:13:28.950
**P3**
When you get that moment of creativity, it's almost like your responsibility. You have no control over it, which I quite like and makes it. Yeah, like I mentioned earlier, you don't have that stress of that expectation.

0:13:29.820 --> 0:13:34.660
**IK**
Yeah. Thank you. That I'd, I've never had the idea of, like cumbling because.

0:13:35.940 --> 0:13:38.100
**IK**
Normally people think of it.

0:13:38.380 --> 0:13:43.740
**IK**
In different ways, but humbling is another way of looking at it, so thank you.

0:13:46.0 --> 0:13:50.0
**IK**
And do you think there's anything else because you've said you?

0:13:50.40 --> 0:14:5.920
**IK**
You've used creativity loads of times, so apart from the song, has there been another way? It might be not music, it might be a different type of art form or another song that you've used in the past.

0:14:9.250 --> 0:14:26.770
**IK**
It would be great to hear from from you on that on that kind of thing. And if if you've ever used it in a kind of uncertain situation. So I think the one with the Hospice that you were saying last time is an uncertain situation. But I mean something else as well.

0:14:28.170 --> 0:14:29.370
**IK**
What have you think?

0:14:30.290 --> 0:14:53.570
**P3**
Yeah, I mean, there have been so many sort of areas throughout my life. I think that personal sort of family sort of instances or even things with friends or I think if you go through anything that is rough or emotional and that's when you feel it's a way of getting off your chest. Some people do journaling, obviously, like talking to people helps, but I find.

0:14:55.90 --> 0:15:1.90
**P3**
My way of affecting on things and processing them is very much through creative media. I also really like to paint as well.

0:15:1.480 --> 0:15:7.360
**P3**
Find 3 nights. I like the that sort of element of expression.

0:15:8.960 --> 0:15:33.400
**P3**
As another way to do creativity. Because I think with songwriting, sometimes I feel it can be a little bit limited and also I'm almost too much like practise of it too much where I know kind of the steps and if I want to get outside of that I might say do painting or even writing. I find like really sort of even like things like short stories and whatnot. I think it's dealing with uncertainty we.

0:15:33.880 --> 0:16:3.520
**P3**
The only thing you really can do because in certain situations some adult what? How you approach it. There's always going to be that that uncertainty. So by using creative media, you process more of the feelings of it and you come to accept that some things are uncertain because the creative process itself is uncertain. So I think it that's why it works so well dealing with those kind of kind of situations, I suppose. So even in my own life when.

0:16:4.320 --> 0:16:8.640
**P3**
I know like when my grandparents that help is kind of getting worse. That was one case where.

0:16:9.630 --> 0:16:26.950
**P3**
I I I felt myself sort of going back and and feeling the needs to sort of process my my emotions in that way, which was important. But no, I think it'll always be a part of how I approach life and reflect on experiences that I've had.

0:16:26.990 --> 0:16:40.830
**P3**
And I think I'm I'm I think I was meant to say something. I forgot what it was. Yeah. I want to say, like, when I'm being creative, I think it's more that I just feel like I am the vessel vessel for the creative idea. Like it's it's not really about me. It's about.

0:16:41.360 --> 0:17:13.440
**P3**
So I have this creative thing that needs to come out and then it's off. I've actually made it, I think. Oh, actually this is actually about my life. This is a reflection of me and I'm able to actually better understand a situation than I did when it was before. So obviously I'm writing about this hypothetical person, and there have been so many countless times where I look back and think, oh, OK, this is my way of mentally processing it right. It makes sense. It's almost like the repeat. Like, I don't go into it trying to reflect or process things, but then it's just almost like a nice side effect. And then I also have this thing that I've produced that.

0:17:14.80 --> 0:17:17.440
**P3**
I can then share with other people and then if they've felt in a similar way.

0:17:17.960 --> 0:17:20.360
**P3**
And it might resonate with them, which is just the best feeling.

0:17:21.80 --> 0:17:30.680
**IK**
Yeah. And then there's a point, she said very quickly at the end. So just ask about, I just kind of repeating it and then ask you if you want to say more.

0:17:33.320 --> 0:17:35.160
**IK**
I think the first thing you were saying is.

0:17:37.300 --> 0:17:40.300
**IK**
Do you do different forms of art so?

0:17:43.420 --> 0:17:52.300
**IK**
If I'm right, the music might be something that you know a lot about so that you said it kind of limits you so you do something else where.

0:17:53.940 --> 0:17:58.940
**IK**
Not where you might not necessarily have honed your skills as much on it so.

0:17:58.870 --> 0:17:58.990
**P3**
And.

0:18:4.290 --> 0:18:4.370
**P3**
Oh.

0:18:8.510 --> 0:18:8.630
**P3**
But.

0:18:1.510 --> 0:18:9.110
**IK**
Painting or short stories. So just I I could ask that question one at a time. So that was one. And then I'll ask the rest.

0:18:10.630 --> 0:18:26.710
**IK**
Later, if that's OK, but so do you think that you are more creative or you feel kind of easier to kind of release or express yourself when you use an art form that you're not necessarily as?

0:18:28.170 --> 0:18:29.210
**IK**
As experienced in.

0:18:32.760 --> 0:19:1.800
**P3**
I think it does. It definitely depends on the context. I think there are certain instances where I felt that like I've tried to write a song, say about something and it just really doesn't feel right because it feels like I'm just doing it for the sake of it. Or I'm thinking more about the outcome and all what will other people think? I think, especially because even my family, they're so great and supportive. But you know, they can be like, oh, I see working on the song. Like, can we hear it? Can we, you know, like they have that kind of they really want to get involved, which is amazing.

0:19:2.190 --> 0:19:33.430
**P3**
And it's been great, but with the kind of episode, like, say, the short series, I just don't share that with anyone and it's just just purely for me. So it gives me that I, I know personally they're really bad like I'm not going to do it at all, but it's almost accepting that it makes it a fun way to again just kind of step out of myself. But I know, especially if I haven't written songs in a while, I still find that is such a valuable experience as well. So I think having different medium, I think it makes me sure you never get tired of one thing.

0:19:33.640 --> 0:19:34.920
**P3**
Or feel that.

0:19:36.680 --> 0:20:6.360
**P3**
It kind of keeps that, like I said, the creative spirit alive, always delving it like I'm always trying out a new like, I don't know whether it's, I don't know, like sewing or knitting or something like that. Like, it's always. I just like to experiment with different mediums because I think that's that's important to and almost having that variety improves. The other more. Once I attempt to work with. So I feel by experimenting it makes me feel I can make braver choices and say self writing or painting or that kind of thing.

0:20:7.450 --> 0:20:13.970
**IK**
Yeah. And then the other thing he was talking about, I think was that you're almost a vessel, so.

0:20:17.360 --> 0:20:19.880
**IK**
You are open to what comes.

0:20:20.460 --> 0:20:20.660
**P3**
Mm hmm.

0:20:21.80 --> 0:20:25.880
**IK**
And then most most of the time you find out that you actually reflecting on yourself.

0:20:28.40 --> 0:20:35.960
**IK**
And you said that your that you enjoy sharing with others, so talking about those two things a little bit more would be.

0:20:37.900 --> 0:20:39.820
**IK**
Interesting to hear from you so.

0:20:40.370 --> 0:20:40.490
**P3**
Hmm.

0:20:42.250 --> 0:21:12.290
**P3**
Yeah, I suppose I don't know if other people can relate, but I feel most of my ideas kind of come when I'm not expecting it or just suddenly something pops into my head, I think, oh gosh, I should probably sort of hold on to this because if I don't, then it might just leave me and float away. It's almost like it comes in the air and I have to grab onto it. But yeah, it just it makes you. I think there's a lack of ego in when you're doing creative stuff because it's it's less about you. It's more about.

0:21:12.650 --> 0:21:25.970
**P3**
How can I do justice to this song? How can what am I actually trying to say? That's what I'm always asking myself throughout any creative process. What am I trying to say? Whether it's a painting, whether it's a song, what am I trying to communicate? And I feel like by digging into that.

0:21:27.530 --> 0:21:41.450
**P3**
You just you have to let go of any frequency notions about yourself or what you should be doing. What other people expect of you, which I find to be quite, quite freeing and quite a powerful experience I feel like.

0:21:42.110 --> 0:21:55.710
**P3**
So yeah, I just feel I'm kind of the means to the end. And then if when I feel satisfied is when I feel that what I've created, I've done justice to this thing that was meant to be created and I was just the way for it to come about. If you go what I mean.

0:21:55.950 --> 0:22:1.230
**IK**
Yeah, yeah, I can say, yeah. And then the idea of sharing.

0:22:2.670 --> 0:22:3.230
**IK**
You you said that.

0:22:4.790 --> 0:22:15.870
**IK**
Did you enjoy sharing with people? How? How do you find it? When of course you say you share something, some you don't. But out of the ones you share, so the the music.

0:22:18.260 --> 0:22:23.700
**IK**
How do you feel when you do that for yourself and for others, really.

0:22:25.450 --> 0:22:57.810
**P3**
Yeah, it's a good feeling. I feel like I'm quite generally, I don't do very like many performances or anything like that. It's mainly just like very close friends or family. I tend to share with and yes, really nice feeling there sort of responses and the things that make makes them think of because sometimes it's not even vaguely related to my experience or it just can be something that made them think of someone or something which is really nice that they feel they feel that way. And I think it's a way that we connect as human beings. We connect through art, whether it's music.

0:22:58.210 --> 0:23:8.810
**P3**
Play these things kind of reflects on what we go through and by watching it, it makes us listening to it.

0:23:27.240 --> 0:23:27.400
**IK**
Yeah.

0:23:8.850 --> 0:23:32.130
**P3**
It makes us feel understood, I feel, and I think that is so essential that we feel connected to others, that art is such an amazing, amazing way to do that. But yeah, I still think something. I'm trying out a little bit more. I'm definitely someone I like to just kind of keep things very close to me. I'm always very nervous of what will people think as I'm kind of getting older, trying to work on that aspect of myself.

0:23:32.600 --> 0:23:37.640
**P3**
I think sharing at this I can only bring about sort of good things and.

0:23:39.120 --> 0:23:41.240
**P3**
Hopefully make a positive difference to others.

0:23:41.950 --> 0:23:43.470
**IK**
Yeah. Thank you.

0:23:45.320 --> 0:23:47.440
**IK**
And I'm moving on to the next part really of.

0:23:49.350 --> 0:23:59.270
**IK**
I think we've already kind of talked about uncertainty and you said you know about your your grandparents and how you had to deal with their house.

0:24:0.690 --> 0:24:3.170
**IK**
And different parts of your life so.

0:24:5.490 --> 0:24:16.250
**IK**
How do you typically approach uncertainty? Has it always been creativity or have you used other ways you don't have to, but have you? Is that? Is this your typical approach?

0:24:17.570 --> 0:24:19.650
**IK**
When kind of dealing with those situations.

0:24:22.280 --> 0:24:23.640
**P3**
Yeah, I mean.

0:24:25.120 --> 0:24:33.200
**P3**
I feel with uncertainty I tend to also rely on people close to me, so Brian's family, I think that that's so important to have.

0:24:34.800 --> 0:24:52.0
**P3**
Sort of network that is able to support you through dealing during the ICT, because I think often times we feel like we have to constantly be in control of our lives or in control of outcomes. But there are certain things that are out of our control and I think talking to other people can help us remind mind yourself that.

0:24:53.870 --> 0:25:20.590
**P3**
But yeah, I feel more and more that I encounter uncertainty. You become, you start to feel more comfortable with it. I was also. I worked for a while about six months or so on like a stroke board. I think at the beginning it was quite sort of overwhelming for me. I had like 12 and 1/2 hour shifts. It was a lot. I just, I obviously like some patients would often like sort of die as well. It was very high in stress environment, a lot of uncertainty involved.

0:25:21.990 --> 0:25:27.350
**P3**
And I found that more than ever, you kind of rely on the people around you and on these creative means as well.

0:25:28.30 --> 0:25:47.390
**P3**
And to give you a sense of a sense of perspective, really, I think we sometimes focus on the minute, minute sort of details or problems in our life. But I think being sort with to commit creative means of speaking to others over time. So you step back and you see the bigger picture and you understand that.

0:25:48.670 --> 0:25:57.870
**P3**
Uncertainty is a part of life. You realise that some of the more like problems seem less significant. I think in the wider scheme of things.

0:25:58.880 --> 0:26:7.320
**P3**
And then you slowly start to to learn to accept that which I think is important. But yeah, it's always it's sometimes different I also find.

0:26:8.760 --> 0:26:29.960
**P3**
Even I enjoy going out sort of nature a lot, that sort of thing and feeling connected to the world around you. I think when you get a lost in your own sort of day-to-day, that's when and you uncertainty becomes more of a problem. But if you take a moment step back, then that's when you're able to handle it a little bit better. I don't know if that really serves.

0:26:30.900 --> 0:26:33.340
**IK**
Yeah. And and.

0:26:34.420 --> 0:26:37.20
**IK**
I think you said also creative means really.

0:26:39.590 --> 0:26:39.790
**P3**
Round.

0:26:38.880 --> 0:26:44.880
**IK**
And how would you? How did that help you with your 12 and how 12 and 1/2 hour shifts because I'm guessing?

0:26:46.200 --> 0:26:49.520
**IK**
You can't do the creativity during the shift so.

0:26:51.400 --> 0:26:53.240
**IK**
How? How?

0:26:53.400 --> 0:26:54.320
**IK**
With the creativity help.

0:26:56.820 --> 0:26:56.940
**P3**
And.

0:26:55.920 --> 0:27:0.840
**IK**
Would you do it before or after? How did it help you think? Reflect on it? I don't.

0:27:2.370 --> 0:27:12.10
**P3**
Gosh, I mean, I would, I don't know. Obviously you've been doing. So I I worked as a receptionist, so I would be sort of thinking about things or even my lunch break sort of I think.

0:27:13.730 --> 0:27:16.170
**P3**
Back and I think creatively it can be a reminder.

0:27:19.970 --> 0:27:20.130
**P3**
Of.

0:27:20.330 --> 0:27:29.770
**P3**
Often because I quite I like to write from the perspective of other people who are, and I think if you're also going through a difficult time, that can be quite nice kind of step out of what you're experiencing.

0:27:31.450 --> 0:27:35.210
**P3**
But yeah, I think especially how had a really long day, I'm feeling really tired.

0:27:36.570 --> 0:27:40.290
**P3**
Being creative, it's almost like a pick me up because.

0:27:40.330 --> 0:28:2.10
**P3**
It takes you out of almost something that might feel monotonous or just have as a reminder that you are able to produce something of value. You are able to like. Give something to society. I think oftentimes we set really high goals for ourselves in life and we think, oh, I need to achieve this or I need to do that. But by being creative.

0:28:2.450 --> 0:28:6.10
**P3**
Reminds us of that. Even a small thing to say. I write a song in like 20 minutes.

0:28:7.410 --> 0:28:23.210
**P3**
I have created something that has value and even I hate to someone else, there's just it's so limitless. The power that art and being creative can have, which is it's nice reminder, especially in stressful or sort of overwhelming times.

0:28:30.60 --> 0:28:30.220
**P3**
Yeah.

0:28:24.560 --> 0:28:32.280
**IK**
Yeah, thank you. And now I'm just reading up to you a fact, a statement really so.

0:28:33.680 --> 0:28:35.800
**IK**
In a scoping review.

0:28:35.900 --> 0:28:42.820
**IK**
There have been multiple studies that have shown that creativity can enhance tolerance of uncertainty.

0:28:43.920 --> 0:28:46.440
**IK**
So what are your views on this and why?

0:28:48.960 --> 0:28:53.560
**P3**
Yeah, I mean, it definitely applies. I feel like to my own life, I find that so much.

0:28:55.640 --> 0:29:19.520
**P3**
Yeah, I think often there I I see other creative people and I feel that they're just so much more able to deal with these kind of situations because I think what I was mentioning earlier is that because the creative process itself is uncertain and because what you're being able to reflect on those feelings of uncertainty and it makes you sort of feel.

0:29:21.80 --> 0:29:49.920
**P3**
It's comforting. You feel like they're able to process it a lot better. I think generally, just emotional processing, emotional intelligence, being able to amplify, being able to process difficult emotions, I think is so interlinked with being creative, creating artwork, people who I find are, you know, very emotionally intelligent. They are some, some of the most sort of creative people that I know. So I think they're very much interwoven.

0:29:50.310 --> 0:29:54.630
**P3**
And I find it's a way to have personal growth. It's a way to.

0:29:56.670 --> 0:30:21.550
**P3**
Not necessarily become stronger like what we mentioned in the kind of SSE about the difference between resilience and flourishing. So kind of the resilience outlook is you're just trying to have to be strong, you have to power your way through it. But by flourishing and by being oftentimes create, being creative can help with that because you're actually growing from those experiences by reflecting on it by processing your emotions. So I think that's where.

0:30:22.550 --> 0:30:50.870
**P3**
It definitely comes into how how we deal with uncertainty is by going through the creative process and making peace with uncertainty. As I tried to just push it off and try not to think about it or brush it onto the carpet. You're actually confronting it and you're embracing it in a way as opposed to seeing it as something that is negative because uncertainty is a part of life. It's the more we accept it, the more we become at peace with it and the more that we're able to process our emotions and feel dates around it better.

0:30:51.700 --> 0:30:56.340
**IK**
Yeah, thank you. And that's that's really interesting. So thanks.

0:30:58.760 --> 0:31:2.0
**IK**
And now now moving on to kind of creative approaches.

0:31:4.850 --> 0:31:19.50
**IK**
Do you have any? I think I've already. We've already covered this point really, but do you have any examples where a creative approach helped you in engaging with uncertainty? And I think we've already said, but is there anything else you want to add?

0:31:24.20 --> 0:31:27.620
**P3**
Oh gosh, I mean, just so many things. Yeah, I think.

0:31:29.150 --> 0:31:47.990
**P3**
Definitely at that time. So I was, I was on this gap year and I was doing because I didn't get into medicine first time around. So that was already an element of uncertainty. I was like, oh, am I going to get into Med school? What's going on? So I started working and then I it wasn't the best experience. I will say that I had working. It was quite a difficult time.

0:31:49.470 --> 0:31:54.430
**P3**
For a variety of reasons, and I was, you know, at the end of it, I was like, will this even be worth it?

0:31:56.190 --> 0:32:3.870
**P3**
You know, is this isn't good, you know? Is it am I just sort of going through this all for nothing? Will I get into sort of Med school and I think that's when I relied on.

0:32:4.750 --> 0:32:27.550
**P3**
Creativity quite a lot, I think. For me, if more than anything it was realising that I was having because oftentimes I'll be so tired at end of the shift and I think I just want to sleep or I just want to. But the more that I actually reflect in my emotions of the journaling or doing that sort of thing, the more that I felt better and more able to.

0:32:29.610 --> 0:33:2.290
**P3**
Approach life with a more accepting approach and make make peace with it. Then I would say also I rely on my faith quite a lot because that my faith sought to teach me to deal with uncertainty and recognise it as a part of life. I think especially in we live in a very fast-paced society and it's very it can often lead us to like I mentioned have these big goals and we're constantly just go, go, go. We don't have a moment to sit and think and realise that we aren't control of so many aspects of my life and for me.

0:33:2.680 --> 0:33:27.480
**P3**
With my faith, I I sort of see it as everything is kind of more in God's hands, so that also gives me a lot of peace and helps me deal with uncertainty as well. So yeah, I think hopefully, but it's difficult and I think it's easy to say it now, but I'm sure soon I'll come across the situation where it'll be uncertain. I'll be feeling very overwhelmed. But I think as we go through life, there's something to definitely work on.

0:33:28.120 --> 0:33:35.640
**IK**
Yeah, I show because, well, at least now you're flourishing at medical school, so you at least.

0:33:34.820 --> 0:33:37.420
**P3**
Not all the time. It's a it's an up and down process.

0:33:37.80 --> 0:33:45.520
**IK**
Yeah, but I think from the uncertain situation, if you stick with it, you can see the change as you say.

0:34:4.780 --> 0:34:4.980
**P3**
Mm hmm.

0:33:47.160 --> 0:34:7.360
**IK**
But there's one thing you said really at the end was really interesting because that's also something that I think as well. So I'm sure all religions as well. I think the idea is that if you you put faith in, in some in something or someone and you know that. OK.

0:34:11.690 --> 0:34:11.890
**P3**
Mm hmm.

0:34:8.30 --> 0:34:20.590
**IK**
In uncertain situations, they will come to help, or that person will be there for you. So and you also late, like kind of linking that to creativity so.

0:34:22.170 --> 0:34:23.890
**IK**
Is there any difference in the sense of?

0:34:25.640 --> 0:34:27.360
**IK**
The creativity and the religion.

0:34:28.700 --> 0:34:30.100
**IK**
Do both of them help you?

0:34:31.570 --> 0:34:44.250
**IK**
Kind of put your worries on something else so takes that burden away from you worrying about it yourself. Or are they slightly different in in in that attribute?

0:34:47.230 --> 0:34:48.750
**P3**
Yeah, it's a good question.

0:34:50.230 --> 0:34:50.710
**P3**
I think.

0:34:52.30 --> 0:34:53.710
**P3**
To that I suppose.

0:34:55.230 --> 0:35:7.230
**P3**
With the I feel like that normally comes sort of first for me it feels very normal to kind of, you know you you look to your face, you rely on God, that feels very sort of normal and and natural.

0:35:8.470 --> 0:35:11.510
**P3**
But I think when it comes to maybe very specific experiences.

0:35:13.230 --> 0:35:26.950
**P3**
I I may kind of look to or think about feel a little bit more individual than at that point you it might rely on sort of more creative means. And I also think it can sometimes creativity we can almost make a trap for ourselves.

0:35:27.910 --> 0:35:58.670
**P3**
Where by putting expectation on it or we attach it too much to our ego or our self worth, that's when you can actually sort of lose its ability in dealing with uncertainty and dealing with emotions. So I think it's important to keep that balance. I feel like not necessarily over rely on it or trying to like capitalise on it in a way obviously like people who are saying do create who are created in their jobs, it might not necessarily serve that purpose anymore because.

0:35:58.710 --> 0:36:4.390
**P3**
They're using it as a sort of way to either gain money, gain sort of esteem in society.

0:36:4.870 --> 0:36:24.830
**P3**
And that's when it can almost be like a sort of toxic loop, and it loses its the power that it has. So I'd say, yeah, I think it's important to have have that balance with, obviously, you know, for me, it's my faith for it might be created also talking to my fans and family. I didn't all have to coexist together.

0:36:24.850 --> 0:36:28.890
**P3**
To have to keep us grounded and help us deal with in certain situations.

0:36:29.830 --> 0:36:31.270
**IK**
Yeah. Thank you.

0:36:33.80 --> 0:36:40.360
**IK**
And I think that the bit you were just saying at the end now I think leads on to the other part of I'll ask that question next.

0:36:41.880 --> 0:36:58.560
**IK**
About kind of unhelpfulness. So I think you just talked slightly on the idea of how creativity. Do you think it can be unhelpful because you were just saying now if I'm right that it can be kind of abused in a way or?

0:37:1.220 --> 0:37:3.620
**IK**
Used in a way to gain attention.

0:37:5.140 --> 0:37:10.980
**IK**
With your own self esteem as you see. So do you think creativity can ever be?

0:37:12.10 --> 0:37:12.450
**P3**
Oh.

0:37:12.460 --> 0:37:15.260
**IK**
Unhelpful in clinical practise.

0:37:16.360 --> 0:37:44.320
**P3**
Yeah, it's interesting because we were having this discussion during the SSC where we were thinking about if something like we did was compulsory, would it still have the same value if we're forcing people to be creative? Does it take away the meaning and the power behind it? And I think it's already interesting discussion to have because at the same time you want to push people to think outside the box, to do things differently. But if they feel that that's.

0:37:45.360 --> 0:37:45.920
**P3**
In some way.

0:37:46.170 --> 0:37:50.570
**P3**
They feel it might be too much or a bit toxic. That's definitely not something that we want.

0:37:52.370 --> 0:38:7.210
**P3**
I think it's hard, yeah, to balance those. Those two sides of it. I think for me, I think back to my time, I remember doing like gccr for example and that's when like I remember, I really enjoyed like painting, enjoying and all of that. But when it.

0:38:9.220 --> 0:38:10.740

**P3**
So yeah, I was just thinking about some of my my time in school when I was doing it for and it almost became it lost that. The beauty of it was almost lost because I was thick. I'm to get a good grade. And you when you start tying it to yourself worth and you know a future sort of I need to get a good grade in this for you know you me and it just it becomes part of.

0:38:52.250 --> 0:38:56.610
**P3**
How we yeah, like trying to see. How does this benefit me?

0:38:58.130 --> 0:38:59.10
**P3**
I'm seeing it as a more.

0:38:59.330 --> 0:39:17.50
**P3**
A way to just achieve something that we want, but it just becomes a means to an end. That's when the creative process lost when we just enjoy the process for the sake of it. I think that's when all the that's when you get the self reflection and the sense of like being able to.

0:39:18.690 --> 0:39:29.690
**P3**
I guess yeah, reflection being able to process emotions by focusing on the actual method itself as opposed to seeing how can this art do expertly? How can this, you know?

0:39:30.210 --> 0:39:42.370
**P3**
I think it's coming hard. You know, we live in an age of sort of social media capitalism. Everything has like a means to an end. So I think it's important to hold on to the secretness of the creative process in a very fast-paced society.

**IK**
I think we've already said about how it might be helpful in in clinical practice. So is there anything else you wanted to say on the helpful side?

0:40:8.710 --> 0:40:8.790
**IK**
Or.

0:40:9.970 --> 0:40:35.810
**P3**
Yeah, I think we touched on it. So I think like firstly helping us reflect on our experiences as like, especially as doctors you go through so much emotionally, physically, it can mean incredibly training. And if you're gonna not taking care of yourself or being able to process your emotions and experiences, it's going to take a toll. Eventually I feel for sure also just as a way to relax and also remind yourself.

0:40:36.690 --> 0:40:52.450
**P3**
You are an individual, especially in the NHS. If we think about how we don't want to sort whatever we make into a bit of a van, but a lot of doctors are not necessarily being appreciated, not just pay wise, but the working conditions are not great to be honest.

0:40:53.650 --> 0:41:2.170
**P3**
So it can often make you feel you're not really an individual, you're just kind of a number. You're just one part of the machinery in this massive sort of organisation. So.

0:41:3.10 --> 0:41:29.730
**P3**
I think it can make you recognise you still feel like an individual again because you might make no one will be able. No one can make the same art as you. No one can. You can't be the same art that you make can't be replaced by someone else. It's so unique. So I think that is also a good, important reminder. I know for so many people. It's also a way to kind of just relax, like like that meditative process. Like I mentioned before. So there's so many facets of it, I feel like.

0:41:30.970 --> 0:41:31.450
**P3**
That can be important.

**IK**
Is there anything else you want to add?

**P3**
I think I forgot to mention particularly with regards to the SSC, I think there’s so much value in the fact that we were able to create things together as a group and the fact that I actually had the realisation that, ”Oh, everyone's going through this feeling of uncertainty. Everyone is kind of going through that same process”.

Connecting together as we were making the art, it makes sense why, art therapies work for patients because they're able to connect in this way. So I think incorporating that in some way into the curriculum would be really, really amazing to see.

**IK**
How do you think we could bring it into the curriculum?

**P3**
Yeah, I think you know I I think definitely incorporating it in a bigger way. I was even thinking I think I can't remember who it was that mentioned it. One of the people we met on the SSC is one of the doctors was talking about how they were essentially proposing a programme more throughout the year. So like every two weeks or something where they getting medical students to engage in artwork, even going to museums, discussing art, doing that more kind of putting that creative hat on, doing more of the critical thinking.

0:45:55.150 --> 0:46:25.190
**P3**
To incorporate that I think is really nice at the minute we have, there are aspects of the course which are valuable in their own way, but I know some students are not very keen on we have, I don't know if you've heard, we have like team based learning this sort of thing and people are thinking I know we were discussing in the SSC that we thought it would be more valuable if we were instead looking a bit more towards humanities. I know in other unities even my sister. So she went to Birmingham to study medicine and they had a module more looking at ethics.

0:46:25.350 --> 0:46:25.750
**P3**
Philosophy.

0:46:26.270 --> 0:46:58.310
**P3**
Digging into even artwork that they had various sort of artworks on like death and mortality, and they had to reflect on them. How does this apply to patients? I think going through that, I think it has so much value and I think what we don't want to do is that when we become doctors, we lose that ability to think with that sort of perspective because it is so integral to having empathy for our patients and just not accepting not that's necessary accepting things for the way they are.

0:46:58.350 --> 0:46:58.630
**P3**
Being critical.

0:46:59.670 --> 0:47:15.550
**P3**
Being reflective. So yeah, I think maybe having more of a long process throughout the MBBS, even if it's just like once a month I think has so much value and it will just help enrich people and ultimately make well-rounded doctors overall.
